# Modification of Natural Eudesmane Scaffolds via Mizoroki-Heck Reactions

**DOI:** 10.3390/molecules22040652

**Published:** 2017-04-20

**Authors:** Mohamed Zaki, Mohamed Akssira, Sabine Berteina-Raboin

**Affiliations:** 1 Institut de Chimie Organique et Analytique, University d’Orléans, UMR CNRS 7311, B.P. 6759, 45067 Orléans CEDEX 2, France; zaki.smc@gmail.com; 2 Laboratoire de Chimie Physique & Chimie Bioorganique, Département de chimie, URA C 22, Pôle RéPAM, F. S. T. University Hassan II de Casablanca, B.P. 146 Yasmina, 28800 Mohammedia, Morocco; akssira@fstm.ac.ma

**Keywords:** eudesmane, isocostic and ilicic acids, *Dittrichia viscosa* L. Greuter, sesquiterpenes, Mizoroki-Heck reaction

## Abstract

The Mizoroki-Heck reaction was applied to substrates derived from isocostic and ilicic acids, important sesquiterpene components of *Dittrichia viscosa* L. Greuter that were extracted directly from plant material collected in Morocco. After optimization of the metallo-catalysis conditions, various aryl-groups were successfully introduced on the exocyclic double bond with an exclusive *E*-configuration and without racemization.

## 1. Introduction

**Figure 1:**
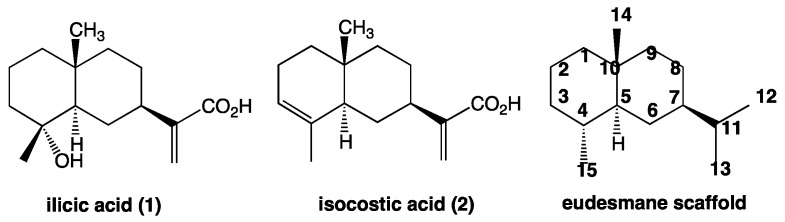
Isocostic and ilicic acids as eudesmane scaffolds.

Natural products are a well-known continuous source of inspiration for the design of new bioactive agents with applications in the therapeutic, cosmetic or agricultural areas [1]. They can serve as building blocks for the synthesis of more complex bioactive compounds [2]. Based on traditional use, numerous plants of medicinal interest have been identified and conventional drugs developed from their extracts [3,4]. As part of our program studying Moroccan plants [5,6], our interest is focused on *Dittrichia viscosa* L. Greuter [7,8], an invasive perennial plant particularly abundant in wasteland areas. Its extract is used in traditional Moroccan medicine for its antipyretic, antiseptic and anti-inflammatory properties [9,10], and also exhibits antifungal activity [11,12,13]. Recent studies reported that the aerial parts of the plant are a rich source of eudesmane sesquiterpenes, among which ilicic acid (**1**) and isocostic acid (**2**) (Figure 1) represent up to 2.5% and 2% of the aerial part dry weight, respectively [5]. This plant, like others, could represent a renewable source of enantiopure compounds to obtain diversified libraries of products of interest.

Indeed, sesquiterpene derivatives have attracted considerable attention lately [14] due to their pharmacological and phytochemical activities, in particular sesquiterpene lactones [15,16,17] which have already been submitted to various structural modifications, including the Mizoroki-Heck reaction [18,19,20,21,22,23]. However, only a few modifications have been reported for analogues such as eudesmane-carboxylic acids, aldehydes or eudesmanols, and none included modifications of the exocyclic alkene part [24,25,26]. In view of the great biological potential of ilicic and isocostic acid derivatives [27,28,29,30], we decided to apply the Mizoroki-Heck cross-coupling reaction to these natural compounds to generate some novel eudesmane analogues. We report herein the behavior of these compounds extracted from plants in palladium-catalyzed reactions [31,32,33,34].

## 2. Results and Discussion

### 2.1. Optimization of the Mizoroki-Heck Reaction on Ilicic Acid

**Scheme 1:**
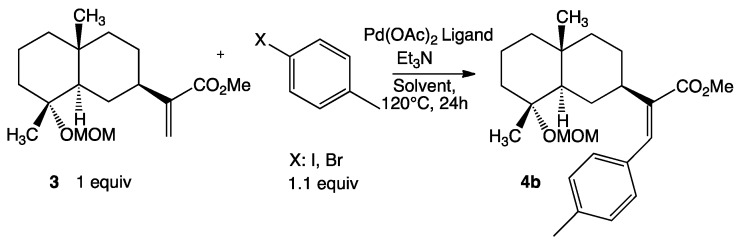
Mizoroki-Heck reaction of ilicic acid.

We first examined the behavior of ilicic acid methyl ester in a model Mizoroki-Heck reaction (Scheme 1). Unfortunately only starting material was recovered [20]. Protection of the alcohol was then considered and the methoxymethyl-ether (MOM) group was selected as the most efficient protecting group allowing the isolation of compound **3** in 60% yield. With **3** as starting material, compound **4b** was then obtained in 30% yield in presence of Pd(OAc)_2_ (0.1 equiv), *p*-tolyl iodide (1.1 equiv) and Et_3_N (3 equiv) in *N*,*N*-dimethylformamide (DMF) (Table 1, entry 1). The use of silver acetate as oxidizing agent and base offered no significant advantages (entry 2) [35,36]. Next a bulky electron-rich phosphine, tri(*o*-tolyl)phosphine was used, enabling the isolation of **4b** in 65% yield, and acetonitrile was used to replace DMF as solvent, but a reduced yield was noted (entry 5) [21,22]. An increase in the catalytic system loading failed to improve this result (entry 6). The optimized conditions were as follows: **3** (1 equiv), aryl iodide (1.1 equiv), Et_3_N (3 equiv), Pd(OAc)_2_ (0.1 equiv) and P(*o*-Tol)_3_ (0.1 equiv) in DMF at 120 °C for 24 h. These conditions were extended to aryl bromides without any significant loss of reactivity (entry 8).

### 2.2. Substrate Scope and Deprotection

**Scheme 2:**
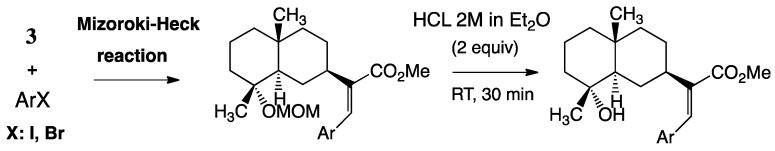
MOM deprotection conditions.

Various aryl iodides and bromides were then used to generate a library of eudesmane analogues using the optimized cross-coupling reaction conditions (Scheme 2 and Table 2). The reactions were clean and the expected products were synthesized in good yields. As expected, the palladium coupling reaction tolerated different aromatic halides bearing electron-donating (Me, OMe) and withdrawing groups (F, CO_2_Me, CHO) in the *ortho*, *meta* and *para*-positions (compounds **4a–i**). Then, sesquiterpenes **5a**–**i** were rapidly generated under acidic conditions in good to excellent yields. When the fluoro substituent was in ortho position, deprotection occurred simultaneously with the Mizoroki-Heck reaction, compound **4g** was never observed and **5g** was isolated in one step in 62% yield (entry 7). The methodology was also extended to heterocyclic derivatives and the desired product **4i** was isolated with good yield (entry 9).

**Figure 2:**
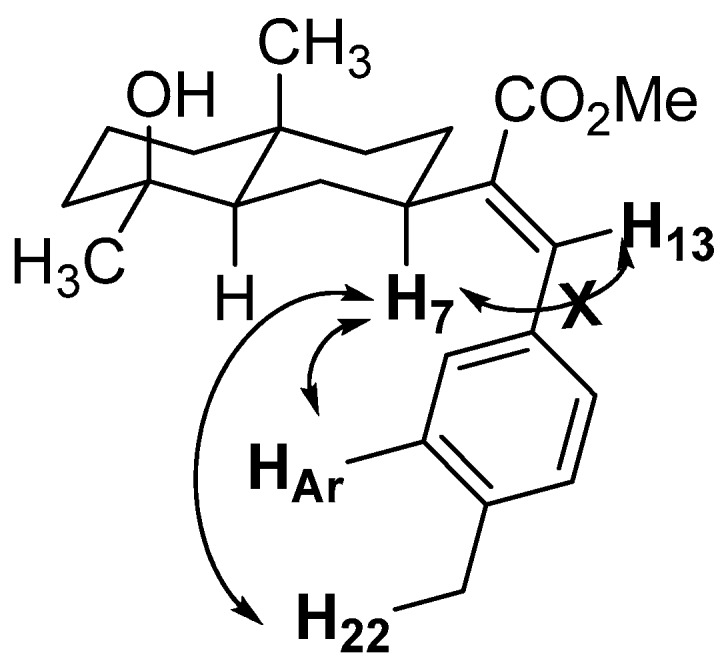
NOESY-NMR experiment of **5b**.

A Nuclear Overhauser Effect Spectroscopy (NOESY)**-**NMR experiment on **5b** allowed us to determine the double bond configuration. Interactions between H_7_-H_Ar_ and H_7_–H_22_ involved an *E*-olefin geometry emphasized by the absence of signals between H_7_ and H_13_ (Figure 2). Our stereochemical result is in accordance with the work reported by Colby and co-workers [20].

### 2.3. Mizoroki-Heck Reaction on Esterified Isocostic Acid

**Scheme 3:**
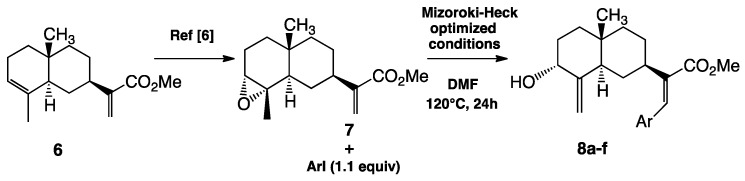
Epoxidation and Mizoroki-Heck reaction on esterified isocostic acid.

Next we focused on the other major constituent found on the acidified dichloromethane (DCM) extracts of *Dittrichia viscosa* L. Greuter. Isocostic acid (**2**) was esterified and submitted to our optimized Mizoroki-Heck conditions leading to a complex mixture of products. When an epoxidation was carried out on esterified compound **6** in order to prevent the migration of the endocyclic double bond, the epoxide **7** was obtained as a unique enantiomer (Scheme 3) [6].

**Figure 3:**
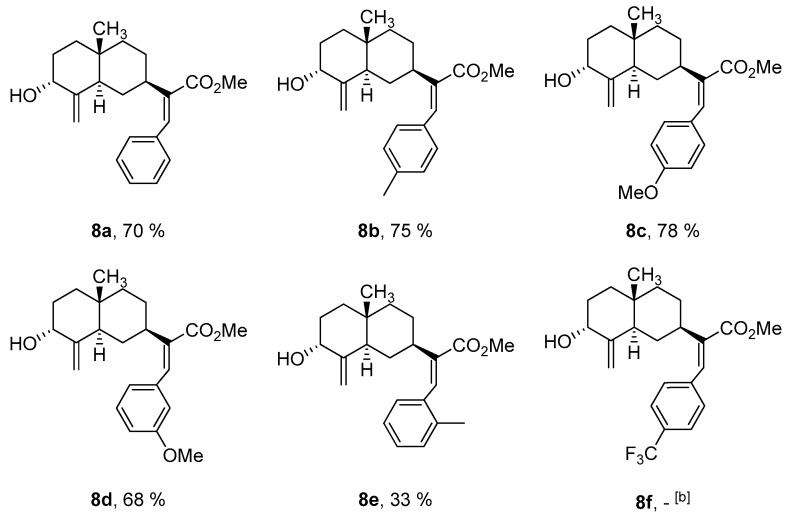
Mizoroki-Heck reaction on esterified isocostic acid.

After the cross-coupling reaction, a new double bond signal appeared both in ^1^H-NMR (δ 4.57 and δ 4.91 ppm) and ^13^C-NMR (δ 109.4 (C-15) and δ 151.7 ppm (C-4)). The Distortionless Enhancement by Polarization Transfer (DEPT) experiment confirmed the exocyclic position of this unsaturation. Furthermore, a significant shielding effect was observed for H_3_ in ^1^H-NMR (δ 2.86 vs. 4.27 ppm) and C_3_ in ^13^C-NMR (δ 61.1 vs. 73.8 ppm), which corroborates the formation of an allylic alcohol from the epoxide ring opening. The double bond activated by the presence of the ester in the α-position reacted in the Mizoroki-Heck reaction giving access chemoselectively to **8a** in 70% yield (Figure 3).

**Scheme 4:**
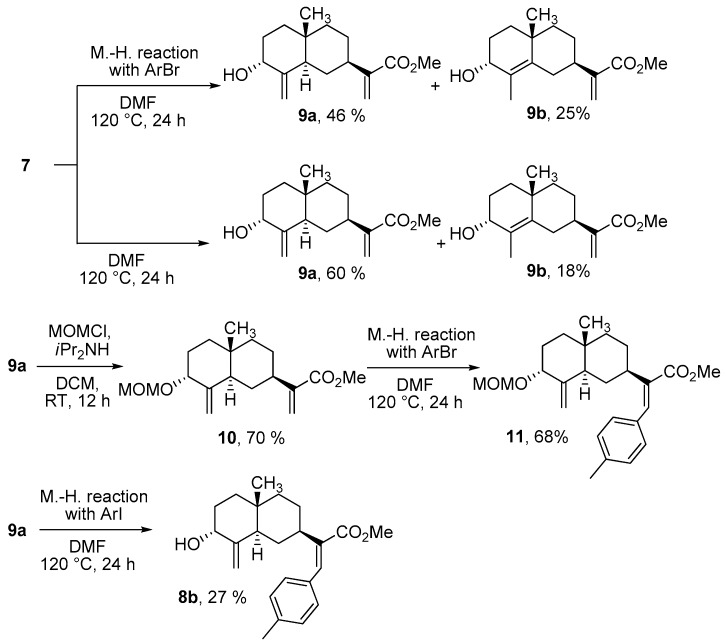
Control experiments.

The reaction was successfully performed with *ortho*-, *meta*- and *para*-aryl iodides substituted with electron donating groups (compounds **8a**–**e**). However, the steric hindrance of aryl iodides substituted in the *ortho*-position had a negative effect on the transformation, as compound **8e** was isolated only in 33% yield. When an electron-deficient aryl iodide was used, only traces of the expected product **8f** were obtained. Unfortunately, aryl bromides proved incompatible with our optimized conditions as only the opening of the epoxide was observed (Scheme 4).

The lack of reactivity of the aryl bromide was confirmed by heating **7** in DMF in the absence of the other reagents. This experiment showed the formation of **9a** and **9b** in almost the same proportions. The isolation of **9a** and **9b** was possible by column chromatography and each compound was fully characterized by comparing their spectroscopic data with the literature [6,37]. Further investigation was then conducted on the major product **9a** resulting from the epoxide ring opening. The protection of the alcohol **9a** was made as before with a MOM group leading to compound **10** then cross coupling conditions were applied and expected compound **11** was obtained in good yield. When **9a** was submitted directly to the cross coupling reaction with a more reactive aryl iodide, only 27% of **8b** was isolated along with degradation products, indicating that our cross-coupling conditions are not compatible with the presence of an unprotected alcohol. For this other major constituent found on the acidified DCM extracts of *Dittrichia viscosa* L. Greuter, the configuration of the trisubstituted double bond synthetized was examined though the correlations obtained by the NOESY-NMR experiment. A spatial correlation was observed between the aromatic proton and H_7_ when no signal was noticed between H_7_ and H_13_ led us conclude on the *E*-olefin geometry.

## 3. Materials and Methods

### 3.1. General Methods

All reagents were purchased from commercial suppliers and were used without further purification except for DMF, which was stored under argon and activated molecular sieves. The reactions were monitored by thin-layer chromatography (TLC) analysis using silica gel (60 F254) plates. Compounds were visualized by UV irradiation. Flash column chromatography was performed on silica gel 60 (230–400 mesh, 0.040–0.063 mm). ^1^H-NMR and ^13^C-NMR spectra were recorded on Avance spectrometers (Bruker, France, SAS) at 250.13 MHz (^13^C, 62.9 MHz) or 400.13 MHz (^13^C, 100.62 MHz). Chemical shifts are given in parts per million from tetramethylsilane (TMS) as internal standard. The following abbreviations are used for the proton spectra multiplicities: s: singlet, d: doublet, t: triplet, q: quartet, qt: quintuplet, m: multiplet. Coupling constants (*J*) are reported in Hertz (Hz). Multiplicities were determined by DEPT-135 sequences. Attributions of protons and carbons were made with the help of Heteronuclear Single Quantum Correlation (HSQC) and Heteronuclear Multiple Bond Correlation (HMBC) 2D NMRs. Eudesmane numbering of carbons was used instead of the IUPAC numbering. High-resolution mass spectra (HRMS) were performed on a Maxis 4G instrument (Bruker, France, SAS).

### 3.2. Procedure for the Synthesis of Methyl-(2E)-2-[(2R,4aR,8R,8aR)-8-(methoxymethoxy)-4a,8-dimethyl-decahydronaphthalen-2-yl]prop-2-enoate (***3***)

To a solution of methyl 2-((2*R*,4a*R*,8*R*,8a*R*)-8-hydroxy-4a,8-dimethyldecahydronaphthalen-2-yl) acrylate (126 mg, 2.25 mmol, 1 equiv) in anhydrous CH_2_Cl_2_ (10 mL) were added DPA (1.56 mL, 9.02 mmol, 4 equiv) and MOMCl (0.68 mL, 9.02 mmol, 4 equiv). The reaction mixture was stirred for 12 h at room temperature under argon. Then water (10 mL) was added and the mixture was extracted with CH_2_Cl_2_ (3 × 10 mL). The organic layers were dried (MgSO_4_), filtered and concentrated under reduced pressure to give the desired product **3** (419 mg, 1.35 mmol, 60% yield) after purification by flash chromatography on a silica gel column (petroleum ether/EtOAc:90/10). Colorless oil, [α]${}_{D}^{20}$ −39.5 (*c* 1.0, CH_2_Cl_2_). ^1^H-NMR (250 MHz, CDCl_3_): *δ* = 6.10 (d, *J* = 1.2 Hz, 1H, H-13), 5.53 (dd, *J* = 1.2, 1.2 Hz, 1H, H-13), 4.69 (dd, *J* = 7.4, 6.0 Hz, 2H, OCH_2_), 3.73 (s, 3H, OCH_3_), 3.32 (s, 3H, OCH_3_), 2.51 (dddd, *J* = 12.7, 11.7, 4.4, 3.2 Hz, 1H, H-7), 1.96–1.74 (m, 2H), 1.68–1.17 (m, 10H), 1.17–1.01 (m, 4H), 0.92 (s, 3H, H-14) ppm. ^13^C-NMR (63 MHz, CDCl_3_) δ 168.0 (C-12), 146.1 (C-11), 122.5 (C-13), 90.1 (OCH_2_), 78.2 (C-4), 55.2 (OCH_3_), 52.6 (OCH_3_), 51.8 (C-5), 45.1 (CH_2_), 40.9 (CH_2_), 40.6 (C-7), 39.2 (CH_2_), 34.7 (C-10), 27.7 (CH_2_), 26.7 (CH_2_), 19.9 (CH_2_), 19.7 (C-15), 19.3 (C-14) ppm. HRMS (ESI): *m*/*z* [M + Na]^+^ calcd. for C_18_H_30_NaO_4_ 333.2036; found 333.2036.

### 3.3. General Procedure for the Synthesis of Compounds ***4a***–***4i***

A solution of substrate **3** (1 equiv, 100 mg), Pd(OAc)_2_, (0.1 eqiv) and an appropriate haloaryl compound (1.1 eqiv) in the presence of triethylamine (3 equiv) and P(*o*-Tol)_3_ (0.1 equiv) in DMF (2 mL) was stirred for 24 h at 120 °C. After cooling, water (10 mL) was added to the reaction mixture which is extracted with EtOAc (3 × 10 mL). The combined organic layers were washed with water (2 × 30 mL), dried (MgSO_4_), filtered and concentrated under reduce pressure. Expected compounds were obtained after purification by flash chromatography on silica gel (petroleum ether/EtOAc:90/10).

#### 3.3.1. Methyl-(2*E*)-2-[(2*R*,4a*R*,8*R*,8a*R*)-8-(methoxymethoxy)-4a,8-dimethyl-decahydronaphthalen-2-yl]-3-phenylprop-2-enoate (**4a**)

Colorless oil, [α]${}_{D}^{20}$ −42.7 (*c* 1.0, CH_2_Cl_2_). ^1^H-NMR (400 MHz, CDCl_3_): *δ* = 7.57 (s, 1H, H-13), 7.42–7.25 (m, 5H, H_Ar_), 4.67 (s, 2H, OCH_2_), 3.81 (s, 3H, OCH_3_), 3.28 (s, 3H, OCH_3_), 2.86 (dddd, *J* = 12.3, 12.2, 4.0, 3.8 Hz, 1H, H-7), 2.27–2.13 (m, 1H), 1.94 (dd, *J* = 12.5, 12.5 Hz, 1H), 1.89–1.80 (m, 1H), 1.75–1.66 (m, 1H), 1.61–1.44 (m, 4H), 1.44–1.23 (m, 5H), 1.15 (s, 3H, H-15), 1.03 (s, 3H, H-14) ppm. ^13^C-NMR (101 MHz, CDCl_3_): *δ* = 168.6 (C-12), 138.7 (C-13), 138.0 (C-11), 136.1 (C_Ar_), 129.0 (2CH_Ar_), 128.5 (2CH_Ar_), 128.2 (CH_Ar_), 90.1 (OCH_2_), 78.3 (C-4), 55.1 (OCH_3_), 52.8 (OCH_3_), 51.7 (C-5), 45.0 (CH_2_), 40.9 (CH_2_), 39.4 (CH_2_), 39.1 (C-7), 34.7 (C-10), 26.0 (CH_2_), 25.5 (CH_2_), 19.9 (CH_2_), 19.7 (C-15), 19.4 (C-14) ppm. HRMS (ESI): *m*/*z* [M + Na]^+^ calcd. for C_24_H_34_NaO_4_ 409.2348; found 409.2349.

#### 3.3.2. Methyl-(2*E*)-2-[(2*R*,4a*R*,8*R*,8a*R*)-8-(methoxymethoxy)-4a,8-dimethyl-decahydronaphthalen-2-yl]-3-(4-methylphenyl)prop-2-enoate (**4b**)

Colorless oil, [α]${}_{D}^{20}$ +68.3 (*c* 1.0, CH_2_Cl_2_). ^1^H-NMR (400 MHz, CDCl_3_): *δ* = 7.55 (s, 1H, H-13), 7.21 (d, *J* = 8.2 Hz, 2H, H_Ar_), 7.18 (d, *J* = 8.2 Hz, 2H, H_Ar_), 4.68 (s, 2H, OCH_2_), 3.80 (s, 3H, OCH_3_), 3.29 (s, 3H, OCH_3_), 2.88 (dddd, *J* = 12.2, 12.2, 4.1, 4.0 Hz, 1H, H-7), 2.37 (s, 3H), 2.28–2.14 (m, 1H), 1.96 (dd, *J* = 12.5, 12.4 Hz, 1H), 1.87–1.80 (m, 1H), 1.76–1.67 (m, 1H), 1.58–1.45 (m, 3H), 1.45–1.29 (m, 5H), 1.22–1.11 (m, 4H), 1.03 (s, 3H, H-14) ppm. ^13^C-NMR (101 MHz, CDCl_3_): *δ* = 168.6 (C-12), 138.7 (C_Ar_), 138.2 (C-13), 137.3 (C-11), 133.2 (C_Ar_), 129.2 (2CH_Ar_), 129.1 (2CH_Ar_), 90.1 (OCH_2_), 78.3 (C-4), 55.1 (OCH_3_), 52.8 (OCH_3_), 51.6 (C-5), 45.0 (CH_2_), 40.9 (CH_2_), 39.4 (CH_2_), 39.0 (C-7), 34.7 (C-10), 25.9 (CH_2_), 25.5 (CH_2_), 21.4 (C-15), 19.9 (CH_2_), 19.7 (CH_3_), 19.4 (C-14) ppm. HRMS (ESI): *m*/*z* [M + Na]^+^ calcd. for C_25_H_36_NaO_4_ 423.2506; found 423.2505.

#### 3.3.3. Methyl-(2*E*)-2-[(2*R*,4a*R*,8*R*,8a*R*)-8-(methoxymethoxy)-4a,8-dimethyl-decahydronaphthalen-2-yl]-3-(4-methoxyphenyl)prop-2-enoate (**4c**)

Colorless oil, [α]${}_{D}^{20}$ +86.0 (*c* 1.0, CH_2_Cl_2_). ^1^H-NMR (400 MHz, CDCl_3_): *δ* = 7.52 (s, 1H, H-13), 7.27 (d, *J* = 8.5 Hz, 2H, H_Ar_), 6.90 (d, *J* = 8.5 Hz, 2H, H_Ar_), 4.68 (s, 2H, OCH_2_), 3.83 (s, 3H, OCH_3_), 3.79 (s, 3H, OCH_3_), 3.29 (s, 3H, OCH_3_), 2.90 (dddd, *J* = 12.3, 12.0, 4.2, 4.0 Hz, 1H, H-7), 2.22 (dddd, *J* = 13.7, 12.8, 12.8, 3.8 Hz, 1H), 1.96 (dd, *J* = 12.4, 12.4 Hz, 1H), 1.88–1.85 (m, 1H), 1.76–1.66 (m, 1H), 1.64–1.45 (m, 4H), 1.44–1.30 (m, 4H), 1.24–1.12 (m, 4H), 1.04 (s, 3H, H-14) ppm. ^13^C-NMR (101 MHz, CDCl_3_): *δ* = 168.7 (C-12), 159.7 (C_Ar_), 138.4 (C-13), 136.3 (C-11), 130.8 (2CH_Ar_), 128.5 (C_Ar_), 114.0 (2CH_Ar_), 90.2 (OCH_2_) , 78.4 (C-4), 55.5 (OCH_3_), 55.2 (OCH_3_), 52.9 (OCH_3_), 51.6 (C-5), 45.1 (CH_2_), 40.9 (CH_2_), 39.5 (CH_2_), 39.0 (C-7), 34.8 (C-10), 25.9 (CH_2_), 25.5 (CH_2_), 19.9 (CH_2_), 19.7 (C-15), 19.4 (C-14) ppm. HRMS (ESI): *m*/*z* [M + Na]^+^ calcd. for C_25_H_36_NaO_5_ 439.2455; found 439.2454.

#### 3.3.4. Methyl-(2*E*)-2-[(2*R*,4a*R*,8*R*,8a*R*)-8-(methoxymethoxy)-4a,8-dimethyl-decahydronaphthalen-2-yl]-3-(4-formylphenyl)prop-2-enoate (**4d**)

Colorless oil, [α]${}_{D}^{20}$ +14.3 (*c* 1.0, CH_2_Cl_2_). ^1^H-NMR (400 MHz, CDCl_3_): *δ* = 10.02 (s, 1H, CHO), 7.89 (d, *J* = 8.0 Hz, 2H, H_Ar_), 7.56 (s, 1H, H-13), 7.45 (d, *J* = 7.9 Hz, 2H, H_Ar_), 4.66 (s, 2H, OCH_2_), 3.82 (s, 3H, OCH_3_), 3.28 (s, 3H, OCH_3_), 2.77 (dddd, *J* = 12.3, 11.8, 3.7, 3.7 Hz, 1H, H-7), 2.20 (qd, *J* = 13.1, 3.9 Hz, 1H), 1.94 (dd, *J* = 12.7, 12.3 Hz, 1H), 1.88–1.80 (m, 1H), 1.76–1.67 (m, 1H), 1.61 (s, 1H), 1.57–1.19 (m, 8H), 1.14 (s, 3H, H-15), 1.02 (s, 3H, H-14) ppm. ^13^C-NMR (101 MHz, CDCl_3_): *δ* = 191.8 (CHO), 168.1 (C-12), 142.4 (C_Ar_), 140.3 (C_Ar_), 137.0 (C-13), 135.7 (C-11), 129.9 (2CH_Ar_), 129.6 (2CH_Ar_), 90.2 (OCH_2_), 78.3 (C-4), 55.2 (OCH_3_), 52.8 (OCH_3_), 51.9 (C-5), 44.8 (CH_2_), 40.8 (CH_2_), 39.5 (C-7), 39.4 (CH_2_), 34.7 (C-10), 26.0 (CH_2_), 25.6 (CH_2_), 19.8 (CH_2_), 19.7 (C-15), 19.4 (C-14) ppm. HRMS (ESI): *m*/*z* [M + Na]^+^ calcd. for C_25_H_34_NaO_5_ 437.2294; found 437.2298.

#### 3.3.5. Methyl-(2*E*)-2-[(2*R*,4a*R*,8*R*,8a*R*)-8-(methoxymethoxy)-4a,8-dimethyl-decahydronaphthalen-2-yl]-3-(4-fluorophenyl)prop-2-enoate (**4e**)

White solid, m.p. 126–128 °C. [α]${}_{D}^{20}$ +55.0 (*c* 1.0, CH_2_Cl_2_). ^1^H-NMR (400 MHz, CDCl_3_): *δ* = 7.51 (s, 1H, H-13), 7.32–7.23 (m, 2H, H_Ar_), 7.06 (dd, *J* = 8.4, 8.4 Hz, 2H, H_Ar_), 4.67 (s, 2H, OCH_2_), 3.80 (s, 3H, OCH_3_), 3.29 (s, 3H, OCH_3_), 2.80 (dddd, *J* = 12.2, 12.1, 4.2, 4.1 Hz, 1H, H-7), 2.20 (qd, *J* = 13.6, 13.0, 4.2 Hz, 1H), 1.94 (dd, *J*= 12.5, 12.4 Hz, 1H), 1.87–1.81 (m, 1H), 1.73–1.65 (m, 1H), 1.60 (s, 1H), 1.58–1.44 (m, 3H), 1.44–1.23 (m, 5H), 1.15 (s, 3H, H-15), 1.03 (s, 3H, H-14) ppm. ^13^C-NMR (101 MHz, CDCl_3_): *δ* = 168.4 (C-12), 162.5 (d, *J*= 248.4 Hz, C_Ar_), 138.0 (C-13), 137.5 (C-11), 132.1 (d, *J*= 3.4 Hz, C_Ar_), 130.9 (d, *J*= 8.1 Hz, 2CH_Ar_), 115.6 (d, *J*= 21.5 Hz, 2CH_Ar_), 90.2 (OCH_2_), 78.3 (C-4), 55.2 (OCH_3_), 52.9 (OCH_3_), 51.7 (C-5), 45.0 (CH_2_), 40.9 (CH_2_), 39.4 (CH_2_), 39.1 (C-7), 34.8 (C-10), 25.9 (CH_2_), 25.5 (CH_2_), 19.9 (CH_2_), 19.7 (C-15), 19.4 (C-14) ppm. HRMS (ESI): *m*/*z* [M + Na]^+^ calcd. for C_24_H_33_FNaO_4_ 427.2255; found 427.2255.

#### 3.3.6. Methyl-(2*E*)-2-[(2*R*,4a*R*,8*R*,8a*R*)-8-(methoxymethoxy)-4a,8-dimethyl-decahydronaphthalen-2-yl]-3-(3-methoxyphenyl)prop-2-enoate (**4f**)

Colorless oil, [α]${}_{D}^{20}$ +92.0 (*c* 1.0, CH_2_Cl_2_). ^1^H-NMR (400 MHz, CDCl_3_): *δ* = 7.55 (s, 1H, H-13), 7.31–7.24 (m, 1H, H_Ar_), 6.91–6.82 (m, 3H, H_Ar_), 4.70–4.63 (m, 2H, OCH_2_), 3.82 (s, 3H, OCH_3_), 3.81 (s, 3H, OCH_3_), 3.28 (s, 3H, OCH_3_), 2.88 (dddd, *J*= 12.5, 12.3, 3.5, 3.5 Hz, 1H, H-7), 2.19 (dddd, *J* = 13.0, 13.0, 13.0, 3.6 Hz, 1H), 1.95 (dd, *J* = 12.5, 12.5 Hz, 1H), 1.89–1.82 (m, 1H), 1.78–1.70 (m, 2H), 1.61 (s, 1H), 1.56–1.51 (m, 1H), 1.51–1.43 (m, 1H), 1.43–1.31 (m, 4H), 1.31–1.23 (m, 1H), 1.16 (s, 3H, H-15), 1.03 (s, 3H, H-14) ppm. ^13^C-NMR (101 MHz, CDCl_3_): *δ* = 168.5 (C-12), 159.6 (C_Ar_), 138.6 (C-13), 138.2 (C-11), 137.4 (C_Ar_), 129.5 (CH_Ar_), 121.5 (CH_Ar_), 114.3 (CH_Ar_), 113.9 (CH_Ar_), 90.2 (OCH_2_), 78.3 (C-4), 55.4 (OCH_3_), 55.1 (OCH_3_), 53.0 (OCH_3_), 51.7 (C-5), 45.0 (CH_2_), 40.9 (CH_2_), 39.5 (CH_2_), 39.2 (C-7), 34.7 (C-10), 25.9 (CH_2_), 25.6 (CH_2_), 19.9 (CH_2_), 19.5 (C-15), 19.4 (C-14) ppm. HRMS (ESI): *m*/*z* [M + Na]^+^ calcd. for C_25_H_36_NaO_5_ 439.2452; found 439.2454.

#### 3.3.7. Methyl-2-[(1*E*)-2-[(2*R*,4a*R*,8*R*,8a*R*)-8-(methoxymethoxy)-4a,8-dimethyl-decahydronaphthalen-2-yl]-3-methoxy-3-oxoprop-1-en-1-yl]benzoate (**4h**)

Colorless oil, [α]${}_{D}^{20}$ +107.3 (*c* 1.0, CH_2_Cl_2_). ^1^H-NMR (400 MHz, CDCl_3_): *δ* = 8.03 (dd, *J* = 7.9, 1.4 Hz, 1H, H_Ar_), 7.99 (s, 1H, H-13), 7.51 (td, *J* = 7.6, 1.4 Hz, 1H, H_Ar_), 7.42–7.35 (m, 1H, H_Ar_), 7.25–7.19 (m, 1H, H_Ar_), 4.63 (s, 2H, OCH_2_), 3.86 (s, 3H, OCH_3_), 3.81 (s, 3H, OCH_3_), 3.27 (s, 3H, OCH_3_), 2.47 (tt, *J* = 12.1, 4.2 Hz, 1H, H-7), 2.21–2.06 (m, 1H), 1.92–1.75 (m, 2H), 1.67–1.56 (m, 2H), 1.54–1.37 (m, 3H), 1.31 (ddt, *J* = 12.1, 8.6, 4.0 Hz, 3H), 1.23–1.13 (m, 2H), 1.11 (s, 3H, H-15), 0.97 (s, 3H, H-14) ppm. ^13^C-NMR (101 MHz, CDCl_3_): *δ* = 168.1 (C-12), 167.0 (CO_2_Me), 140.1 (C-13), 138.4 (C-11), 136.2 (C_Ar_), 132.2 (CH_Ar_), 130.8 (CH_Ar_), 129.7 (CH_Ar_), 129.0 (C_Ar_), 128.0 (CH_Ar_), 90.1 (OCH_2_), 78.3 (C-4), 55.1 (OCH_3_), 52.5 (OCH_3_), 52.2 (OCH_3_), 51.7 (C-5), 44.9 (CH_2_), 40.8 (CH_2_), 39.6 (C-7), 39.3 (CH_2_), 34.7 (C-10), 26.0 (CH_2_), 25.5 (CH_2_), 19.8 (CH_2_), 19.8 (C-15), 19.3 (C-14) ppm. HRMS (ESI): *m*/*z* [M + Na]^+^ calcd. for C_26_H_36_NaO_6_ 467.2405; found 467.2404.

#### 3.3.8. Methyl-(2*E*)-2-[(2*R*,4a*R*,8*R*,8a*R*)-8-(methoxymethoxy)-4a,8-dimethyl-decahydronaphthalen-2-yl]-3-{3-methyl-2-oxo-2*H*,3*H*-[1,3oxazolo[4,5-b]pyridine-6-yl]}prop-2-enoate (**4i**)

White solid, m.p. 152–154 °C. [α]${}_{D}^{20}$ +92.7 (*c* 1.0, CH_2_Cl_2_). ^1^H-NMR (400 MHz, CDCl_3_): *δ* = 8.07 (s, 1H, H_pyr_), 7.48 (s, 1H, H-13), 7.35 (s, 1H, H_pyr_), 4.67 (dd, *J* = 7.5, 3.6Hz, 2H, OCH_2_), 3.82 (s, 3H, OCH_3_), 3.50 (s, 3H, CH_3_), 3.29 (s, 3H, OCH_3_), 2.73 (dddd, *J* = 12.3, 12.0, 4.0, 3.4 Hz, 1H, H-7), 2.23 (qd, *J* = 12.9, 3.4 Hz, 1H), 1.95 (dd, *J* = 12.6, 12.5 Hz, 1H), 1.85 (bd, *J* = 11.1 Hz, 1H), 1.74–1.63 (m, 2H), 1.59–1.51 (m, 1H), 1.51–1.22 (m, 5H), 1.21–1.11 (m, 4H), 1.10–0.99 (m, 4H) ppm. ^13^C-NMR (101 MHz, CDCl_3_): *δ* = 167.8 (C-12), 153.6 (CO), 145.5 (C_pyr_), 144.0 (CH_pyr_), 139.8 (C_pyr_), 137.1 (C-11), 134.1 (C-13), 127.2 (C_pyr_), 116.2 (CH_pyr_), 90.2 (OCH_2_), 78.2 (C-4), 55.2 (OCH_3_), 53.0 (OCH_3_), 51.9 (C-5), 44.8 (CH_2_), 40.8 (CH_2_), 39.5 (C-7), 39.4 (CH_2_), 34.7 (C-10), 27.2 (CH_3_), 25.9 (CH_2_), 25.5 (CH_2_), 19.8 (CH_2_), 19.6 (C-15), 19.3 (C-14) ppm. HRMS (ESI): *m*/*z* [M + H]^+^ calcd. for C_25_H_35_N_2_O_6_ 459.2488; found 459.2489.

### 3.4. General Procedure for Synthesis of Compounds **5a**–***5i***

To a solution of the previous substrates **4** (1 equiv) in diethyl ether (10 mL) HCl solution in ether (2 equiv) was added. The reaction mixture was stirred for 30 min at room temperature then water (10 mL) was added and mixture was extracted with diethyl ether (3 × 10 mL). The organic layers were dried (MgSO_4_), filtered and concentrated under reduce pressure leading to the desired products after purification by flash chromatography on silica gel (petroleum ether/EtOAc: 80/20).

#### 3.4.1. Methyl-(2*E*)-2-[(2*R*,4a*R*,8*R*,8a*R*)-8-hydroxy-4a,8-dimethyl-decahydronaphthalen-2-yl]-3-phenylprop-2-enoate (**5a**)

Colorless oil, [α]${}_{D}^{20}$ −33.2 (*c* 1.0, CH_2_Cl_2_). ^1^H-NMR (400 MHz, CDCl_3_): *δ* = 7.60 (s, 1H, H-13), 7.42–7.34 (m, 2H, H_Ar_), 7.34–7.24 (m, 3H, H_Ar_), 3.81 (s, 3H, OCH_3_), 2.85 (dddd, *J* = 12.4, 12.0, 3.7, 3.4 Hz, 1H, H-7), 2.28–2.15 (m, 1H), 1.98 (dd, *J* = 12.5, 12.5 Hz, 1H), 1.81–1.72 m, 1H), 1.69–1.61 (m, 1H), 1.61–1.47 (m, 3H), 1.46–1.28 (m, 5H), 1.20–1.13 (m, 2H), 1.12 (s, 3H, H-15), 1.00 (s, 3H, H-14) ppm. ^13^C-NMR (101 MHz, CDCl_3_): *δ* = 168.4 (C-12), 138.9 (C-13), 137.8 (C-11), 136.1 (C_Ar_), 128.9 (2CH_Ar_), 128.6 (2CH_Ar_), 128.2 (CH_Ar_), 72.4 (C-4), 55.0 (OCH_3_), 51.7 (C-5), 44.6 (CH_2_), 43.5 (CH_2_), 41.1 (CH_2_), 39.1 (C-7), 34.7 (C-10), 26.1 (CH_2_), 25.2 (CH_2_), 23.0 (C-15), 20.3 (CH_2_), 18.9 (C-14) ppm. HRMS (ESI): *m/z* [M + Na]^+^ calcd. for C_22_H_30_NaO_3_ 365.2087; found 365.2087.

#### 3.4.2. Methyl-(2*E*)-2-[(2*R*,4a*R*,8*R*,8a*R*)-8-hydroxy-4a,8-dimethyl-decahydronaphthalen-2-yl]-3-(4-methylphenyl)prop-2-enoate (**5b**)

Yellow oil, [α]${}_{D}^{20}$ +43.0 (*c* 1.0, CH_2_Cl_2_). ^1^H-NMR (250 MHz, CDCl_3_): *δ* = 7.57 (s, 1H, H-13), 7.18 (s, 4H, H_Ar_), 3.80 (s, 3H, OCH_3_), 2.88 (dddd, *J* = 12.2, 12.1, 4.0, 3.9 Hz, 1H, H-7), 2.37 (s, 3H), 2.32–2.12 (m, 1H), 1.98 (dd, *J* = 12.5, 12.4 Hz, 1H), 1.81–1.71 (m, 1H), 1.70–1.60 (m, 1H), 1.60–1.48 (m, 2H), 1.48–1.29 (m, 4H), 1.28–1.13 (m, 3H), 1.14–1.04 (s, 4H), 1.00 (s, 3H, H-14) ppm. ^13^C-NMR (63 MHz, CDCl_3_): *δ* = 168.5 (C-12), 139.0 (C_Ar_), 138.2 (C-13), 137.1 (C-11), 133.1 (C_Ar_), 129.3 (2CH_Ar_), 129.0 (2CH_Ar_), 72.3 (C-4), 55.1 (OCH_3_), 51.6 (C-5), 44.6 (CH_2_), 43.4 (CH_2_), 41.1 (CH_2_), 39.1 (C-7), 34.7 (C-10), 26.0 (CH_2_), 25.2 (CH_2_), 23.0 (C-15), 21.4 (CH_3_), 20.3 (CH_2_), 18.9 (C-14) ppm. HRMS (ESI): *m*/*z* [M + Na]^+^ calcd. for C_23_H_32_NaO_3_ 379.2242; found 379.2243.

#### 3.4.3. Methyl-(2*E*)-2-[(2*R*,4a*R*,8*R,*8a*R*)-8-hydroxy-4a,8-dimethyl-decahydronaphthalen-2-yl]-3-(4-methoxyphenyl)prop-2-enoate (**5c**)

White solid, m.p. 112–114 °C. [α]${}_{D}^{20}$ +73.0 (*c* 1.0, CH_2_Cl_2_); ^1^H-NMR (400 MHz, CDCl_3_): *δ* = 7.54 (s, 1H, H-13), 7.24 (d, *J* = 8.4 Hz, 2H, H_Ar_), 6.91 (d, *J* = 8.4 Hz, 2H, H_Ar_), 3.83 (s, 3H, OCH_3_), 3.79 (s, 3H, OCH_3_), 2.88 (dddd, *J* = 12.2, 12.0, 3.8, 3.8 Hz, 1H, H-7), 2.30–2.14 (m, 3H), 1.99 (dd, *J* = 12.6, 12.5 Hz, 1H), 1.81–1.72 (m, 1H), 1.70–1.61 (m, 1H), 1.57–1.48 (m, 2H), 1.46–1.23 (m, 4H), 1.23–1.14 (m, 2H), 1.12 (s, 3H, H-15), 1.00 (s, 3H, H-14) ppm. ^13^C-NMR (101 MHz, CDCl_3_): *δ* = 168.6 (C-12), 159.7 (C_Ar_), 138.7 (C-13), 136.1 (C-11), 130.6 (2CH_Ar_), 128.4 (C_Ar_), 114.1 (2CH_Ar_), 72.3 (C-4), 55.4 (OCH_3_), 55.1 (OCH_3_), 51.6 (C-5), 44.6 (CH_2_), 43.4 (CH_2_), 41.1 (CH_2_), 39.1 (C-7), 34.7 (C-10), 26.0 (CH_2_), 25.2 (CH_2_), 23.0 (C-15), 20.3 (CH_2_), 18.9 (C-14) ppm. HRMS (ESI): *m*/*z* [M + Na]^+^ calcd. for C_23_H_32_NaO_4_ 395.2191; found 395.2192.

#### 3.4.4. Methyl-(2*E*)-2-[(2*R*,4a*R*,8*R*,8a*R*)-8-hydroxy-4a,8-dimethyl-decahydronaphthalen-2-yl]-3-(4-formylphenyl)prop-2-enoate (**5d**)

White solid, m.p. 132–134°C. [α]${}_{D}^{20}$ −29.0 (*c* 1.0, CH_2_Cl_2_). ^1^H-NMR (400 MHz, CDCl_3_): *δ* = 10.02 (s, 1H, CHO), 7.89 (d, *J* = 8.1 Hz, 2H, H_Ar_), 7.58 (s, 1H, H-13), 7.42 (d, *J* = 7.9 Hz, 2H, H_Ar_), 3.82 (s, 3H, OCH_3_), 2.75 (dddd, *J* = 12.1, 12.1, 3.8, 3.7 Hz, 1H, H-7), 2.21 (qd, *J* = 13.8, 4.5 Hz, 1H), 1.96 (dd, *J* = 12.5, 12.3 Hz, 1H), 1.82–1.72 (m, 1H), 1.71–1.63 (m, 1H), 1.62–1.48 (m, 2H), 1.47–1.27 (m, 5H), 1.27–1.19 (m, 1H), 1.18–1.03 (m, 5H), 0.99 (s, 3H, H-14) ppm. ^13^C-NMR (101 MHz, CDCl_3_): *δ* = 191.8 (CHO), 167.9 (C-12), 142.4 (C_Ar_), 140.1 (C_Ar_), 137.3 (C-13), 135.7 (C-11), 130.0 (2CH_Ar_), 129.5 (2CH_Ar_), 72.3 (C-4), 55.0 (OCH_3_), 51.9 (C-5), 44.4 (CH_2_), 43.5 (CH_2_), 41.0 (CH_2_), 39.6 (C-7), 34.7 (C-10), 26.1 (CH_2_), 25.2 (CH_2_), 23.0 (C-15), 20.2 (CH_2_), 18.9 (C-14) ppm. HRMS (ESI): *m*/*z* [M + Na]^+^ calcd. for C_23_H_30_NaO_4_ 393.2039; found 393.2036.

#### 3.4.5. Methyl-(2*E*)-2-[(2*R*,4a*R*,8*R*,8a*R*)-8-hydroxy-4a,8-dimethyl-decahydronaphthalen-2-yl]-3-(4-fluorophenyl)prop-2-enoate (**5e**)

White solid, m.p. 101–103 °C, [α]${}_{D}^{20}$ −68.4 (*c* 1.0, CH_2_Cl_2_). ^1^H-NMR (400 MHz, CDCl_3_): *δ* = 7.54 (s, 1H, H-13), 7.33–7.20 (m, 2H, H_Ar_), 7.07 (dd, *J* = 8.5, 8.4 Hz, 2H, H_Ar_), 3.80 (s, 3H, OCH_3_), 2.79 (dddd, *J* = 12.2, 12.1, 4.0, 3.8 Hz, 1H, H-7), 2.21 (qd, *J* = 13.8, 12.8, 4.3 Hz, 1H), 1.97 (dd, *J* = 12.6, 12.3Hz, 1H), 1.80–1.73 (m, 1H), 1.69–1.61 (m, 1H), 1.60–1.47 (m, 4H), 1.46–1.29 (m, 5H), 1.21–1.13 (m, 1H), 1.12 (s, 3H, H-15), 0.99 (s, 3H, H-14) ppm. ^13^C-NMR (101 MHz, CDCl_3_): *δ* = 168.3 (C-12), 162.5 (d, *J* = 248.3 Hz, C_Ar_), 137.9 (C-13), 137.8 (C-11), 132.1 (d, *J* = 3.4 Hz, C_Ar_), 130.8 (d, *J* = 8.2 Hz, 2CH_Ar_), 115.7 (d, *J* = 21.7 Hz, 2CH_Ar_), 72.3 (C-4), 55.0 (OCH_3_), 51.7 (C-5), 44.6 (CH_2_), 43.5 (CH_2_), 41.1 (CH_2_), 39.2 (C-7), 34.7 (C-10), 26.0 (CH_2_), 25.2 (CH_2_), 23.0 (C-15), 20.3 (CH_2_), 18.9 (C-14) ppm. HRMS (ESI): *m*/*z* [M + Na]^+^ calcd. for C_22_H_29_FNaO_3_ 383.1992; found 383.1992.

#### 3.4.6. Methyl-(2*E*)-2-[(2*R*,4a*R*,8*R*,8a*R*)-8-hydroxy-4a,8-dimethyl-decahydronaphthalen-2-yl]-3-(3-methoxyphenyl)prop-2-enoate (**5f**)

Colorless oil, [α]${}_{D}^{20}$ +71.3.5 (*c* 1.0, CH_2_Cl_2_). ^1^H-NMR (400 MHz, CDCl_3_): *δ* = 7.56 (s, 1H, H-13), 7.32–7.24 (m, 1H, H_Ar_), 6.90–6.79 (m, 3H, H_Ar_), 3.81 (s, 3H, OCH_3_), 3.81 (s, 3H, OCH_3_), 2.86 (dddd, *J* = 12.3, 12.3, 3.9, 3.8 Hz, 1H, H-7), 2.28–2.14 (m, 1H), 1.97 (dd, *J* = 12.5, 12.5 Hz, 1H), 1.81–1.72 (m, 1H), 1.71–1.65 (m, 1H), 1.63–1.49 (m, 3H), 1.46–1.24 (m, 5H), 1.22–1.13 (m, 2H), 1.12 (s, 3H, H-15), 0.99 (s, 3H, H-14) ppm. ^13^C-NMR (101 MHz, CDCl_3_): *δ* = 168.4 (C-12), 159.7 (C_Ar_), 138.8 (C-13), 138.0 (C-11), 137.4 (C_Ar_), 129.6 (CH_Ar_), 121.3 (CH_Ar_), 114.2 (CH_Ar_), 114.0 (CH_Ar_), 72.3 (C-4), 55.4 (OCH_3_), 55.1 (OCH_3_), 51.7 (C-5), 44.6 (CH_2_), 43.5 (CH_2_), 41.1 (CH_2_), 39.3 (C-7), 34.7 (C-10), 26.1 (CH_2_), 25.3 (CH_2_), 23.0 (C-15), 20.3 (CH_2_), 18.9 (C-14) ppm. HRMS (ESI): *m*/*z* [M + Na]^+^ calcd. for C_23_H_32_NaO_4_ 395.2188; found 395.2192.

#### 3.4.7. Methyl-(2*E*)-2-[(2*R*,4a*R*,8*R*,8a*R*)-8-hydroxy-4a,8-dimethyl-decahydronaphthalen-2-yl]-3-(2-fluorophenyl)prop-2-enoate (**5g**)

Colorless oil, [α]${}_{D}^{20}$ −52.8 (*c* 1.0, CH_2_Cl_2_). ^1^H-NMR (400 MHz, CDCl_3_): *δ* = 7.57 (s, 1H, H-13), 7.30 (bdd, *J* = 6.9, 6.8 Hz, 1H, H_Ar_), 7.22 (dd, *J* = 7.4, 7.3 Hz, 1H, H_Ar_),7.14 (dd, *J* = 7.6, 7.4 Hz, 1H, H_Ar_), 7.08 (dd, *J* = 9.2, 9.1 Hz, 1H, H_Ar_),3.81 (s, 3H, OCH_3_), 2.69 (dddd, *J* = 12.2, 12.2, 3.9, 3.6 Hz, 1H, H-7), 2.20 (qd, *J* = 13.5, 12.8, 4.3 Hz, 1H), 1.97 (dd, *J* = 12.5, 12.5 Hz, 1H), 1.81–1.71 (m, 1H), 1.69–1.59(m, 1H), 1.45–1.28 (m, 2H), 1.45–1.20 (m, 5H), 1.19–1.12 (m, 2H), 1.11 (s, 3H, H-15), 1.10–1.00 (m, 1H), 0.98 (s, 3H, H-14) ppm. ^13^C-NMR (101 MHz, CDCl_3_): *δ* = 167.8 (C-12), 160.3 (d, *J* = 248.6 Hz, C_Ar_), 139.7 (C-11), 131.9 (d, *J* = 3.2 Hz, C-13), 130.2 (d, *J* = 1.9 Hz, CH_Ar_), 130.1 (d, *J* = 3.3 Hz, CH_Ar_), 124.1 (d, *J* = 3.6 Hz, CH_Ar_), 123.9 (d, *J* = 14.7 Hz, C_Ar_), 115.8 (d, *J* = 21.9 Hz, CH_Ar_), 72.3 (C-4), 55.0 (OCH_3_), 51.8 (C-5), 44.6 (CH_2_), 43.4 (CH_2_), 41.1 (CH_2_), 39.9 (C-7), 34.7 (C-10), 25.8 (CH_2_), 25.1 (CH_2_), 23.1 (C-15), 20.2 (CH_2_), 18.9 (C-14) ppm. HRMS (ESI): *m/z* [M + Na]^+^ calcd. for C_22_H_29_FNaO_3_ 383.1992; found 383.1992.

#### 3.4.8. Methyl-2-[(1*E*)-2-[(2*R*,4a*R*,8*R*,8a*R*)-8-hydroxy-4a,8-dimethyl-decahydronaphthalen-2-yl]-3-methoxy-3-oxoprop-1-en-1-yl]benzoate (**5h**)

White solid, m.p. 115–117 °C. [α]${}_{D}^{20}$ +82.1 (*c* 1.0, CH_2_Cl_2_). ^1^H-NMR (400 MHz, CDCl_3_): *δ* = 8.03 (d, *J* = 7.9 Hz, 1H, H_Ar_), 8.00 (s, 1H, H-13), 7.53 (dd, *J* = 7.7, 7.4 Hz, 1H, H_Ar_), 7.40 (dd, *J* = 7.7, 7.4 Hz, 1H, H_Ar_), 7.22 (d, *J* = 7.7 Hz, 1H, H_Ar_), 3.88 (s, 3H, OCH_3_), 3.82 (s, 3H, OCH_3_), 2.46 (dddd, *J* = 12.3, 12.1, 4.2, 4.2 Hz, 1H, H-7), 2.15 (qd, *J* = 13.4, 12.7, 4.1 Hz, 1H), 1.92 (dd, *J* = 12.5, 12.4Hz, 1H), 1.78–1.69 (m, 1H), 1.62–1.45 (m, 6H), 1.40–1.28 (m, 5H), 1.21–1.13 (m, 1H), 1.09 (s, 3H, H-15), 1.07–0.97 (m, 2H), 0.95 (s, 3H, H-14) ppm. ^13^C-NMR (101 MHz, CDCl_3_): *δ* = 168.1 (C-12), 167.0 (CO_2_Me), 140.3 (C-13), 138.4 (C-11), 136.1 (C_Ar_), 132.3 (CH_Ar_), 130.9 (CH_Ar_), 129.6 (CH_Ar_), 129.1 (C_Ar_), 128.0 (CH_Ar_), 72.3 (C-4), 55.0 (OCH_3_), 52.3 (OCH_3_), 51.8 (C-5), 44.5 (CH_2_), 43.4 (CH_2_), 41.1 (CH_2_), 39.7 (C-7), 34.6 (C-10), 26.1 (CH_2_), 25.2 (CH_2_), 23.0 (C-15), 20.2 (CH_2_), 18.8 (C-14) ppm. HRMS (ESI): *m/z* [M + Na]^+^ calcd. for C_24_H_32_NaO_5_ 423.2142; found 423.2141.

#### 3.4.9. Methyl-(2*E*)-2-[(2*R*,4a*R*,8*R*,8a*R*)-8-hydroxy-4a,8-dimethyl-decahydronaphthalen-2-yl]-3-{3-methyl-2-oxo-2*H*,3*H*-[1,3oxazolo[4,5-b]pyridine-6-yl]}prop-2-enoate (**5i**)

Colorless oil, [α]${}_{D}^{20}$ +87.4 (*c* 1.0, CH_2_Cl_2_). ^1^H-NMR (400 MHz, CDCl_3_): *δ* = 8.07 (s, 1H, H_pyr_), 7.49 (s, 1H, H-13), 7.32 (s, 1H, H_pyr_), 3.82 (s, 3H, OCH_3_), 3.50 (s, 3H), 2.74 (dddd, *J* = 12.3, 12.0, 4.2, 4.0 Hz, 1H, H-7), 2.31–2.18 (m, 1H), 1.98 (dd, *J* = 12.5, 12.3 Hz, 1H), 1.81–1.73 (m, 1H), 1.71–1.61 (m, 2H), 1.59–1.49 (m, 1H), 1.48–1.29 (m, 5H), 1.22–1.13 (m, 2H), 1.12 (s, 3H, H-15), 1.00 (s, 3H, H-14), 0.90 (dq, *J* = 12.5, 7.4 Hz, 1H) ppm. ^13^C-NMR (101 MHz, CDCl_3_): *δ* = 167.7 (C-12), 153.6 (CO), 145.6 (C_pyr_), 143.7 (CH_pyr_), 139.7 (C_pyr_), 137.1 (C-11), 134.2 (C-13), 127.1 (C_pyr_), 116.2 (CH_pyr_), 72.3 (C-4), 55.1 (OCH_3_), 51.9 (C-5), 44.4 (CH_2_), 43.6 (CH_2_), 41.0 (CH_2_), 39.5 (C-7), 34.7 (C-10), 27.2 (CH_3_), 26.0 (CH_2_), 25.0 (CH_2_), 22.9 (C-15), 20.3 (CH_2_), 18.9 (C-14) ppm. HRMS (ESI): *m*/*z* [M + H]^+^ calcd. for C_23_H_31_N_2_O_5_ 415.2217; found 415.2227.

### 3.5. Synthesis of Compounds ***6*** and ***7***

#### 3.5.1. Methyl-2-[(2*R*,4a*R*,8a*R*)-4a,8-dimethyl-1,2,3,4,4*a*,5,6,8*a*-octahydronaphthalen-2-yl]acrylate (**6**) [37]

Compound **1** (200 mg, 0.85 mmol) was dissolved in a mixture of toluene–methanol (8:2, 10 mL). The solution was cooled to 0 °C then TMSCHN_2_ (0.5 mL, 2M in diethyl-ether) was added. After completion, the reaction mixture was concentrated under reduced pressure and the residue was purified by flash chromatography on silica gel (petroleum ether/ethyl acetate: 98/2) provided **6** (158 mg, 75%) as a colorless oil; [α]${}_{D}^{20}$ +26.8 (*c* 1.0, CH_2_Cl_2_); ^1^H-NMR (250.13 MHz, CDCl_3_) *δ* = 0.82 (s, 3H, H-14), 1.16 (dd, 1H, *J* = 12.4, 12.4 Hz), 1.29–1.41 (m, 3H), 1.44 (dd, 1H, *J* = 3.4, 2.9 Hz), 1.47–1.53 (m, 1H), 1.54–1.69 (m, 4H), 1.75–1.88 (m, 1H), 1.90–2.14 (m, 3H), 2.52 (ddd, 1H, *J* = 11.9, 4.3, 4.0 Hz), 3.76 (s, 3H, OMe), 5.31 (bs, 1H, H-3), 5.56 (bs, 1H, H-13), 6.14 (bs, 1H, H-13); ^13^C-NMR (62.9 MHz, CDCl_3_) *δ* = 15.8 (C-15), 21.3 (C-14), 23.1 (C-2), 27.6 (C-8), 29.5 (C-6), 32.4 (C-10), 38.0 (C-1), 40.3 (C-9), 40.7 (C-7), 47.0 (C-5), 51.9 (OMe), 121.2 (C-3), 122.7 (C-13), 135.0 (C-4), 146.1 (C-11), 168.1 (C-12).

#### 3.5.2. Methyl-2-[(1a*R*,3a*S*,6*R*,7a*R*,7b*S*)-3a,7b-dimethyldecahydronaphtho[1,2-b]oxiren-6-yl]acrylate (**7**) [38]

To a solution of ester **6** (315 mg, 1.3 mmol) in 10 mL of dichloromethane were added (220 mg, 1.3 mmol) of *m*-chloroperbenzoic acid. The reaction mixture was stirred at room temperature for 3 h then washed with a solution of sodium bisulfite (10%) (3 × 10 mL) then a solution of sodium hydrogen carbonate (5%) (10 mL). The aqueous layers were combined and extracted with DCM (3 × 10 mL). The organic layers were combined, washed with water (10 mL), dried with MgSO_4_, filtered and concentrated under reduced pressure. The resulting residue was purified by flash chromatography on silica gel. (petroleum ether/ethyl acetate:9.7/0.3) provided **7** (236 mg, 70%) as a colorless oil; [α]${}_{D}^{20}$ +10.7 (*c* 1.0, CH_2_Cl_2_); ^1^H-NMR (250.13 MHz, CDCl_3_) *δ* = 0.83 (s, 3H, H-14), 1.03–1.22 (m, 7H), 1.33 (ddd, 1H, *J* = 13.4, 3.5, 3.3 Hz), 1.40–1.48 (m, 1H), 1.48-1.53 (m, 1H), 1.57 (dd, 1H, *J* = 13.4, 3.3 Hz), 1.77–1.84 (m, 1H), 1.87 (ddd, 1H, *J* = 12.1, 6.7, 3.1 Hz), 1.94 (dd, 1H, *J* = 15.1, 6.1 Hz), 2.43 (dddd, 1H, *J* = 12.1, 12.0, 3.9, 3.9 Hz), 2.86 (bs, 1H, H-3), 3.70 (s, 3H, CO_2_Me), 5.50 (s, 1H, H-13), 6.09 (s, 1H, H-13); ^13^C-NMR (62.9 MHz, CDCl_3_) *δ* = 16.5 (C-14), 21.3 (C-15), 21.7 (CH_2_), 27.3 (CH_2_), 30.1 (CH_2_), 31.6 (C-10), 34.8 (CH_2_), 39.8 (CH_2_), 40.7 (C-7), 48.2 (C-5), 52.1 (OMe), 58.8 (C-4), 61.1 (C-3), 123.1 (C-13), 145.5 (C-11), 168.0 (C-12). HRMS (ESI): calcd. For C_16_H_25_O_3_ [M + H]^+^ 265.1800; found 265.1798.

### 3.6. Synthesis of Compounds ***8a–8e***, ***9a*** and ***9b***

A solution of substrate **3** (1 equiv), Pd(OAc)_2_, (0.1 equiv) and the appropriate haloaryl compound (1.1 equiv) in the presence of triethylamine (3 eqiv) and P(*o*-Tol)_3_ (0.1 equiv) in DMF (2 mL) was stirred for 24 h at 120 °C. After cooling, water (10 mL) was added to the reaction mixture which was then extracted with EtOAc (3 × 10 mL). The combined organic layers were washed with water (2 × 30 mL), dried (MgSO_4_), filtered and concentrated under reduce pressure. The expected compounds were obtained after purification by flash chromatography on silica gel (petroleum ether/EtOAc:80/20).

#### 3.6.1. Methyl-2-[(2*R*,4a*S*,7*R*,8a*R*)-7-hydroxy-4a-methyl-8-methylidene-decahydronaphthalen-2-yl]-3-phenylprop-2-enoate (**8a**)

Yellow oil, [α]${}_{D}^{20}$ −15.4 (*c* 1.0, CH_2_Cl_2_). ^1^H-NMR (400 MHz, CDCl_3_): *δ* = 7.61 (s, 1H, H-13), 7.37 (dd, *J* = 8.3, 6.9 Hz, 2H, H_Ar_), 7.30 (dd, *J* = 7.3, 7.2 Hz, 1H, H_Ar_), 7.24 bs, 2H, H_Ar_), 4.91 (d, *J* = 1.7 Hz, 1H, H-15), 4.57 (s, 1H, H-15), 4.27 (s, 1H, H-3), 3.80 (s, 3H, OCH_3_), 2.92 (dddd, *J* = 12.3, 12.0, 3.7, 3.4 Hz, 1H, H-7), 2.29–2.14 (m, 2H), 2.06 (dd, *J* = 12.5, 12.4 Hz, 1H), 1.91–1.56 (m, 3H), 1.54–1.12 (m, 6H), 0.80 (s, 3H, H-14) ppm. ^13^C-NMR (101 MHz, CDCl_3_): *δ* = 168.4 (C-12), 151.7 (C-4), 139.1 (C-13), 137.8 (C-11), 136.0 (C_Ar_), 128.9 (2CH_Ar_), 128.6 (2CH_Ar_), 128.2 (CH_Ar_), 109.4 (C-15), 73.8 (C-3), 51.7 (OCH_3_), 43.7 (C-5), 40.7 (CH_2_), 38.5 (C-7), 35.9 (C-10), 35.6 (CH_2_), 29.9 (CH_2_), 28.1 (CH_2_), 25.8 (CH_2_), 15.7 (C-14) ppm. HRMS (ESI): *m/z* [M + H]^+^ calcd. for C_22_H_29_O_3_ 341.2111; found 341.2111.

#### 3.6.2. Methyl-2-[(2*R*,4a*S*,7*R*,8a*R*)-7-hydroxy-4a-methyl-8-methylidene-decahydronaphthalen-2-yl]-3-(4-methylphenyl)prop-2-enoate (**8b**)

Yellowish oil, [α]${}_{D}^{20}$ +63.1 (*c* 1.0, CH_2_Cl_2_). ^1^H-NMR (250 MHz, CDCl_3_): *δ* = 7.60 (s, 1H, H-13), 7.18 (s, 4H, H_Ar_), 4.93 (dd, *J* = 1.6, 1.6 Hz, 1H, H-15), 4.59 (dd, *J* = 1.8, 1.8 Hz, 1H, H-15), 4.28 (dd, *J* = 2.8, 2.8 Hz, 1H, H-3), 3.81 (s, 3H, OCH_3_), 2.96 (dddd, *J* = 12.3, 12.2, 4.1, 3.9 Hz, 1H, H-7), 2.37 (s, 3H), 2.32–2.19 (m, 1H), 2.08 (dd, *J* = 12.5, 12.4 Hz, 1H), 1.87–1.64 (m, 3H), 1.55–1.31 (m, 5H), 1.30–1.09 (m, 2H),0.82 (s, 3H, H-14) ppm. ^13^C-NMR (63 MHz, CDCl_3_): *δ* = 168.5 (C-12), 151.7 (C-4), 139.2 (C-13), 138.3 (C_Ar_), 137.1 (C-11), 133.1 (C_Ar_), 129.3 (2CH_Ar_), 128.9 (2CH_Ar_), 109.5 (C-15), 73.8 (C-3), 51.6 (OCH_3_), 43.8 (C-5), 40.8 (CH_2_), 38.5 (C-7), 35.9 (C-10), 35.6 (CH_2_), 29.9 (CH_2_), 28.1 (CH_2_), 25.8 (CH_2_), 21.4 (CH_3_), 15.8 (C-14) ppm. HRMS (ESI): *m*/*z* [M + H]^+^ calcd. for C_23_H_31_O_3_ 355.2266; found 355.2267.

#### 3.6.3. Methyl-2-[(2*R*,4a*S*,7*R*,8a*R*)-7-hydroxy-4a-methyl-8-methylidene-decahydronaphthalen-2-yl]-3-(4-methoxyphenyl)prop-2-enoate (**8c**)

Yellow solid, m.p. 107–109 °C. [α]${}_{D}^{20}$ +114.6 (*c* 1.0, CH_2_Cl_2_). ^1^H-NMR (250 MHz, CDCl_3_): *δ* = 7.57 (s, 1H, H-13), 7.23 (d, *J* = 8.7 Hz, 2H, H_Ar_), 6.91 (d, *J* = 8.7 Hz, 2H, H_Ar_), 4.93 (dd, *J* = 1.6, 1.6 Hz, 1H, H-15), 4.59 (dd, *J* = 1.8, 1.8 Hz, 1H, H-15), 4.29 (dd, *J* = 2.8, 2.8 Hz, 1H, H-3), 3.83 (s, 3H, OCH_3_), 3.80 (s, 3H, OCH_3_), 2.98 (dddd, *J* = 12.2, 12.2, 3.9, 3.8 Hz, 1H, H-7), 2.35–2.20 (m, 2H), 2.09 (dd, *J* = 12.3, 12.2 Hz, 1H), 1.95–1.59 (m, 4H), 1.56–1.28 (m, 5H), 0.82 (s, 3H, H-14) ppm. ^13^C-NMR (63 MHz, CDCl_3_): *δ* = 168.6 (C-12), 159.7 (C_Ar_), 151.7 (C-4), 138.9 (C-13), 136.1 (C-11), 130.6 (2CH_Ar_), 128.4 (C_Ar_), 114.1 (2CH_Ar_), 109.5 (C-15), 73.8 (C-3), 55.4 (OCH_3_), 51.6 (OCH_3_), 43.8 (C-5), 40.8 (CH_2_), 38.4 (C-7), 35.9 (C-10), 35.6 (CH_2_), 29.9 (CH_2_), 28.1 (CH_2_), 25.8 (CH_2_), 15.8 (C-14) ppm. HRMS (ESI): *m/z* [M + H]^+^ calcd. for C_23_H_31_O_4_ 371.2215; found 371.2216.

#### 3.6.4. Methyl-2-[(2*R*,4a*S*,7*R*,8a*R*)-7-hydroxy-4a-methyl-8-methylidene-decahydronaphthalen-2-yl]-3-(3-methoxyphenyl)prop-2-enoate (**8d**)

Colorless oil, [α]${}_{D}^{20}$ +91.0 (*c* 1.0, CH_2_Cl_2_). ^1^H-NMR (250 MHz, CDCl_3_): *δ* = 7.59 (s, 1H, H-13), 7.33–7.24 (m, 1H, H_Ar_), 6.89–6.77 (m, 3H, H_Ar_), 4.94–4.90 (m, 1H, H-15), 4.57 (dt, *J* = 3.5, 1.7 Hz, 1H, H-15), 4.28 (t, *J* = 2.6 Hz, 1H, H-3), 3.81 (d, *J* = 0.6 Hz, 6H, 2OCH_3_), 2.94 (tt, *J* = 12.1, 3.9 Hz, 1H, H-7), 2.33–2.16 (m, 2H), 2.06 (q, *J* = 12.3 Hz, 1H), 1.89–1.12 (m, 9H), 0.81 (s, 3H, H-14) ppm. ^13^C-NMR (63 MHz, CDCl_3_): *δ* = 168.6 (C-12), 159.8 (C_Ar_), 151.9 (C-4), 139.2 (C-13), 138.2 (C-11), 137.5 (C_Ar_), 129.8 (CH_Ar_), 121.3 (CH_Ar_), 114.5 (CH_Ar_), 114.0 (CH_Ar_), 109.6 (C-15), 73.9 (C-3), 55.5 (OCH_3_), 51.9 (OCH_3_), 43.9 (C-5), 40.9 (CH_2_), 38.8 (C-7), 36.1 (C-10), 35.8 (CH_2_), 30.0 (CH_2_), 28.3 (CH_2_), 26.0 (CH_2_), 15.9 (C-14) ppm. HRMS (ESI): *m/z* [M + H]^+^ calcd. for C_23_H_31_O_4_ 371.2216; found 371.2216.

#### 3.6.5. Methyl-2-[(2*R*,4a*S*,7*R*,8a*R*)-7-hydroxy-4a-methyl-8-methylidene-decahydronaphthalen-2-yl]-3-(2-methylphenyl)prop-2-enoate (**8e**)

Colorless oil, [α]${}_{D}^{20}$ +42.3 (*c* 1.0, CHCl_3_). ^1^H-NMR (250 MHz, CDCl_3_): *δ* = 7.65 (s, 1H, H-13), 7.25–7.01 (s, 4H, H_Ar_), 4.91 (dd, *J* = 1.6, 1.6 Hz, 1H, H-15), 4.57 (dd, *J* = 1.8, 1.8 Hz, 1H, H-15), 4.26 (dd, *J* = 2.3, 2.3 Hz, 1H, H-3), 3.82 (s, 3H, OCH_3_), 2.69 (dddd, *J* = 12.3, 12.1, 4.1, 3.8 Hz, 1H, H-7), 2.26 (s, 3H), 2.23–2.13 (m, 2H), 2.03 (q, *J* = 12.2, 12.4 Hz, 1H), 1.85–1.53 (m, 3H), 1.51–1.10 (m, 6H), 0.78 (s, 3H, H-14) ppm. ^13^C-NMR (63 MHz, CDCl_3_): *δ* = 168.3 (C-12), 151.7 (C-4), 139.2 (C-13), 138.0 (C_Ar_), 136.4 (C-11), 135.6 (C_Ar_), 130.1 (CH_Ar_), 128.2 (CH_Ar_), 128.1 (CH_Ar_), 125.8 (CH_Ar_), 109.4 (C-15), 73.8 (C-3), 51.7 (OCH_3_), 43.6 (C-5), 40.7 (CH_2_), 38.8 (C-7), 35.8 (C-10), 35.6 (CH_2_), 29.9 (CH_2_), 28.1 (CH_2_), 25.9 (CH_2_), 20.2 (CH_3_), 15.7 (C-14). HRMS (ESI): *m*/*z* [M + H]^+^ calcd. for C_23_H_31_O_3_ 355.2268; found 355.2269.

#### 3.6.6. Methyl-2-[(2*R*,4a*S*,7*R*,8a*R*)-7-hydroxy-4a-methyl-8-methylidene-decahydronaphthalen-2-yl]prop-2-enoate (**9a**) [6]

Colorless oil, [α]${}_{D}^{20}$ +35.3 (*c* 1.0, CH_2_Cl_2_). ^1^H-NMR (250 MHz, CDCl_3_): *δ* = 6.15 (d, *J* = 1.1 Hz, 1H, H-13), 5.56 (dd, *J* = 1.1, 1.1 Hz, 1H, H-13), 4.93 (dd, *J* = 1.1, 1.1 Hz, 1H, H-15), 4.55 (dd, *J* = 1.8, 1.7 Hz, 1H, H-15), 4.34–4.24 (m, 1H, H-3), 3.75 (s, 3H, OCH_3_), 2.68–2.50 (m, 1H, H-7), 2.44 (ddt, *J* = 12.2, 3.2, 1.8 Hz, 1H), 1.87–1.70 (m, 3H), 1.69–1.36 (m, 5H), 1.30–1.14 (m, 3H), 0.72 (s, 3H, H-14) ppm. ^13^C-NMR (63 MHz, CDCl_3_): *δ* = 168.0 (C-12), 151.8 (C-4), 145.9 (C-11), 122.7 (C-13), 109.2 (C-15), 73.6 (C-3), 51.9 (OCH_3_), 43.8 (C-5), 40.8 (CH_2_), 39.8 (C-7), 35.9 (C-10), 35.8 (CH_2_), 29.8 (CH_2_), 29.7 (CH_2_), 27.3 (CH_2_), 15.7 (C-14) ppm. HRMS (ESI): *m/z* [M + Li]^+^ calcd. for C_16_H_24_LiO_3_ 271.1881; found 271.1880.

#### 3.6.7. Methyl-2-[(2*R*,4a*S*,7*R*)-7-hydroxy-4a,8-dimethyl-1,2,3,4,4a,5,6,7-octahydronaphthalen-2-yl]prop-2-enoate (**9b**) [37]

Colorless oil, [α]${}_{D}^{20}$ −4.7 (*c* 1.0, CH_2_Cl_2_). ^1^H-NMR (250 MHz, CDCl_3_): *δ* = 6.18 (d, *J* = 0.7 Hz, 1H, H-13), 5.57 (dd, *J* = 1.1, 1.0 Hz, 1H, H-13), 3.89 (s, 1H, H-3), 3.76 (s, 3H, OCH_3_), 2.68–5.56 (m, 1H, H-7), 2.53–2.35 (m, 1H), 1.85–1.79 (m, 1H), 1.79–1.72 (m, 4H), 1.72–1.45 (m, 6H), 1.45–1.21 (m, 2H), 1.03 (s, 3H, H-14) ppm. ^13^C-NMR (63 MHz, CDCl_3_): *δ* = 167.8 (C-12), 145.5 (C-11), 139.7 (C-5), 126.5 (C-4), 122.9 (C-13), 69.9 (C-3), 51.9 (OCH_3_), 42.3 (CH_2_), 40.5 (C-7), 35.0 (C-10), 34.2 (CH_2_), 31.6 (CH_2_), 27.8 (CH_2_), 23.1 (C-15), 17.2 (C-14) ppm. HRMS (ESI): *m/z* [M + Na]^+^ calcd. for C_16_H_24_NaO_3_ 287.1618; found 287.1617.

### 3.7. Synthesis of Methyl-2-[(2R,4aS,7R,8aR)-7-(methoxymethoxy)-4a-methyl-8-methylidenedecahydro-naphthalen-2-yl]prop-2-enoate (***10***)

To a solution of methyl-2-[(2*R*,4a*S*,7*R*,8a*R*)-7-hydroxy-4a-methyl-8-methylidenedecahydro-naphthalen-2-yl]prop-2-enoate (**9a**, 8 mg, 0.37 mmol, 1 equiv) in anhydrous CH_2_Cl_2_ (10 mL) were added DPA (0.26 mL, 1.51 mmol, 4 equiv) and MOMCl (0.12 mL, 1.51 mmol, 4 equiv). The reaction mixture was stirred for 12 h at room temperature under argon. Then water (10 mL) was added and the mixture was extracted with CH_2_Cl_2_ (3 × 10 mL). The organic layers were dried (MgSO_4_), filtered and concentrated under reduce pressure to give the desired product **10** (82 mg, 0.26 mmol, 70% yield) after purification by flash chromatography on silica gel (petroleum ether/EtOAc; 90:10). Colorless oil, [α]${}_{D}^{20}$ +28.8 (*c* 1.0, CH_2_Cl_2_). ^1^H-NMR (400 MHz, CDCl_3_): *δ* = 6.15 (d, *J* = 1.1 Hz, 1H, H-13), 5.57 (dd, *J* = 1.1, 1.0 Hz, 1H, H-13), 4.93 (bs, 1H, H-15), 4.67 (dd, *J* = 1.7, 1.6 Hz, 1H, H-15), 4.62 (d, *J* = 6.6 Hz, 1H, OCH_2_), 4.50 (d, *J* = 6.6 Hz, 1H, OCH_2_), 4.12 (dd, *J* = 3.0, 2.7Hz, 1H, H-3), 3.75 (s, 3H, OCH_3_), 3.35 (s, 3H, OCH_3_), 2.57 (dddd, *J* = 11.9, 11.9, 3.3, 2.7 Hz, 1H, H-7), 2.26 (bd, *J* = 12.3, 1H), 1.88–1.79 (m, 2H), 1.74–1.34 (m, 6H), 1.32–1.21 (m, 2H), 0.74 (s, 3H, H-14) ppm. ^13^C-NMR (101 MHz, CDCl_3_): *δ* = 168.0 (C-12), 148.4 (C-4), 146.0 (C-11), 122.7 (C-13), 111.0 (C-15), 93.1 (OCH_2_), 76.4 (C-3), 55.3 (OCH_3_), 51.9 (OCH_3_), 44.3 (C-5), 40.8 (CH_2_), 39.8 (C-7), 36.4 (C-10), 35.8 (CH_2_), 29.7 (CH_2_), 28.7 (CH_2_), 27.4 (CH_2_), 16.0 (C-14) ppm. HRMS (ESI): *m*/*z* [M + H]^+^ calcd. for C_18_H_29_O_4_ 309.2061; found 309.2060.

### 3.8. Synthesis of Methyl-2-[(2R,4aS,7R,8aR)-7-(methoxymethoxy)-4a-methyl-8-methylidenedecahydro-naphthalen-2-yl]-3-(4-methylphenyl)prop-2-enoate (***11***)

A solution of substrate **10** (180 mg, 0.58 mmol, 1 equiv), Pd(OAc)_2_, (13 mg, 0.06 mmol, 0.1 equiv) and 4-bromotoluene (110 mg, 0.64 mmol, 1.1 equiv) in the presence of triethylamine (0.24 mL, 1.75 mmol, 3 equiv) and P(*o*-Tol)_3_ (18 mg, 0.06 mmol, 0.1 equiv) in DMF (2 mL) was stirred for 24 h at 120 °C. After cooling, water (10 mL) was added to the reaction mixture which was extracted with EtOAc (3 × 10 mL). The combined organic layers were washed with water (2 × 30 mL), dried (MgSO_4_), filtered and concentrated under reduced pressure. The expected compound **11** (157 mg, 0.40 mmol) was obtained after purification by flash chromatography on silica gel (petroleum ether/EtOAc:90/10). Colorless oil, [α]${}_{D}^{20}$ +42.5 (*c* 1.0, CH_2_Cl_2_). ^1^H-NMR (400 MHz, CDCl_3_): *δ* = 7.59 (s, 1H, H-13), 7.18 (s, 4H, H_Ar_), 4.92 (bs, 1H, H-15), 4.69 (bs, 1H, H-15), 4.60 (d, *J* = 6.6 Hz, 1H, OCH_2_), 4.50 (d, *J* = 6.6 Hz, 1H, OCH_2_), 4.11 (dd, *J* = 2.8, 2.8 Hz, 1H, H-3), 3.80 (s, 3H, OCH_3_), 3.34 (s, 3H, OCH_3_), 3.00–2.89 (m, 1H, H-7), 2.37 (s, 3H), 2.26 (qd, *J* = 13.0, 3.7 Hz, 1H), 2.13–2.05 (m, 2H), 1.85–1.78 (m, 2H), 1.68–1.57 (m, 1H), 1.54–1.40 (m, 2H), 1.39–1.20 (m, 3H), 0.84 (s, 3H, H-14) ppm. ^13^C-NMR (101 MHz, CDCl_3_): *δ* = 168.4 (C-12), 148.4 (C-4), 139.0 (C-13), 138.2 (C_Ar_), 137.1 (C-11), 133.1 (C_Ar_), 129.3 (2CH_Ar_), 128.9 (2CH_Ar_), 111.0 (C-15), 93.2 (OCH_2_), 76.7 (C-3), 55.3 (OCH_3_), 51.6 (OCH_3_), 44.2 (C-5), 40.8 (CH_2_), 38.5 (C-7), 36.2 (C-10), 35.8 (CH_2_), 28.6 (CH_2_), 28.0 (CH_2_), 25.8 (CH_2_), 21.4 (CH_3_), 16.0 (C-14) ppm. HRMS (ESI): *m*/*z* [M + H]^+^ calcd. for C_25_H_35_O_4_ 399.2532; found 399.2529.

## 4. Conclusions

The ilicic and isocostic acids used in this study were extracted directly from *Dittrichia viscosa* L. Greuter. These readily accessible enantiopure compounds were submitted to the Mizoroki-Heck reaction after some minor modifications. Various aryl halides were introduced, with moderate to good yields, on the α,β-unsaturated methyl ester group with an *E* configuration. No racemization was observed and the mild experimental conditions could be used to synthesize a wide variety of compounds, starting from the plant extract, in a limited number of steps.

## Figures and Tables

**Table 1:** Optimization of the Mizoroki-Heck reaction of ilicic acid.

Entry	X	Pd(OAc)_2_ (equiv)	Additive (equiv)	Et_3_N (equiv)	Yield (%) ^a^
1	I	0.1	-	3	30
2	I	0.1	AgOAc (1)	-	20
3	I	0.1	P(*o*Tol)_3_ (0.4)	3	30
4	I	0.1	P(*o*Tol)_3_ (0.2)	3	60
5	I	0.1	P(*o*Tol)_3_ (0.1)	3	65 (45) ^b^
6	I	0.2	P(*o*Tol)_3_ (0.2)	3	58
7	I	0.1	P(*o*Tol)_3_ (0.1)	2	51
8	Br	0.1	P(*o*Tol)_3_ (0.1)	3	60

^a^ Isolated yields. ^b^ Reaction performed in MeCN.

**Table 2:** Scope of substrates and deprotection.

Entry	ArX	Product 4, Yield	Product 5, Yield
1	X = I	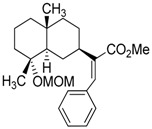 **4a**, 62%	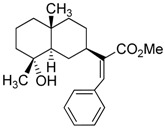 **5a**, 90%
2	X = I	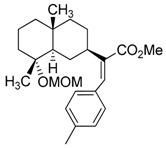 **4b**, 65%	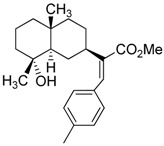 **5b**, 88%
3	X = I	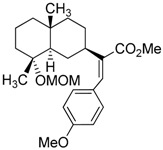 **4c**, 63%	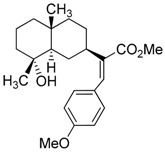 **5c**, 80%
4	X = Br	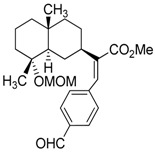 **4d**, 58%	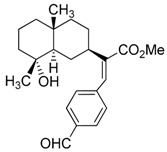 **5d**, 76%
5	X = Br	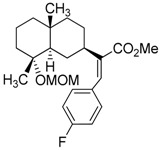 **4e**, 64%	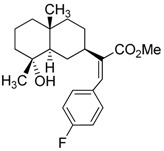 **5e**, 75%
6	X = I	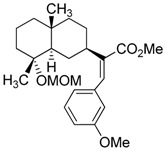 **4f**, 60%	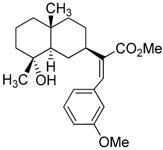 **5f**, 79%
7	X = I	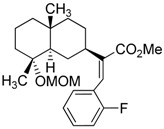 **4g**,—^(c)^	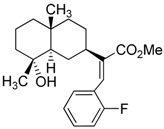 **5g**, 62%
8	X = Br	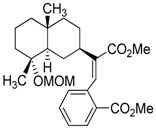 **4h**, 55%	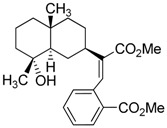 **5h**, 78%
9	X = Br	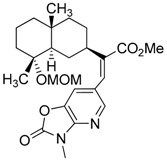 **4i**, 50%	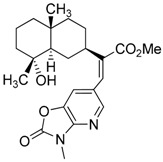 **5i**, 80%

**^a^** Experimental conditions: **3** (1 mmol), aryl-halide (1.1 mmol) dissolved in DMF (2 mL), 120 °C, for 24 h; ^b^ Isolated yields; ^c^ Compound **4g** was not observed during the reaction, **5g** was directly obtained.

## Supplementary Files

Supplementary File 1
