# Supplementary material for: Modification of Natural Eudesmane Scaffolds via Mizoroki-Heck Reactions
Source: Molecules. 2017 Apr 20;22(4):652. doi: 10.3390/molecules22040652 (PMC6153938; doi:10.3390/molecules22040652)

$^{1}$H NMR spectrum of methyl (2E)-2-[(2R,4aR,8R,8aR)-8-hydroxy-4a,8-dimethyl-decahydronaphthalen-2-yl]-prop-2-enoate (1)

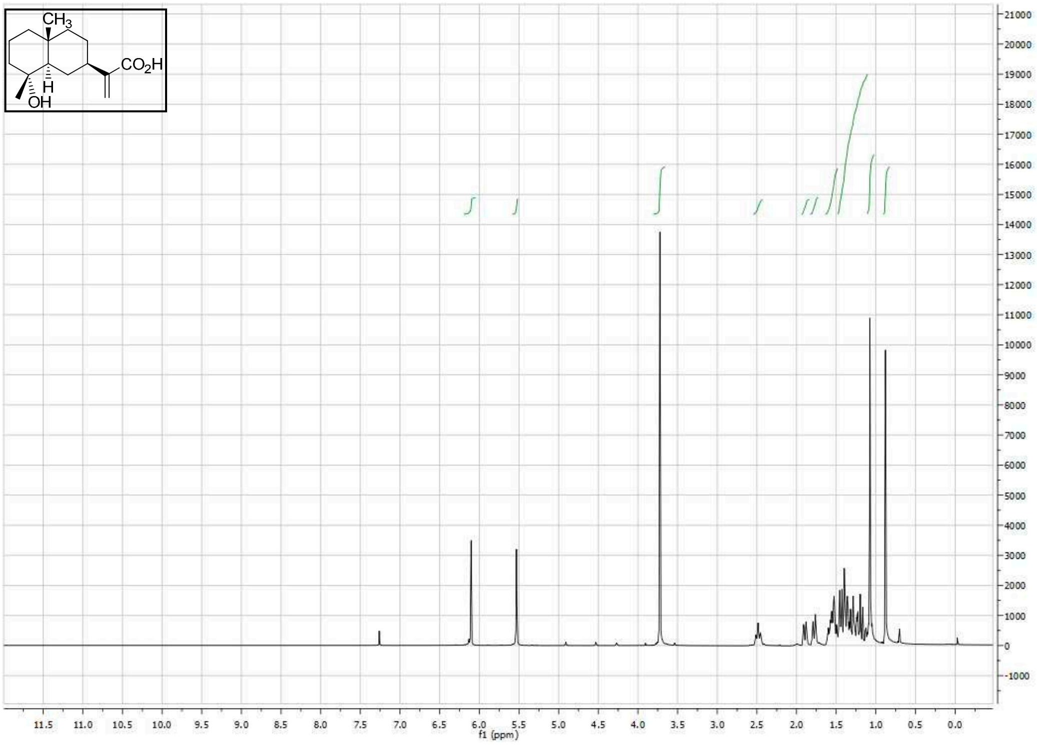


$^{13}$C NMR spectrum of methyl (2E)-2-[(2R,4aR,8R,8aR)-8-hydroxy-4a,8-dimethyl-decahydronaphthalen-2-yl]-prop-2-enoate (1)

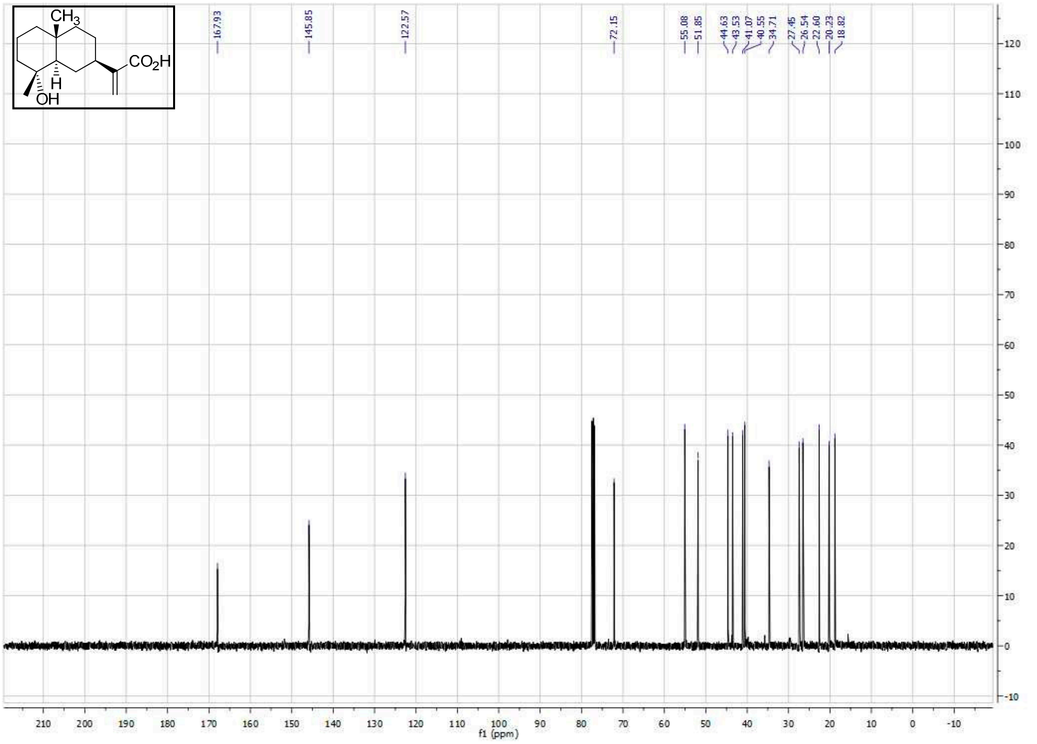

**$^{1}$H NMR spectrum of methyl (2E)-2-[(2R,4aR,8R,8aR)-8-(methoxymethoxy)-4a,8-dimethyl-decahydronaphthalen-2-yl]prop-2-enoate (3)**

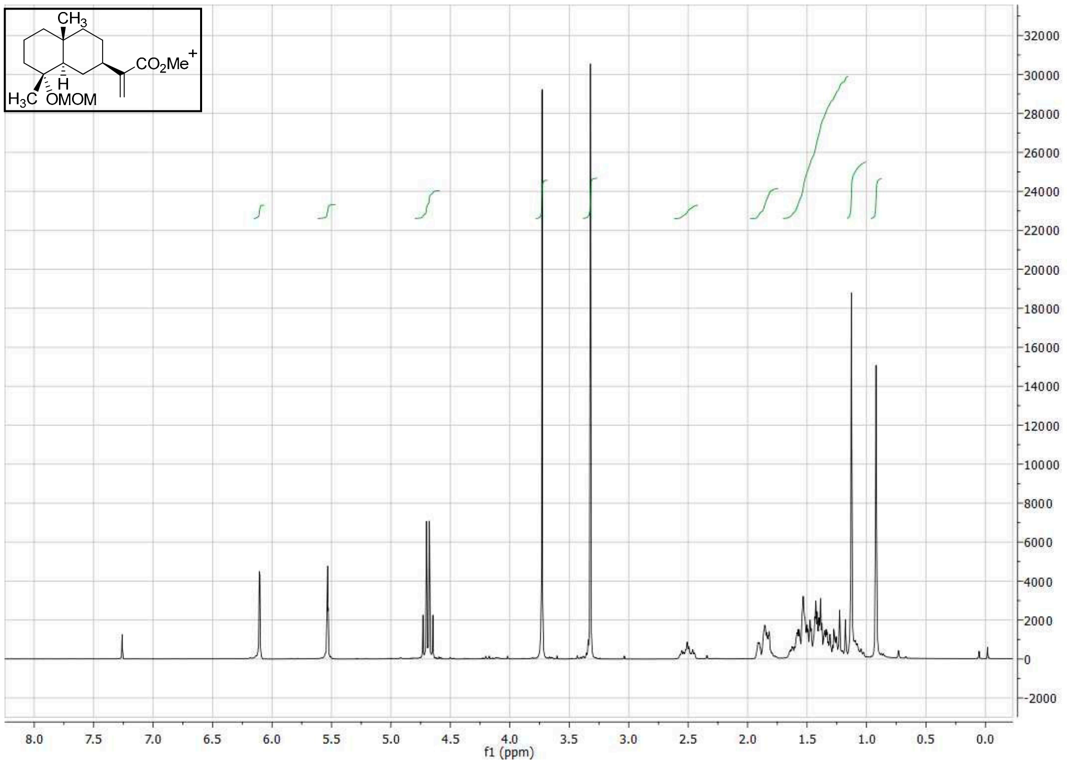


**$^{13}$C NMR spectrum of methyl (2E)-2-[(2R,4aR,8R,8aR)-8-(methoxymethoxy)-4a,8-dimethyl-decahydronaphthalen-2-yl]prop-2-enoate (3)**

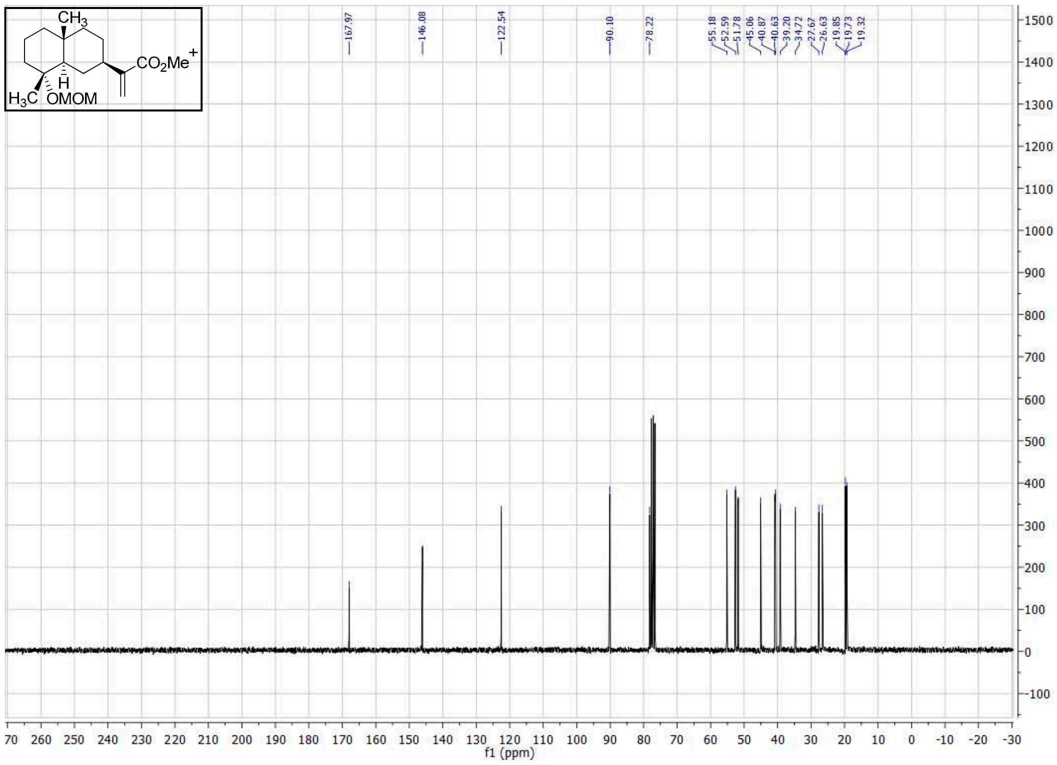

**$^{1}$H NMR spectrum of methyl (2E)-2-[(2R,4aR,8R,8aR)-8-(methoxymethoxy)-4a,8-dimethyl-decahydronaphthalen-2-yl]-3-phenylprop-2-enoate (4a)**

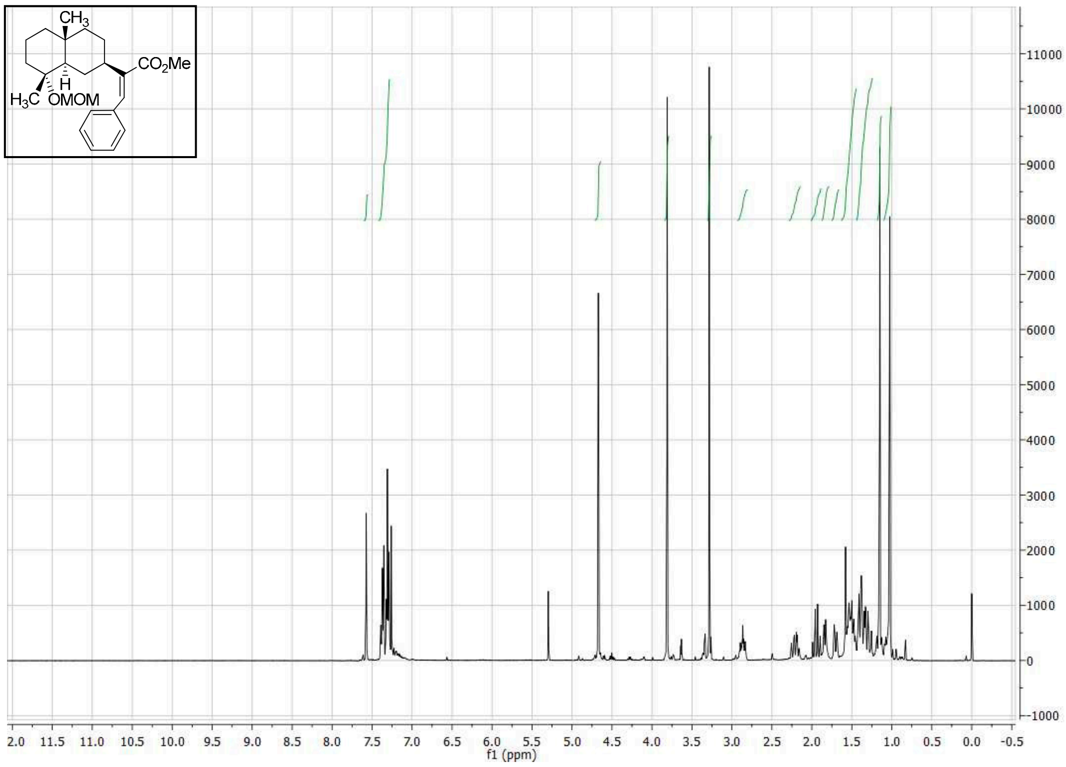


**$^{13}$C NMR spectrum of methyl (2E)-2-[(2R,4aR,8R,8aR)-8-(methoxymethoxy)-4a,8-dimethyl-decahydronaphthalen-2-yl]-3-phenylprop-2-enoate (4a)**

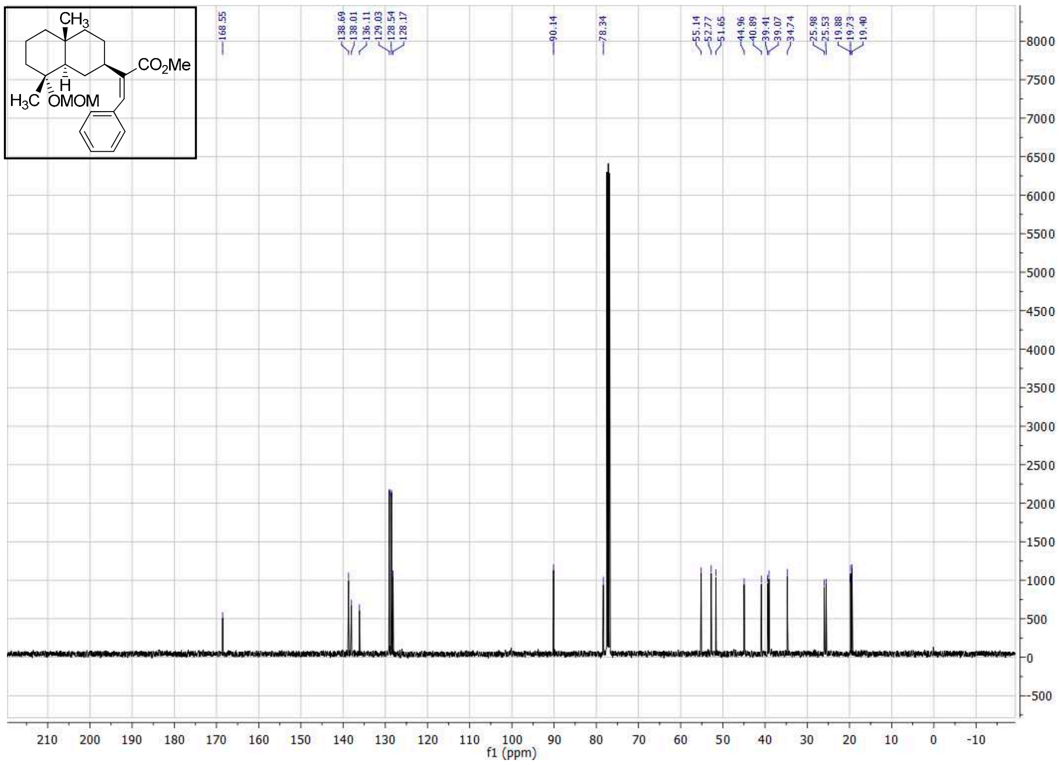

**$^{1}H$ NMR spectrum of methyl (2E)-2-[(2R,4aR,8R,8aR)-8-(methoxymethoxy)-4a,8-dimethyl-decahydronaphthalen-2-yl]-3-(4-methylphenyl)prop-2-enoate (4b)**

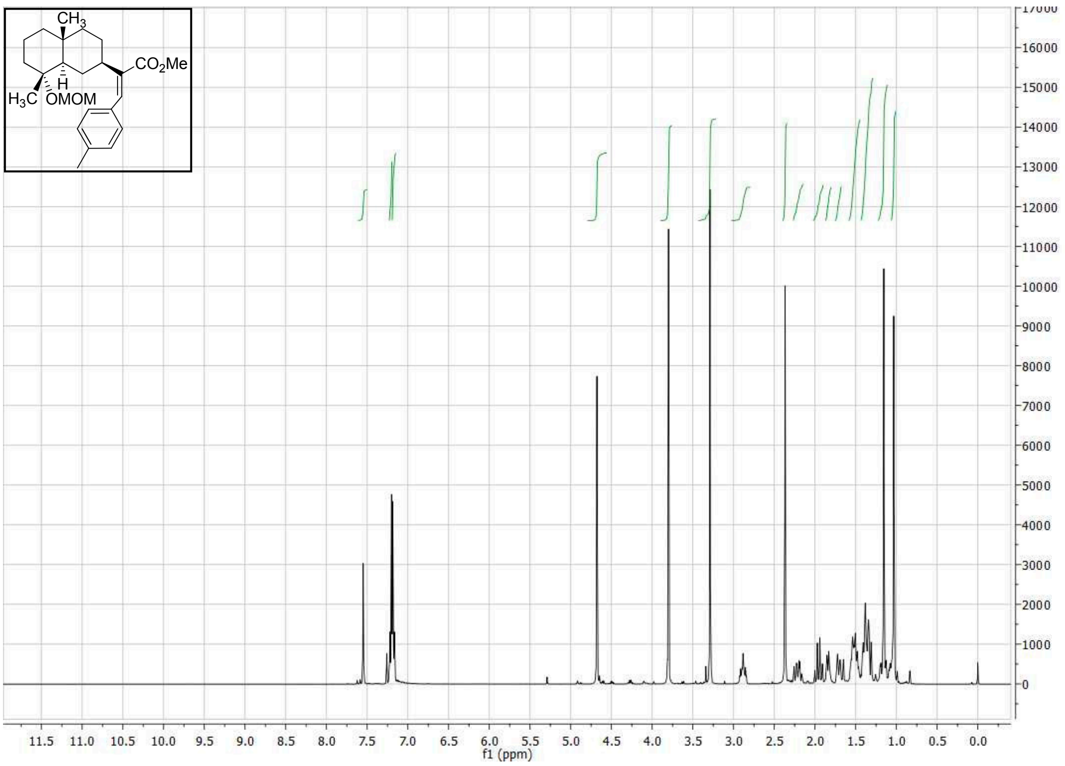


**$^{13}C$ NMR spectrum of methyl (2E)-2-[(2R,4aR,8R,8aR)-8-(methoxymethoxy)-4a,8-dimethyl-decahydronaphthalen-2-yl]-3-(4-methylphenyl)prop-2-enoate (4b)**

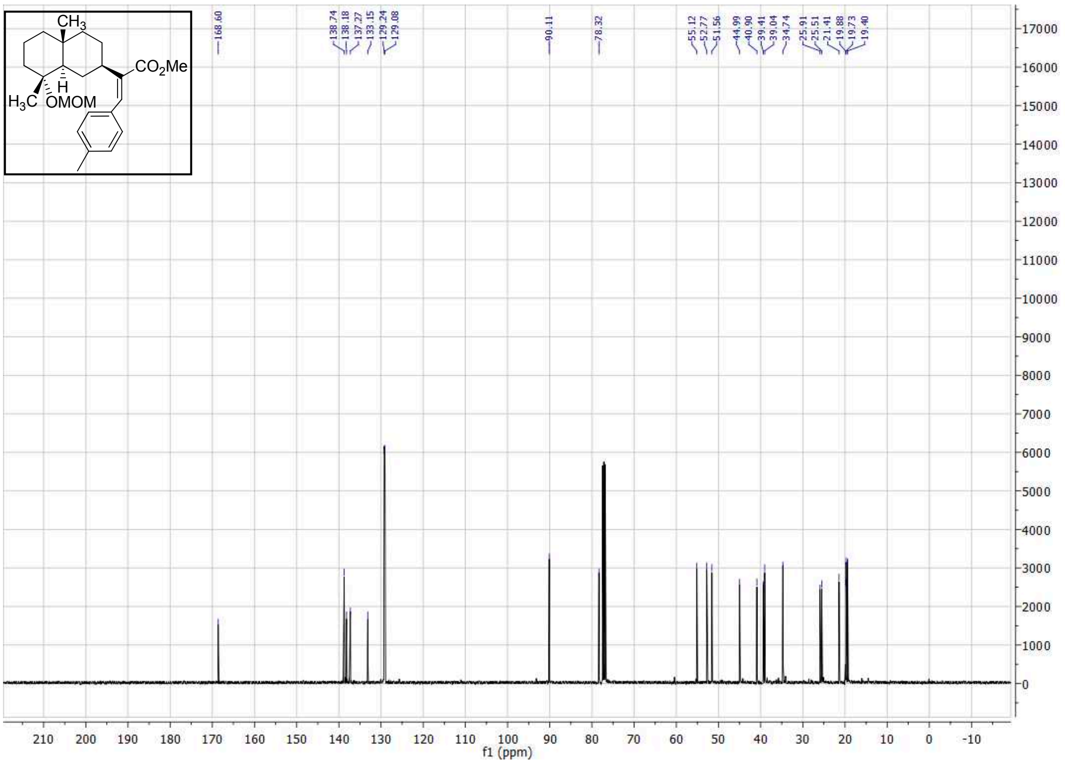

**$^1$H NMR spectrum of methyl (2E)-2-[(2R,4aR,8R,8aR)-8-(methoxymethoxy)-4a,8-dimethyl-decahydronaphthalen-2-yl]-3-(4-methoxyphenyl)prop-2-enoate (4c)**

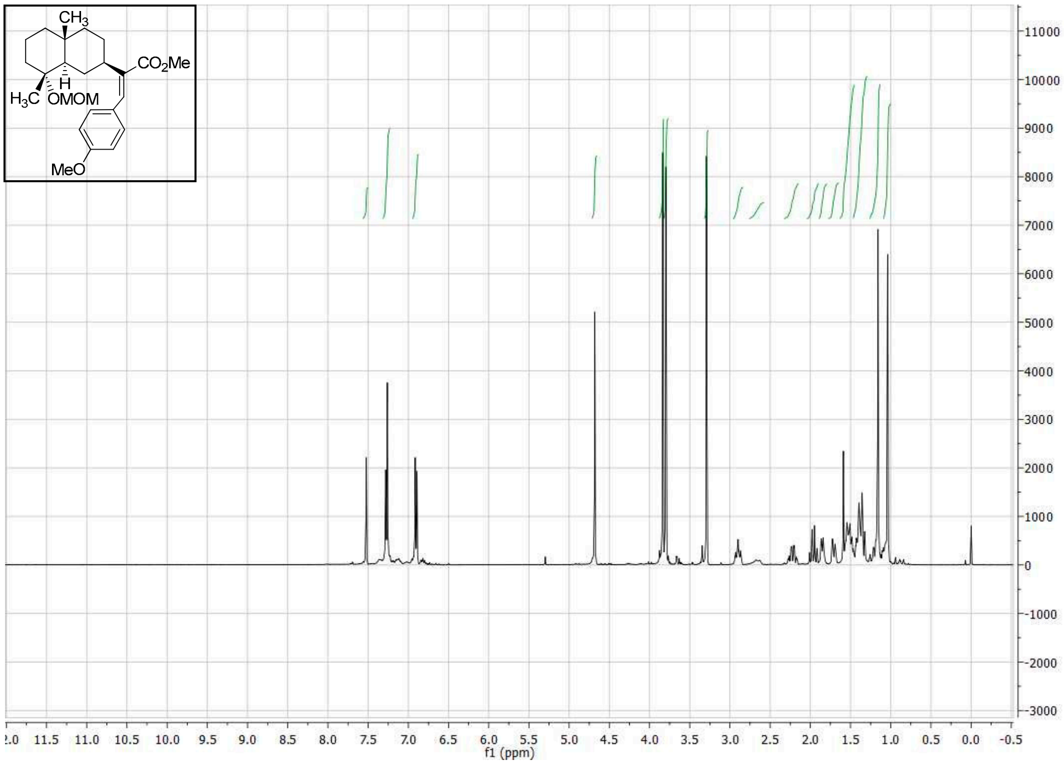


**$^{13}$C NMR spectrum of methyl (2E)-2-[(2R,4aR,8R,8aR)-8-(methoxymethoxy)-4a,8-dimethyl-decahydronaphthalen-2-yl]-3-(4-methoxyphenyl)prop-2-enoate (4c)**

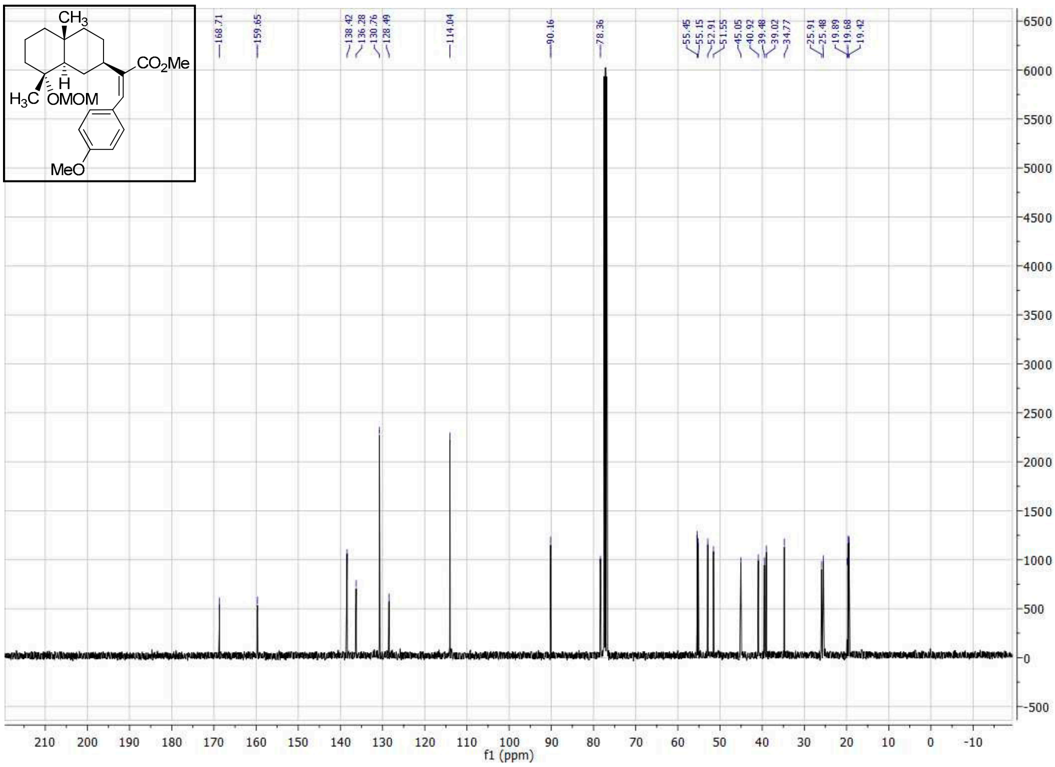

**$^{1}$H NMR spectrum of methyl (2E)-2-[(2R,4aR,8R,8aR)-8-(methoxymethoxy)-4a,8-dimethyl-decahydronaphthalen-2-yl]-3-(4-formylphenyl)prop-2-enoate (4d)**

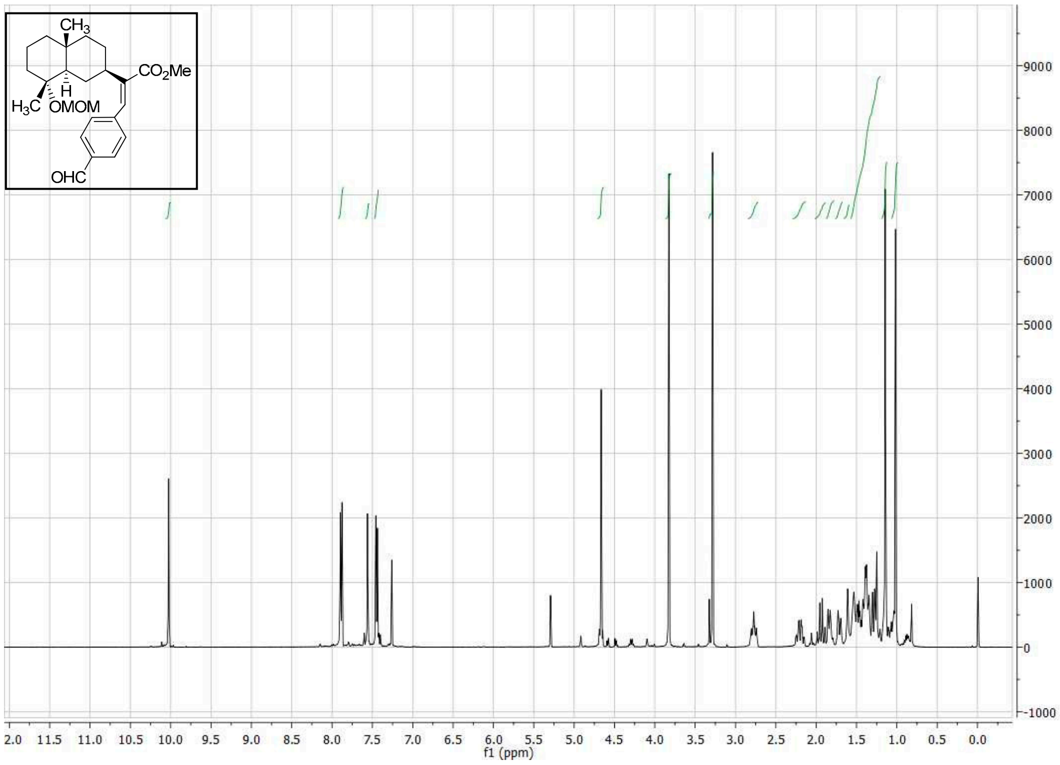


**$^{13}$C NMR spectrum of methyl (2E)-2-[(2R,4aR,8R,8aR)-8-(methoxymethoxy)-4a,8-dimethyl-decahydronaphthalen-2-yl]-3-(4-formylphenyl)prop-2-enoate (4d)**

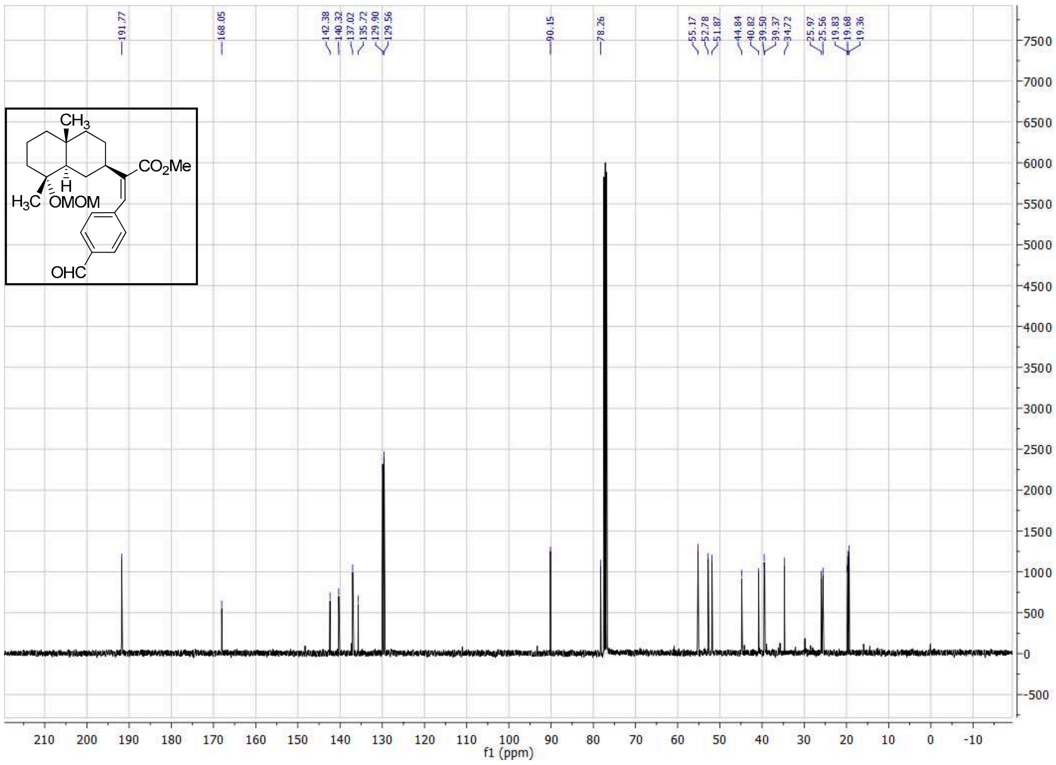

**$^{1}$H NMR spectrum of methyl (2E)-2-[(2R,4aR,8R,8aR)-8-(methoxymethoxy)-4a,8-dimethyl-decahydronaphthalen-2-yl]-3-(4-fluorophenyl)prop-2-enoate (4e)**

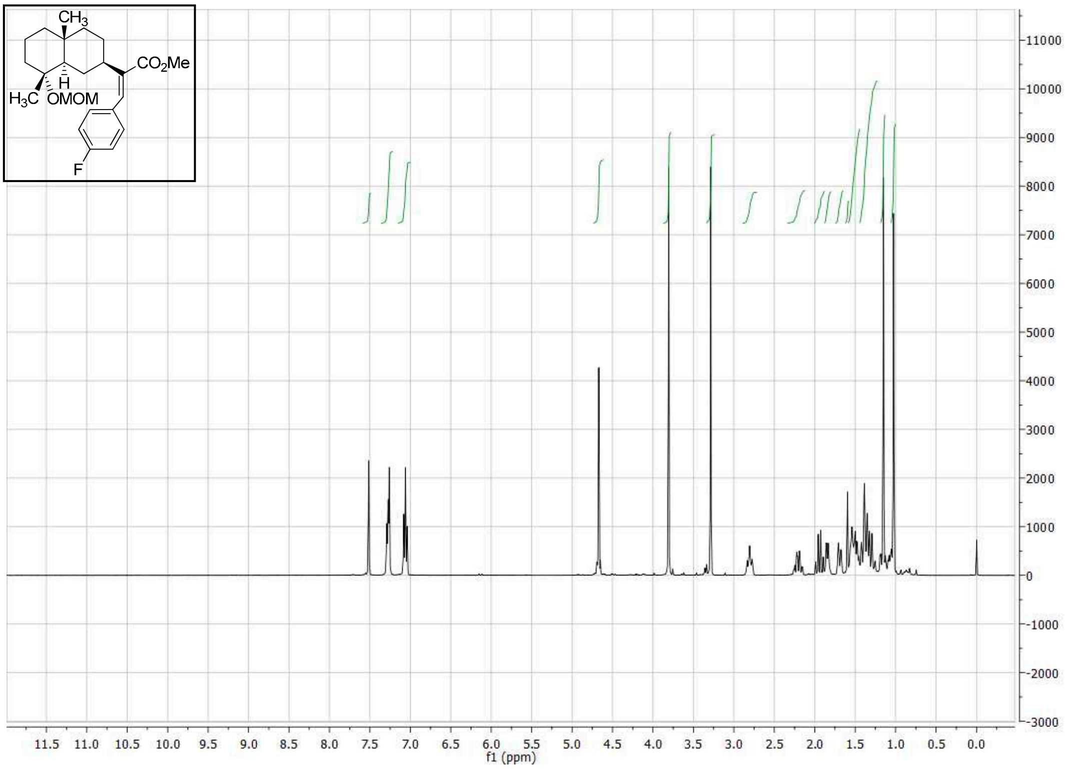


**$^{13}$C NMR spectrum of methyl (2E)-2-[(2R,4aR,8R,8aR)-8-(methoxymethoxy)-4a,8-dimethyl-decahydronaphthalen-2-yl]-3-(4-fluorophenyl)prop-2-enoate (4e)**

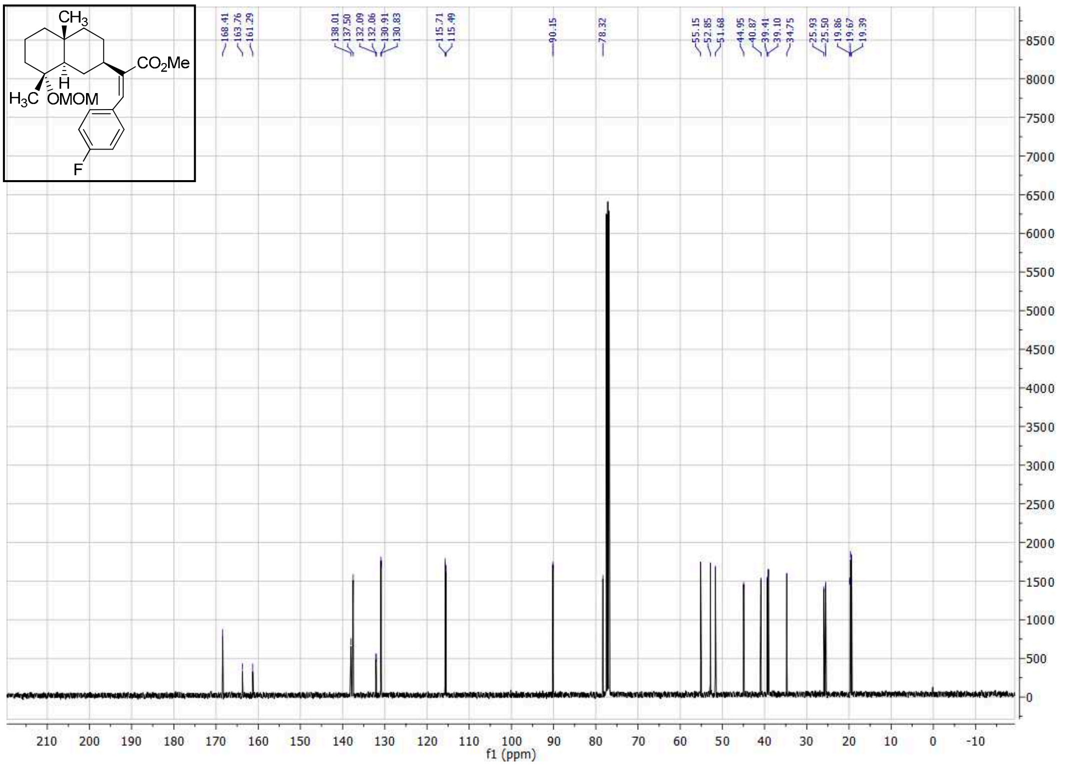

**$^{1}$H NMR spectrum of methyl (2E)-2-[(2R,4aR,8R,8aR)-8-(methoxymethoxy)-4a,8-dimethyl-decahydronaphthalen-2-yl]-3-(3-methoxyphenyl)prop-2-enoate (4f)**

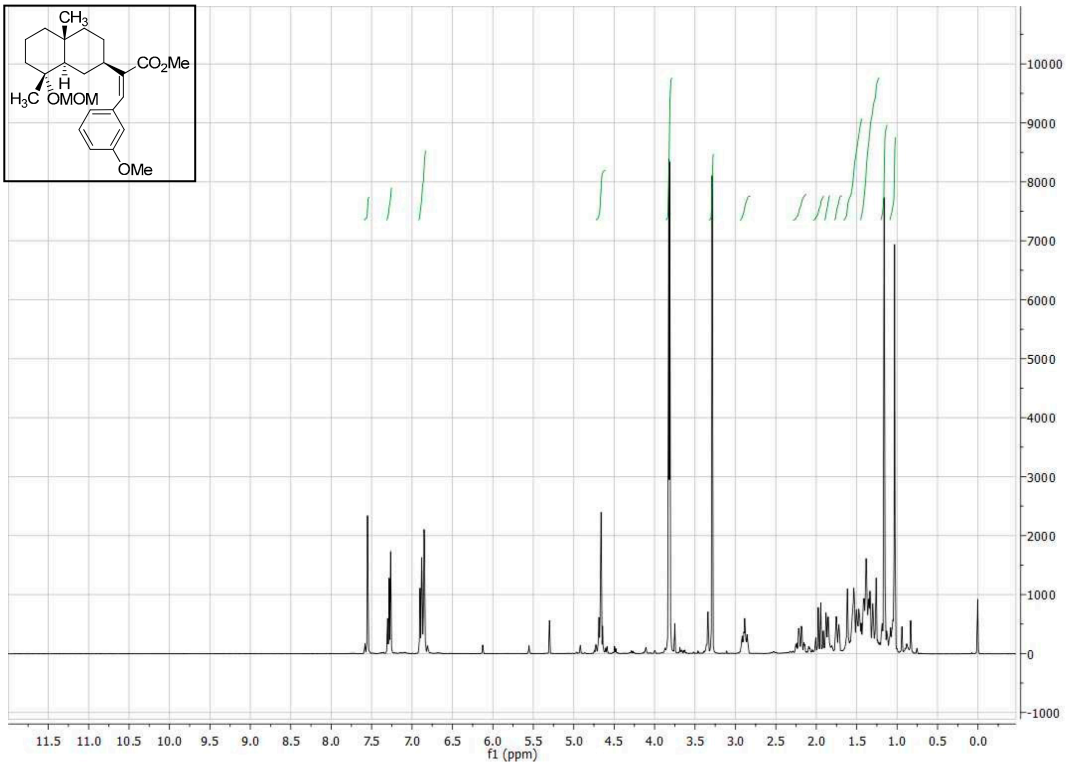


**$^{13}$C NMR spectrum of methyl (2E)-2-[(2R,4aR,8R,8aR)-8-(methoxymethoxy)-4a,8-dimethyl-decahydronaphthalen-2-yl]-3-(3-methoxyphenyl)prop-2-enoate (4f)**

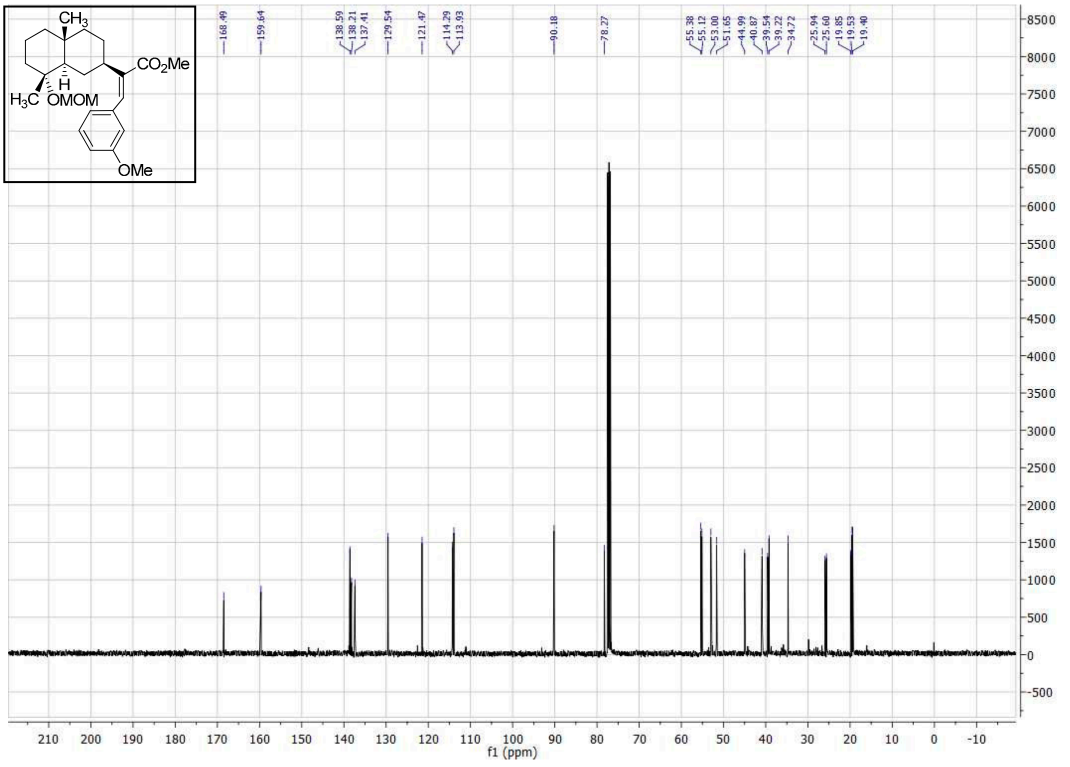

**$^{1}$H NMR spectrum of methyl 2-[(1E)-2-[(2R,4aR,8R,8aR)-8-(methoxymethoxy)-4a,8-dimethyl-decahydronaphthalen-2-yl]-3-methoxy-3-oxoprop-1-en-1-yl]benzoate (4h)**

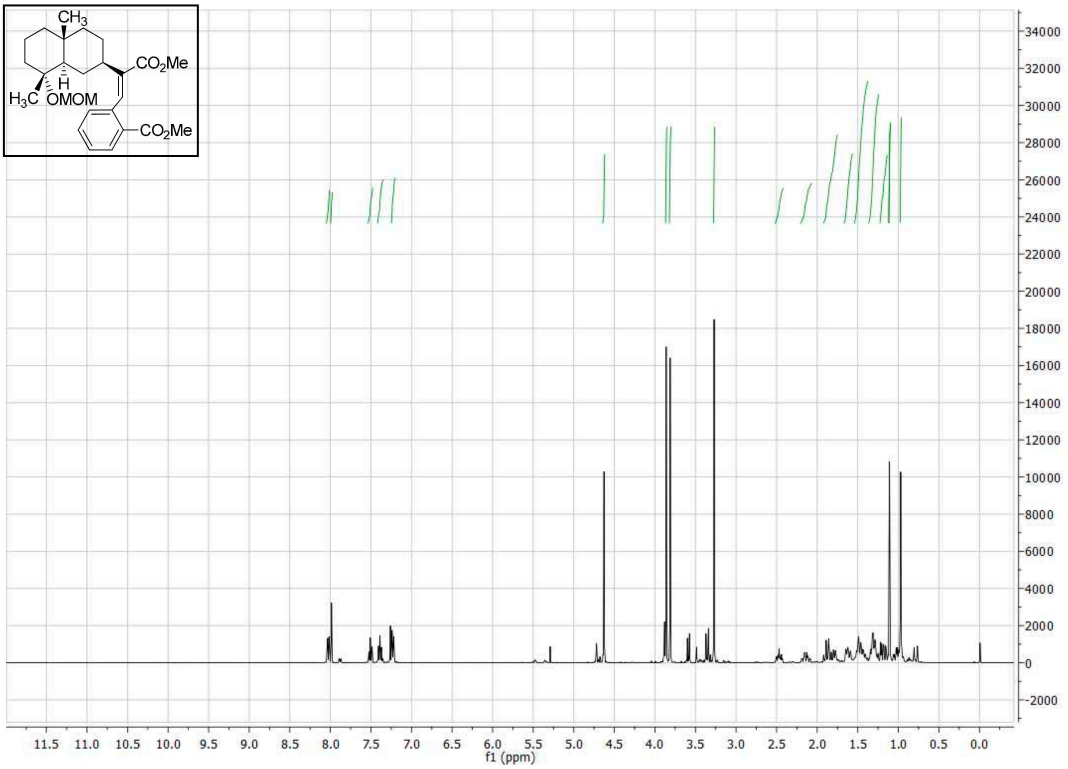


**$^{13}$C NMR spectrum of methyl 2-[(1E)-2-[(2R,4aR,8R,8aR)-8-(methoxymethoxy)-4a,8-dimethyl-decahydronaphthalen-2-yl]-3-methoxy-3-oxoprop-1-en-1-yl]benzoate (4h)**

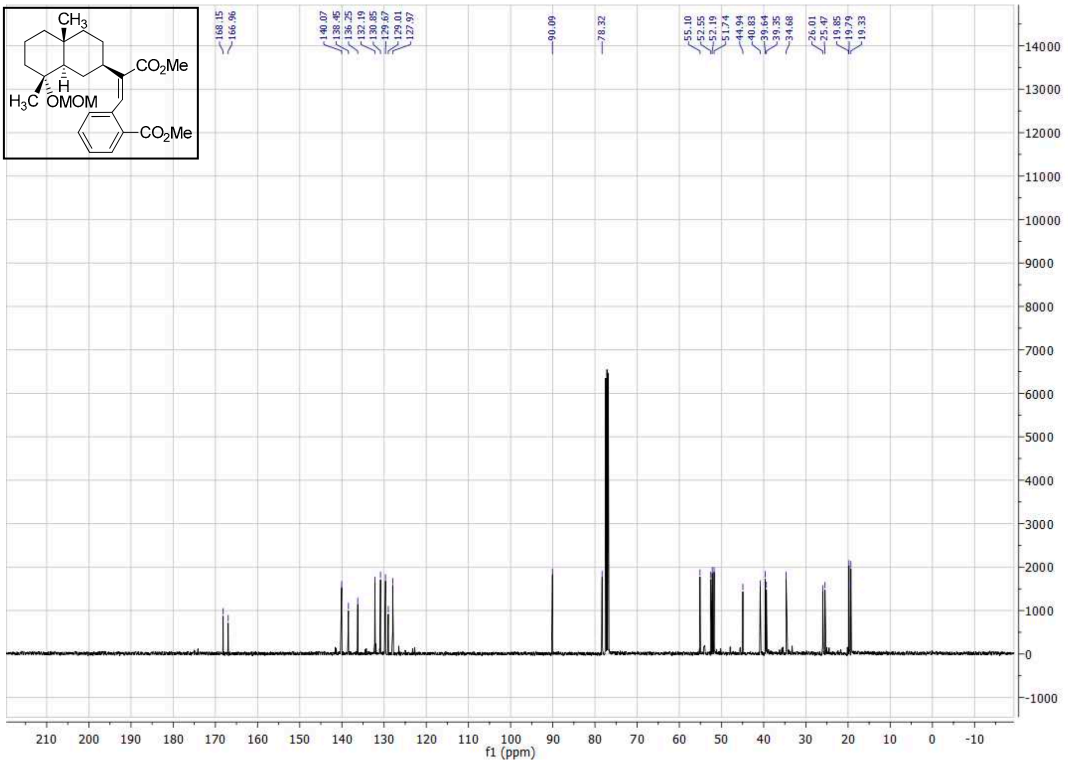

**$^{1}$H NMR spectrum of methyl (2E)-2-[(2R,4aR,8R,8aR)-8-(methoxymethoxy)-4a,8-dimethyl-decahydronaphthalen-2-yl]-3-{3-methyl-2-oxo-2H,3H-[1,3oxazolo[4,5-*b*]pyridine-6-yl}prop-2-enoate (4i)**

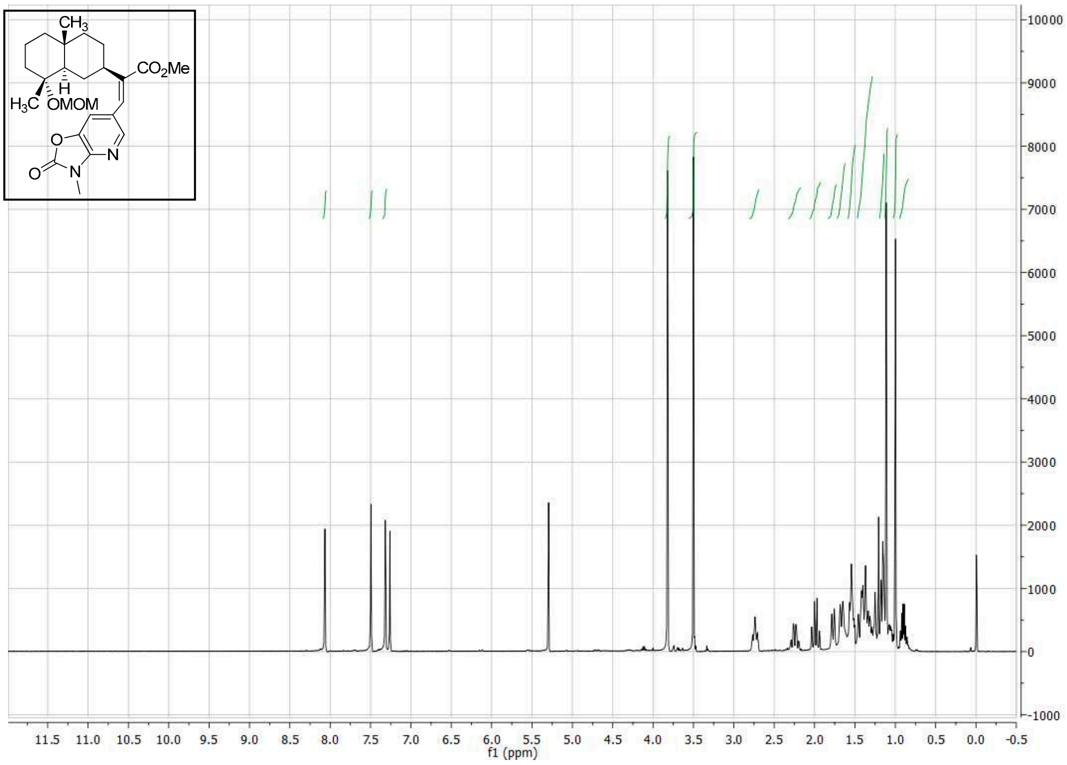


**$^{13}$C NMR spectrum of methyl (2E)-2-[(2R,4aR,8R,8aR)-8-(methoxymethoxy)-4a,8-dimethyl-decahydronaphthalen-2-yl]-3-{3-methyl-2-oxo-2H,3H-[1,3oxazolo[4,5-*b*]pyridine-6-yl}prop-2-enoate (4i)**

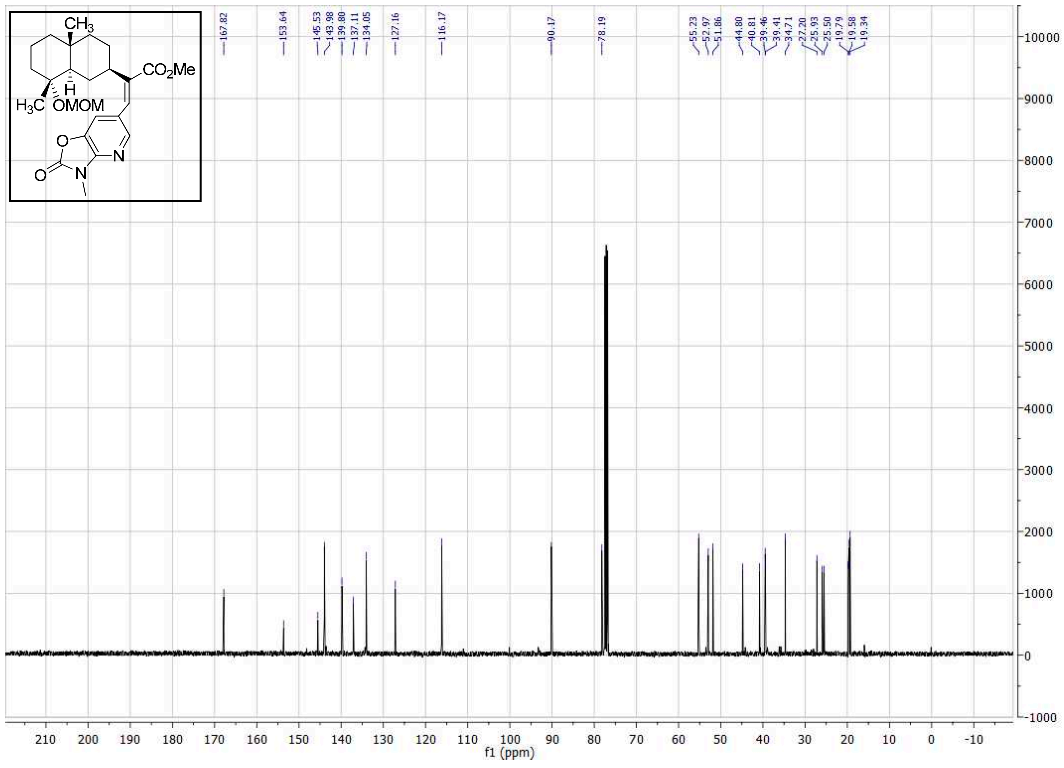

**$^{1}$H NMR spectrum of methyl (2E)-2-[(2R,4aR,8R,8aR)-8-hydroxy-4a,8-dimethyl-decahydronaphthalen-2-yl]-3-phenylprop-2-enoate (5a)**

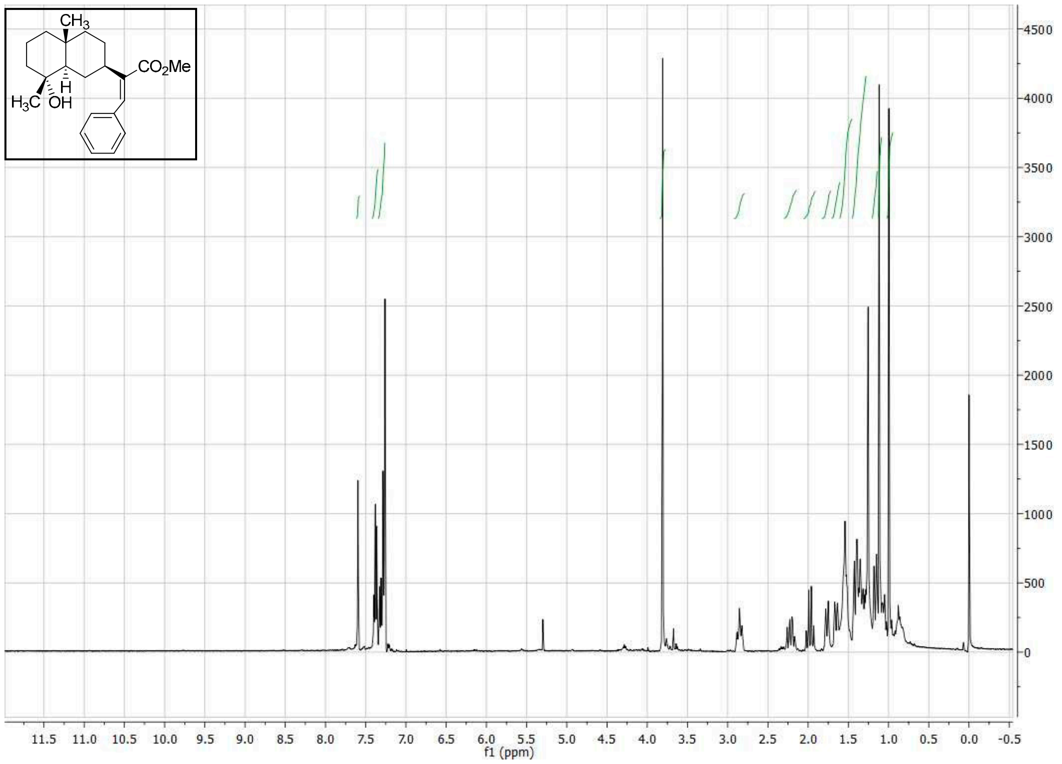


**$^{13}$C NMR spectrum of methyl (2E)-2-[(2R,4aR,8R,8aR)-8-hydroxy-4a,8-dimethyl-decahydronaphthalen-2-yl]-3-phenylprop-2-enoate (5a)**

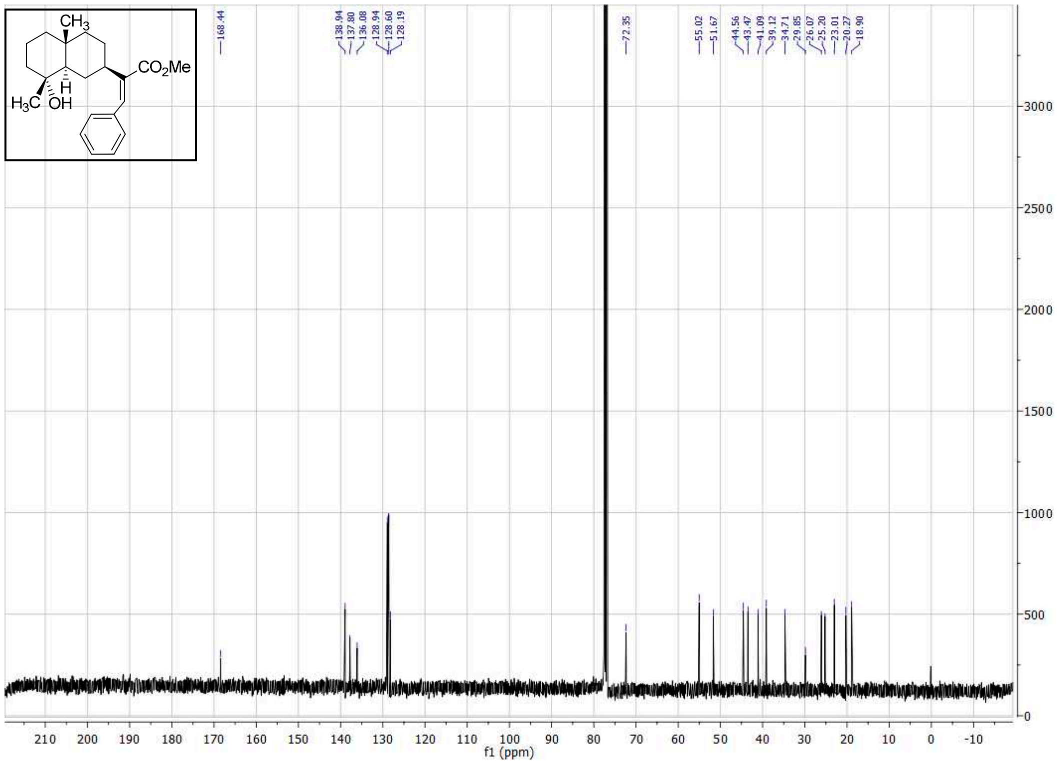

**$^{1}$H NMR spectrum of methyl (2E)-2-[(2R,4aR,8R,8aR)-8-hydroxy-4a,8-dimethyl-decahydronaphthalen-2-yl]-3-(4-methylphenyl)prop-2-enoate (5b)**

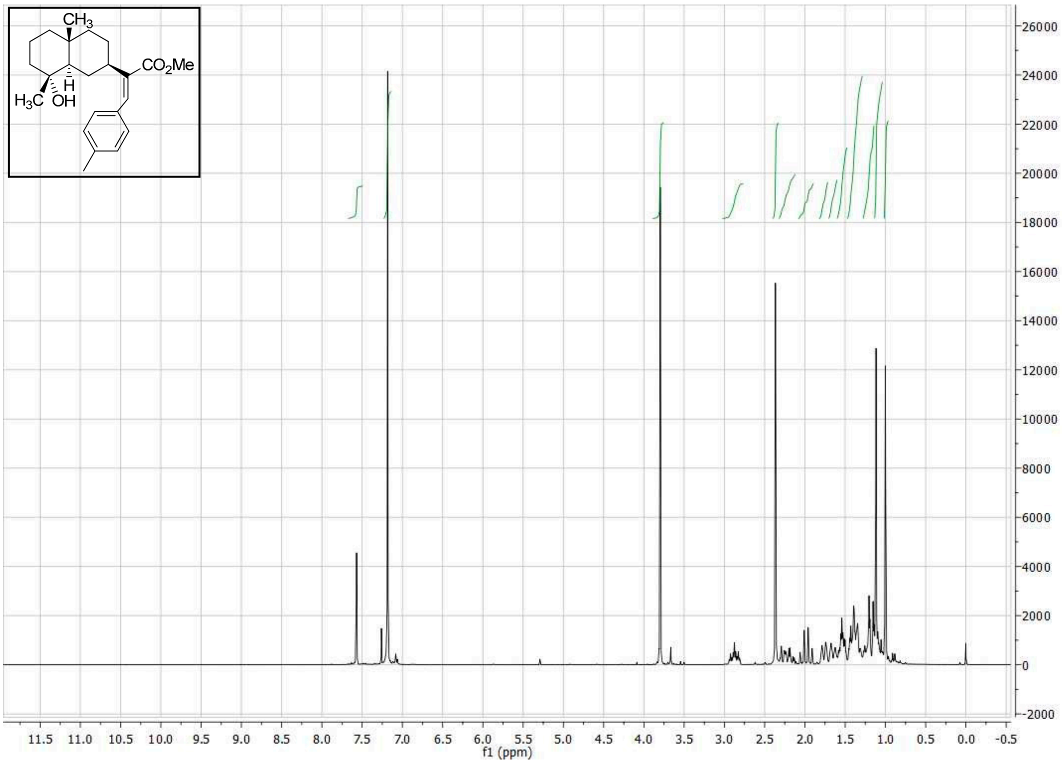

**$^{13}$C NMR spectrum of methyl (2E)-2-[(2R,4aR,8R,8aR)-8-hydroxy-4a,8-dimethyl-decahydronaphthalen-2-yl]-3-(4-methylphenyl)prop-2-enoate (5b)**

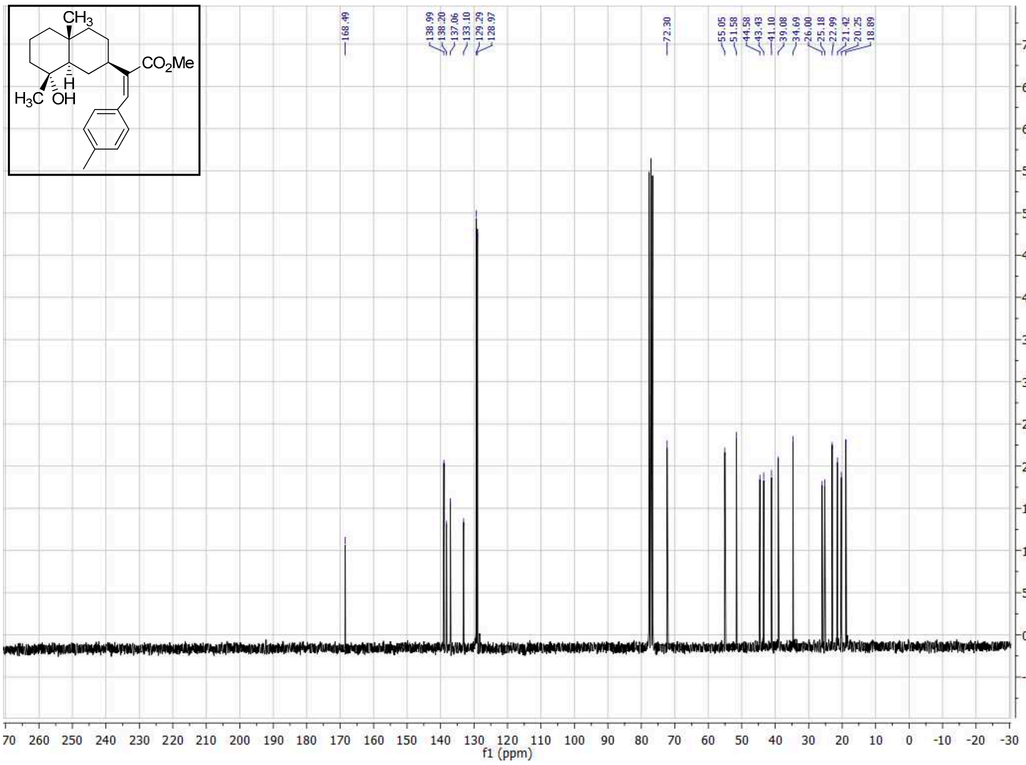

**COSY NMR spectrum of methyl (2E)-2-[(2R,4aR,8R,8aR)-8-hydroxy-4a,8-dimethyl-decahydronaphthalen-2-yl]-3-(4-methylphenyl)prop-2-enoate (5b)**

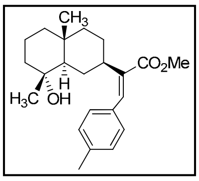


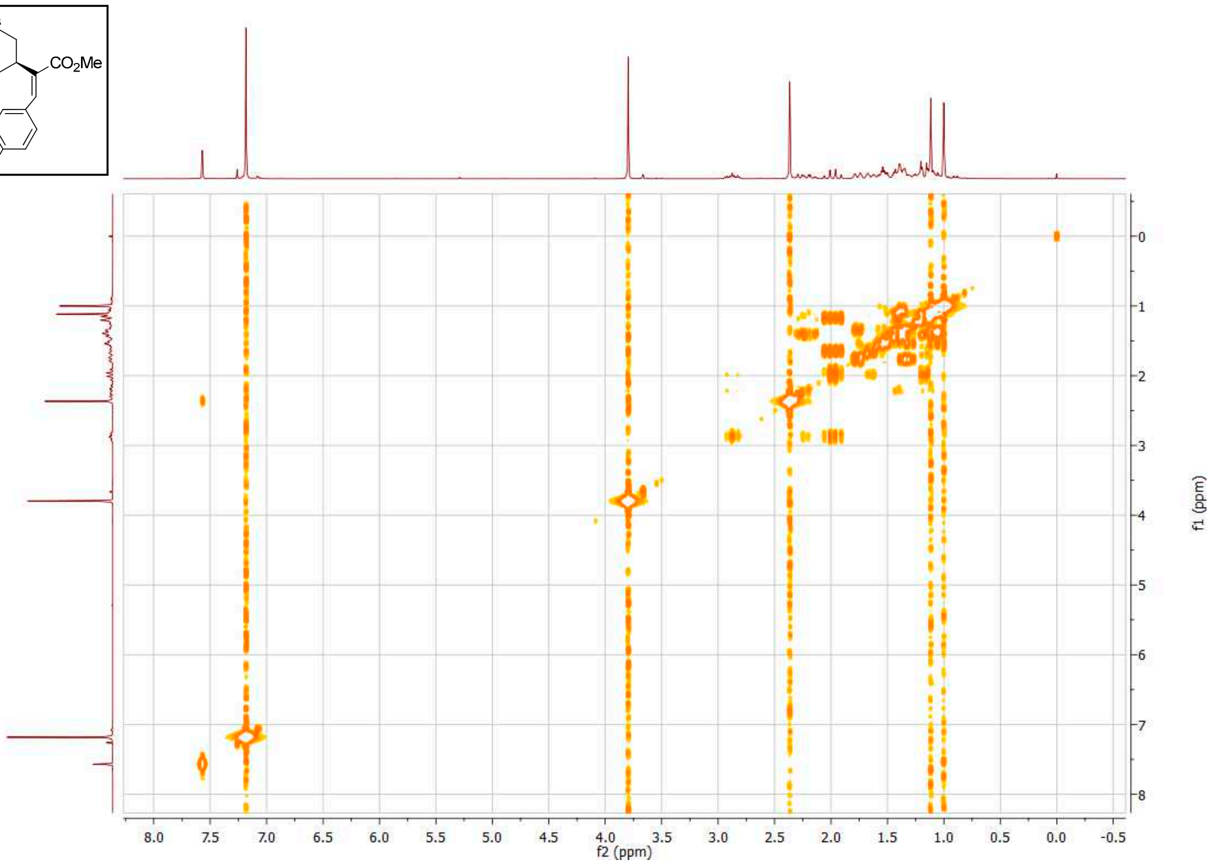

**NOESY NMR spectrum of methyl (2E)-2-[(2R,4aR,8R,8aR)-8-hydroxy-4a,8-dimethyl-decahydronaphthalen-2-yl]-3-(4-methylphenyl)prop-2-enoate (5b)**

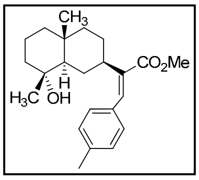


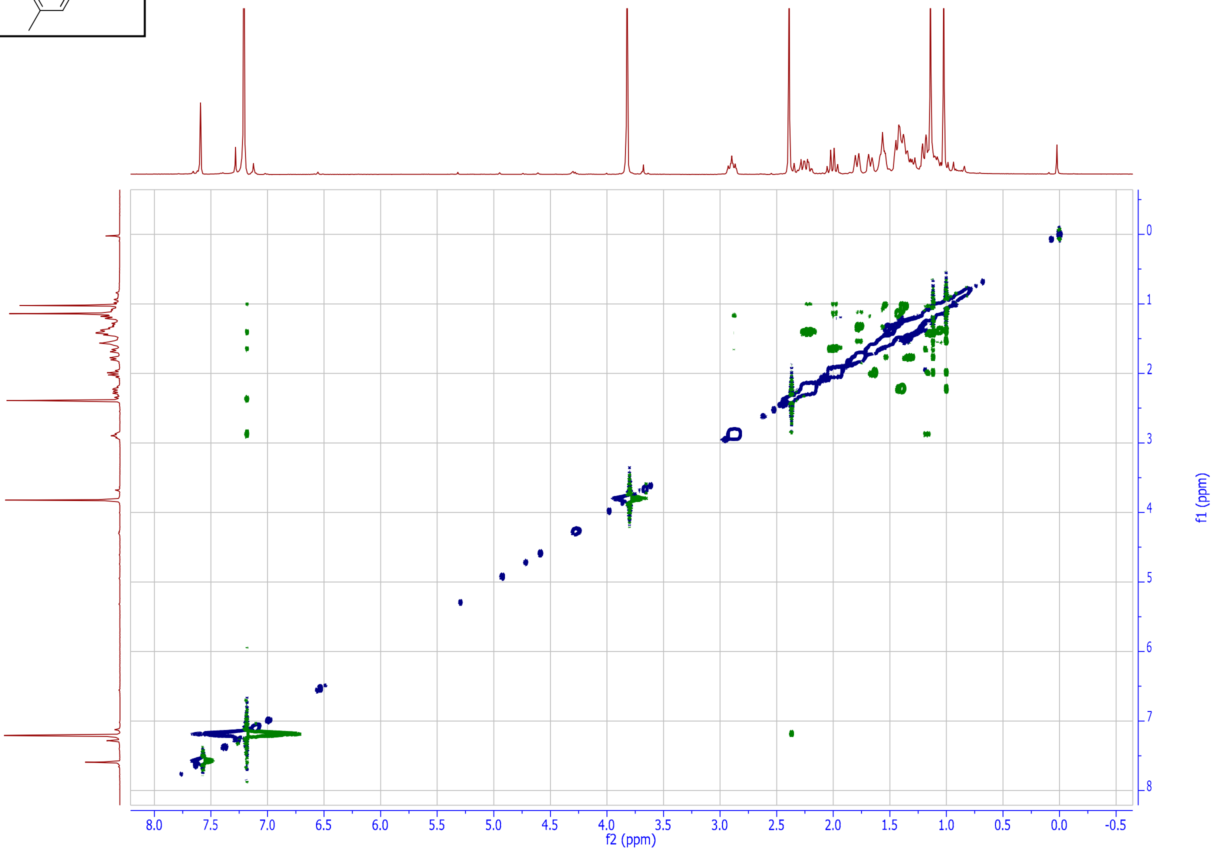

**$^{1}$H NMR spectrum of methyl (2E)-2-[(2R,4aR,8R,8aR)-8-hydroxy-4a,8-dimethyl-decahydronaphthalen-2-yl]-3-(4-methoxyphenyl)prop-2-enoate (5c)**

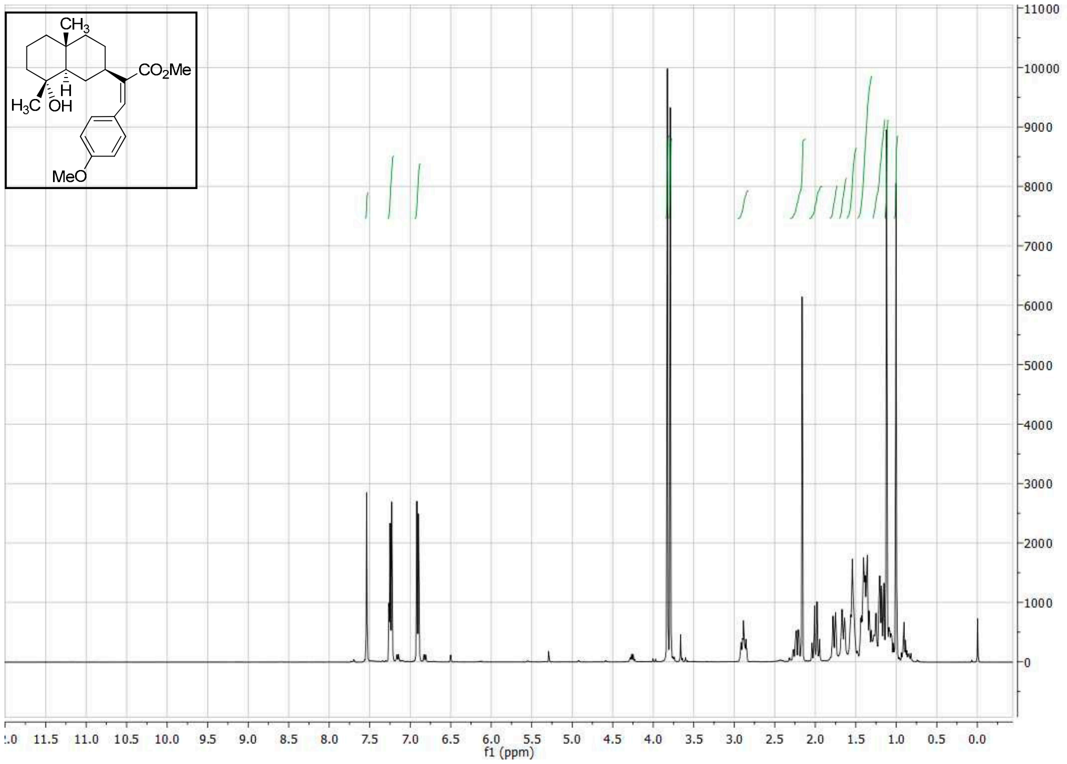


**$^{13}$C NMR spectrum of methyl (2E)-2-[(2R,4aR,8R,8aR)-8-hydroxy-4a,8-dimethyl-decahydronaphthalen-2-yl]-3-(4-methoxyphenyl)prop-2-enoate (5c)**

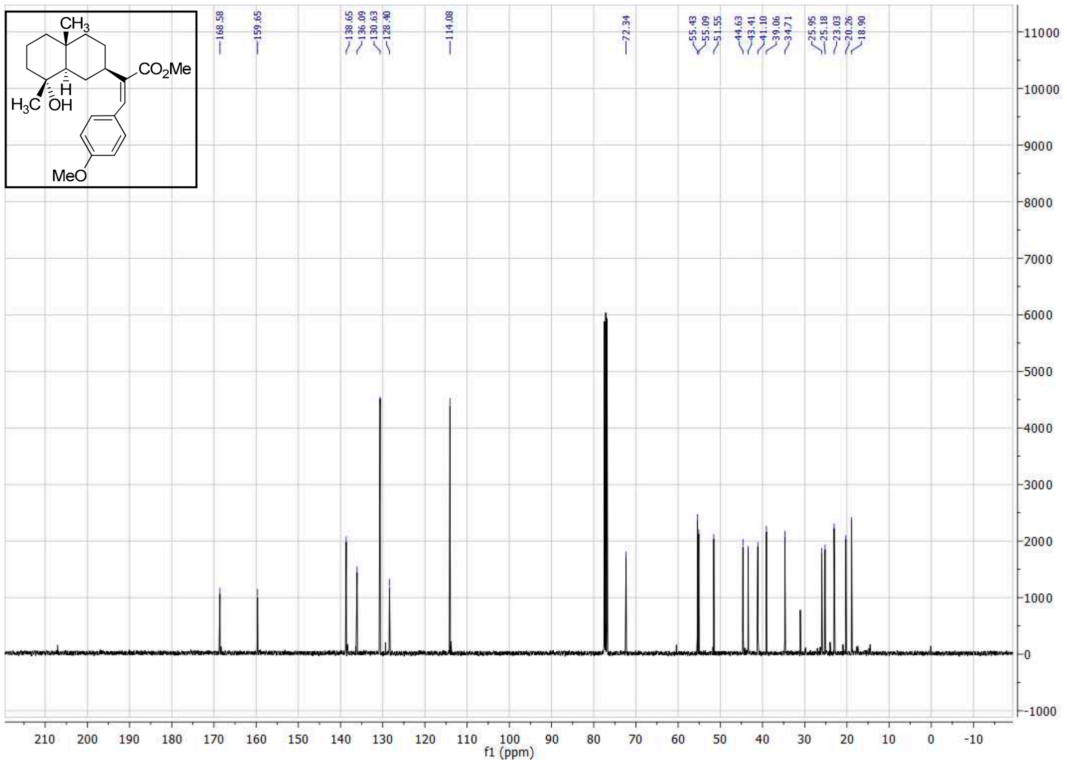

**$^{1}$H NMR spectrum of methyl (2E)-2-[(2R,4aR,8R,8aR)-8-hydroxy-4a,8-dimethyl-decahydronaphthalen-2-yl]-3-(4-formylphenyl)prop-2-enoate (5d)**

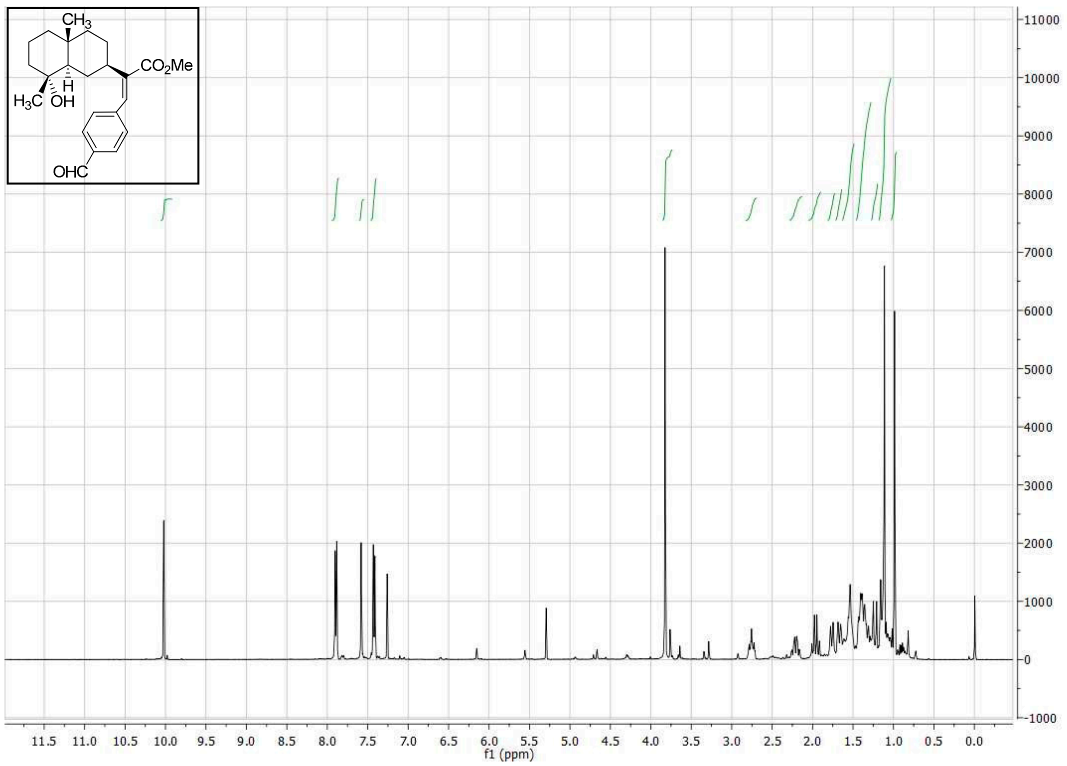

**$^{13}$C NMR spectrum of methyl (2E)-2-[(2R,4aR,8R,8aR)-8-hydroxy-4a,8-dimethyl-decahydronaphthalen-2-yl]-3-(4-formylphenyl)prop-2-enoate (5d)**

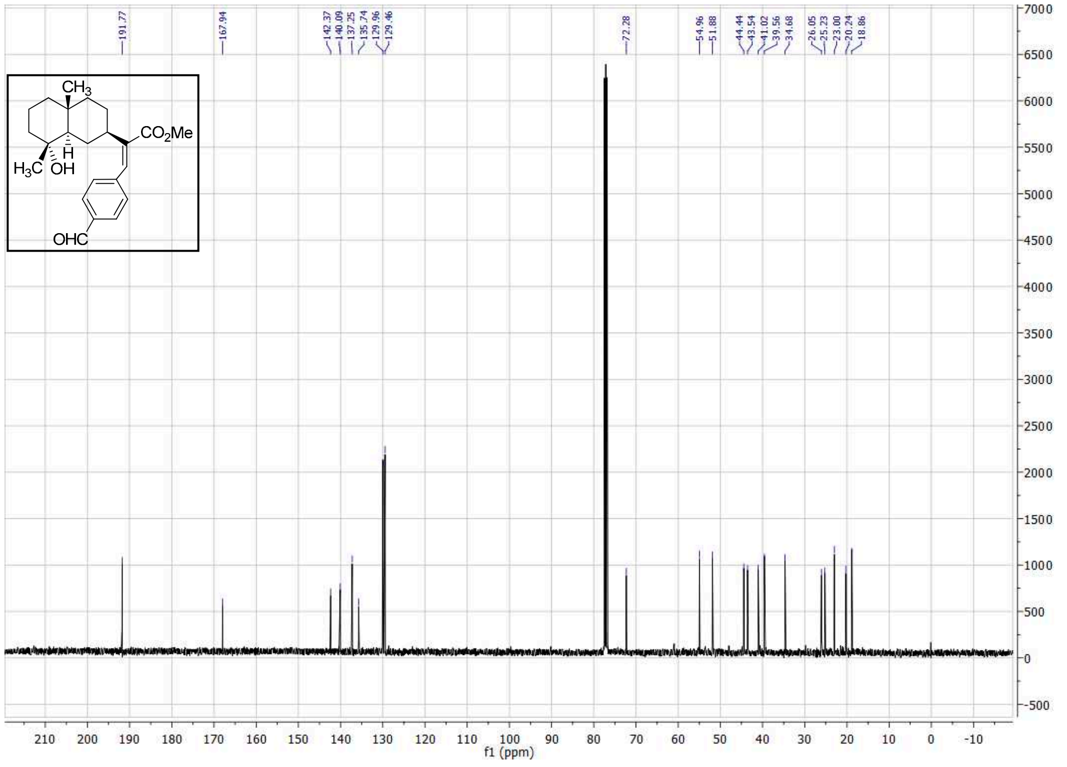

**$^{1}H$ NMR spectrum of methyl (2E)-2-[(2R,4aR,8R,8aR)-8-hydroxy-4a,8-dimethyl-decahydronaphthalen-2-yl]-3-(4-fluorophenyl)prop-2-enoate (5e)**

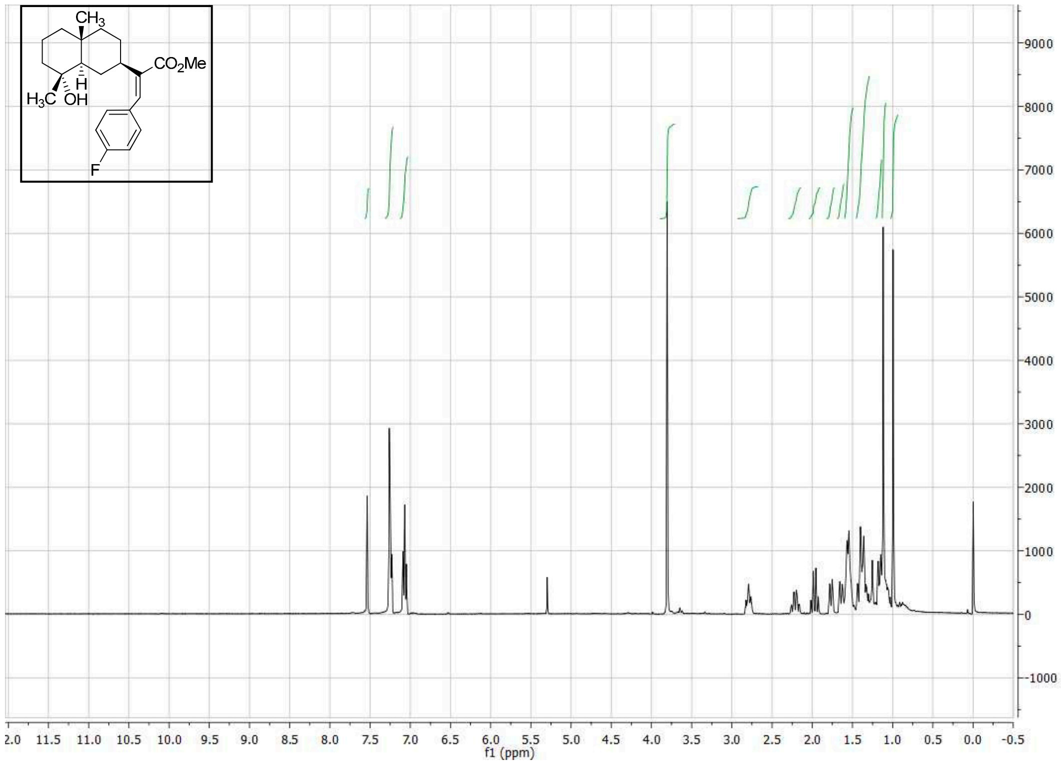


**$^{13}C$ NMR spectrum of methyl (2E)-2-[(2R,4aR,8R,8aR)-8-hydroxy-4a,8-dimethyl-decahydronaphthalen-2-yl]-3-(4-fluorophenyl)prop-2-enoate (5e)**

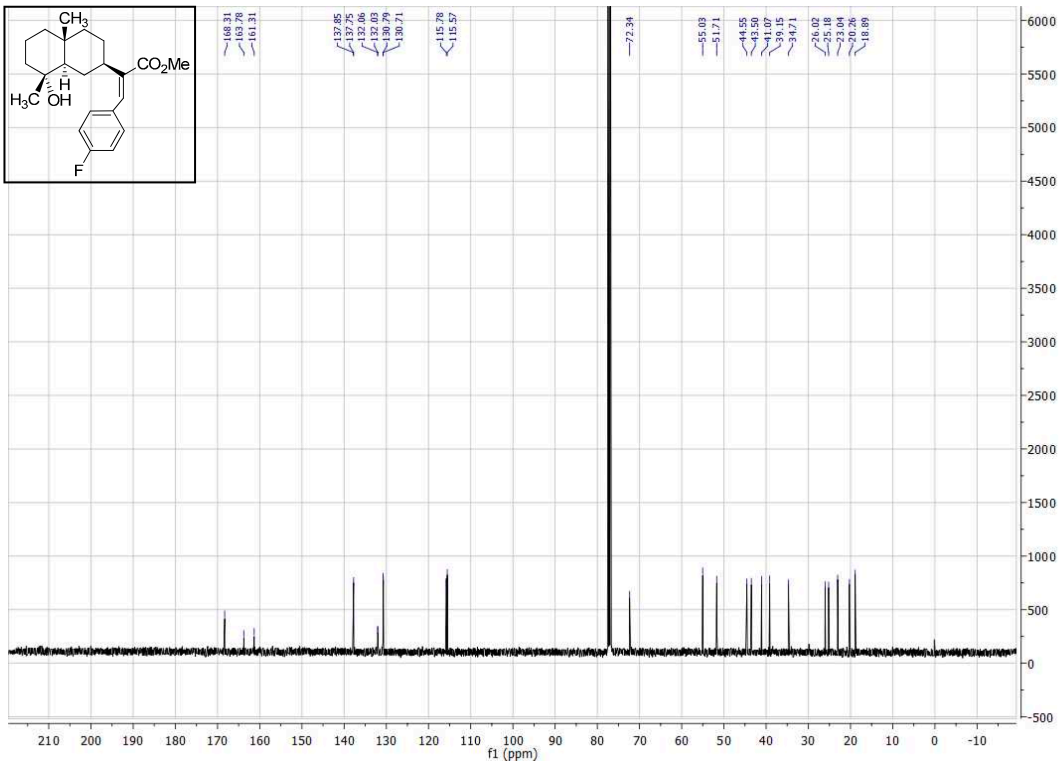

$^{1}$H NMR spectrum of methyl (2E)-2-[(2R,4aR,8R,8aR)-8-hydroxy-4a,8-dimethyl-decahydronaphthalen-2-yl]-3-(3-methoxyphenyl)prop-2-enoate (5f)

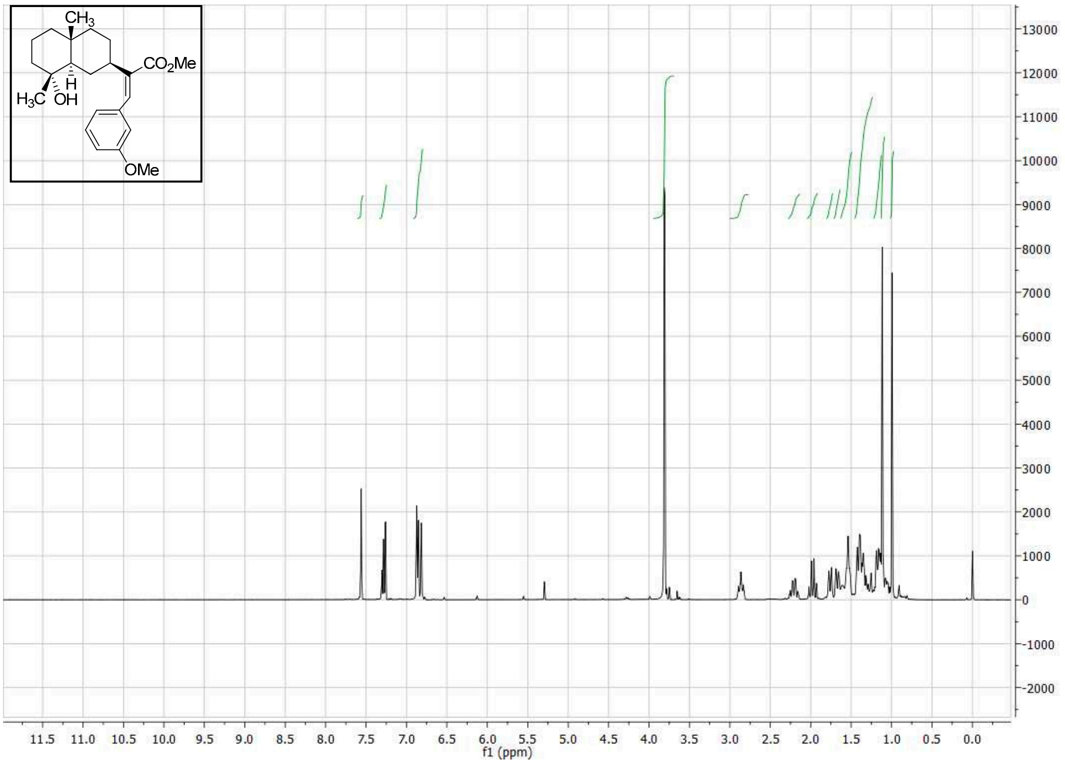

$^{13}$C NMR spectrum of methyl (2E)-2-[(2R,4aR,8R,8aR)-8-hydroxy-4a,8-dimethyl-decahydronaphthalen-2-yl]-3-(3-methoxyphenyl)prop-2-enoate (5f)

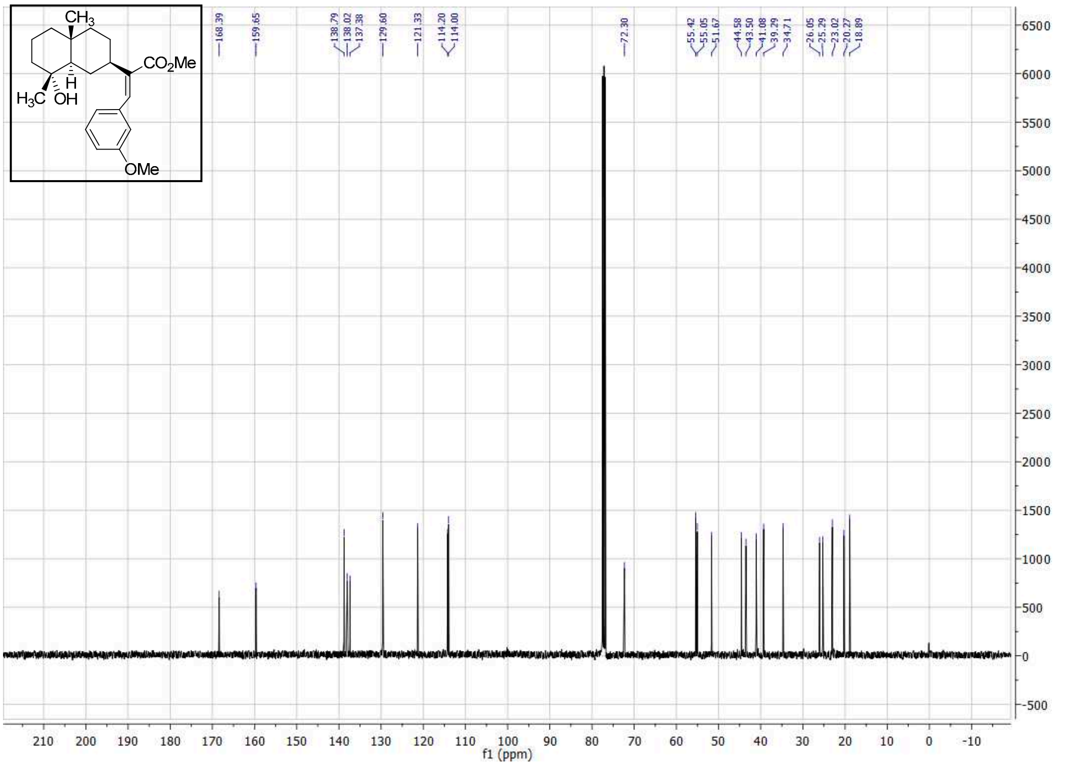

**$^{1}H$ NMR spectrum of methyl (2E)-2-[(2R,4aR,8R,8aR)-8-hydroxy-4a,8-dimethyl-decahydronaphthalen-2-yl]-3-(2-fluorophenyl)prop-2-enoate (5g)**

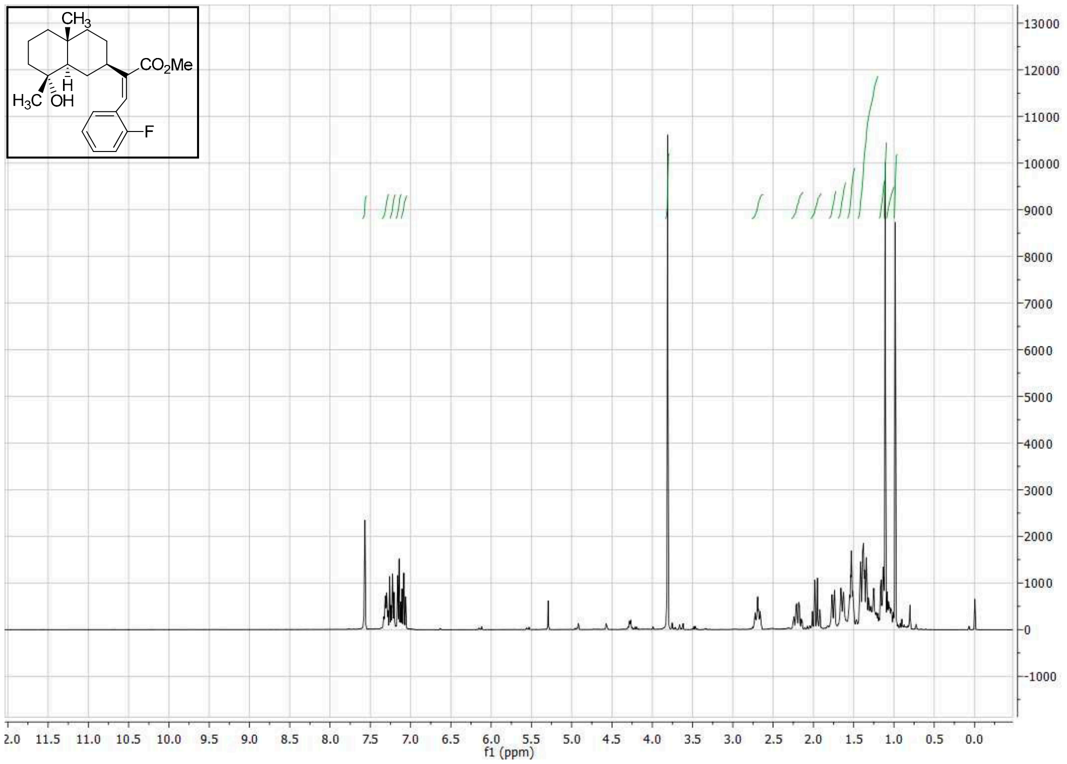


**$^{13}C$ NMR spectrum of methyl (2E)-2-[(2R,4aR,8R,8aR)-8-hydroxy-4a,8-dimethyl-decahydronaphthalen-2-yl]-3-(2-fluorophenyl)prop-2-enoate (5g)**

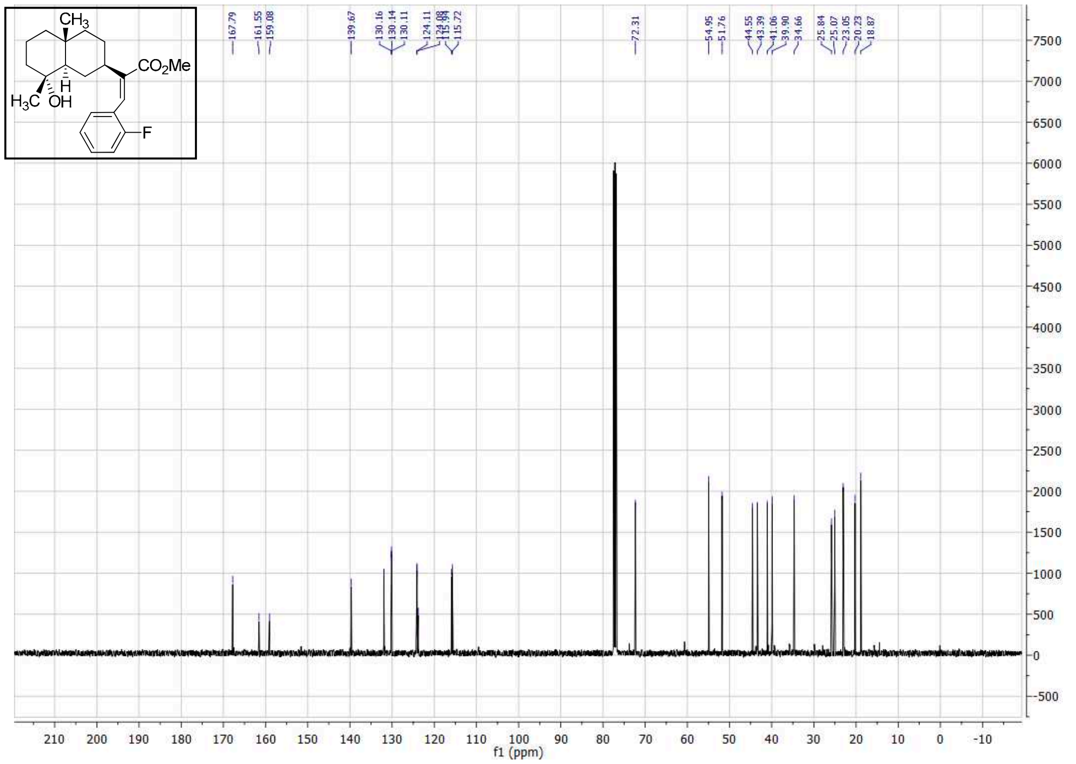

**$^{1}H$ NMR spectrum of methyl 2-[(1E)-2-[(2R,4aR,8R,8aR)-8-hydroxy-4a,8-dimethyl-decahydronaphthalen-2-yl]-3-methoxy-3-oxoprop-1-en-1-yl]benzoate (5h)**

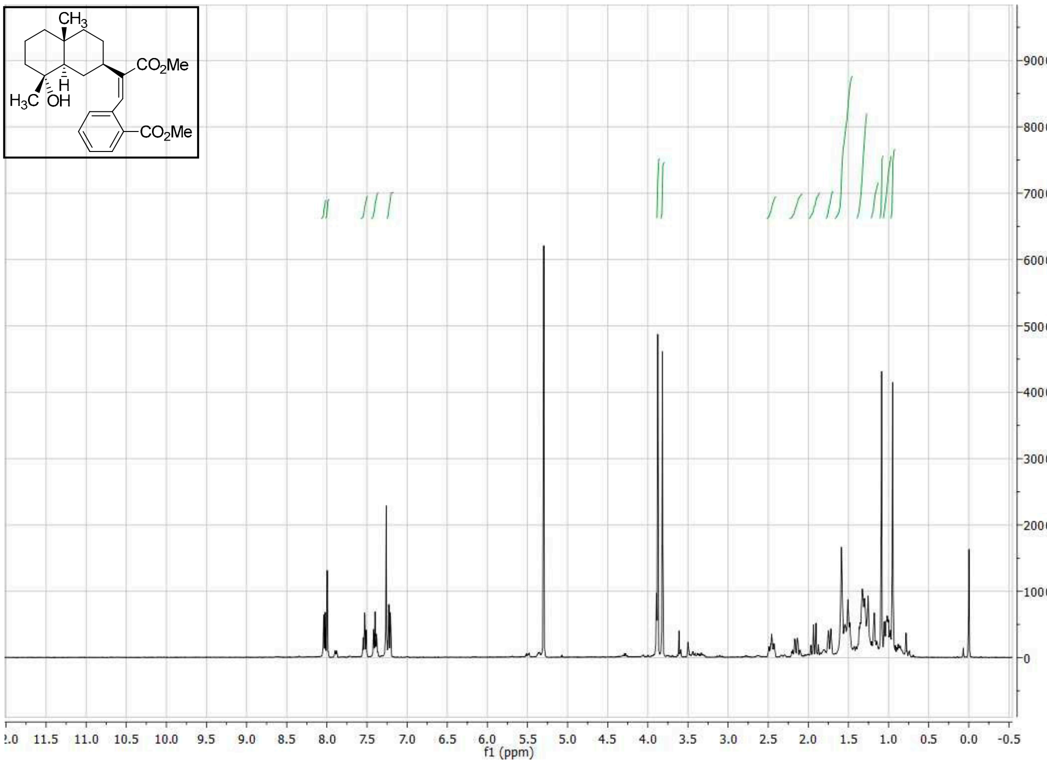


**$^{13}C$ NMR spectrum of methyl 2-[(1E)-2-[(2R,4aR,8R,8aR)-8-hydroxy-4a,8-dimethyl-decahydronaphthalen-2-yl]-3-methoxy-3-oxoprop-1-en-1-yl]benzoate (5h)**

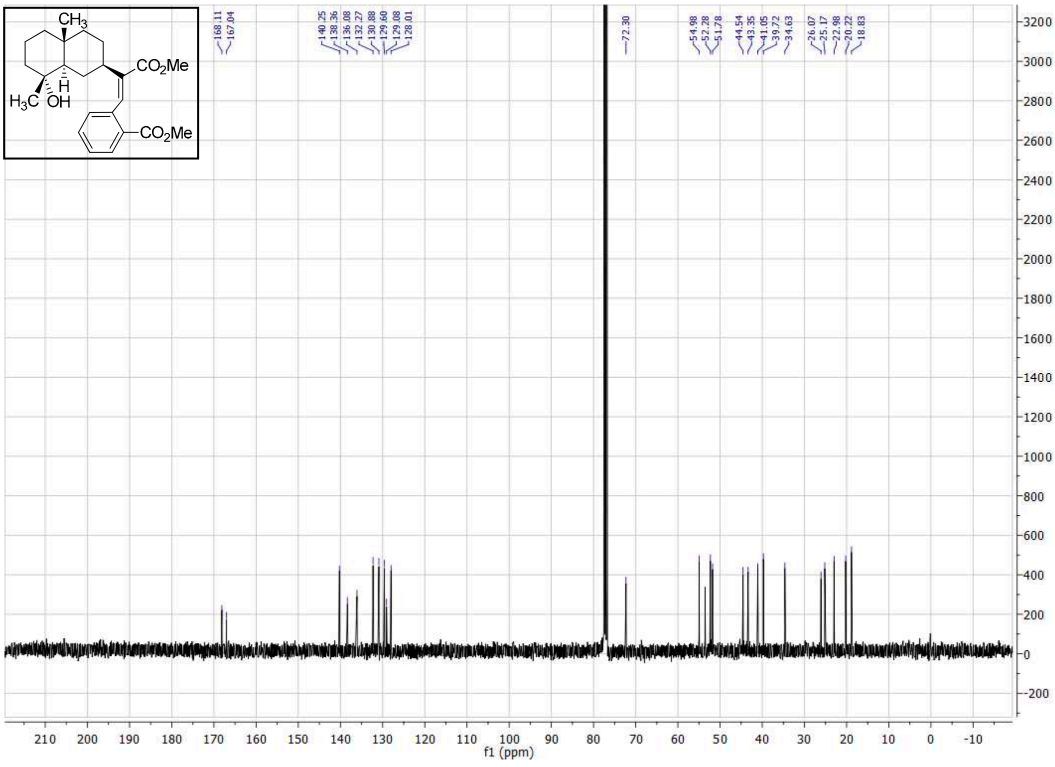

**$^{1}$H NMR spectrum of methyl (2E)-2-[(2R,4aR,8R,8aR)-8-hydroxy-4a,8-dimethyl-decahydronaphthalen-2-yl]-3-{3-methyl-2-oxo-2H,3H-[1,3oxazolo[4,5-*b*]pyridine-6-yl}prop-2-enoate (5i)**

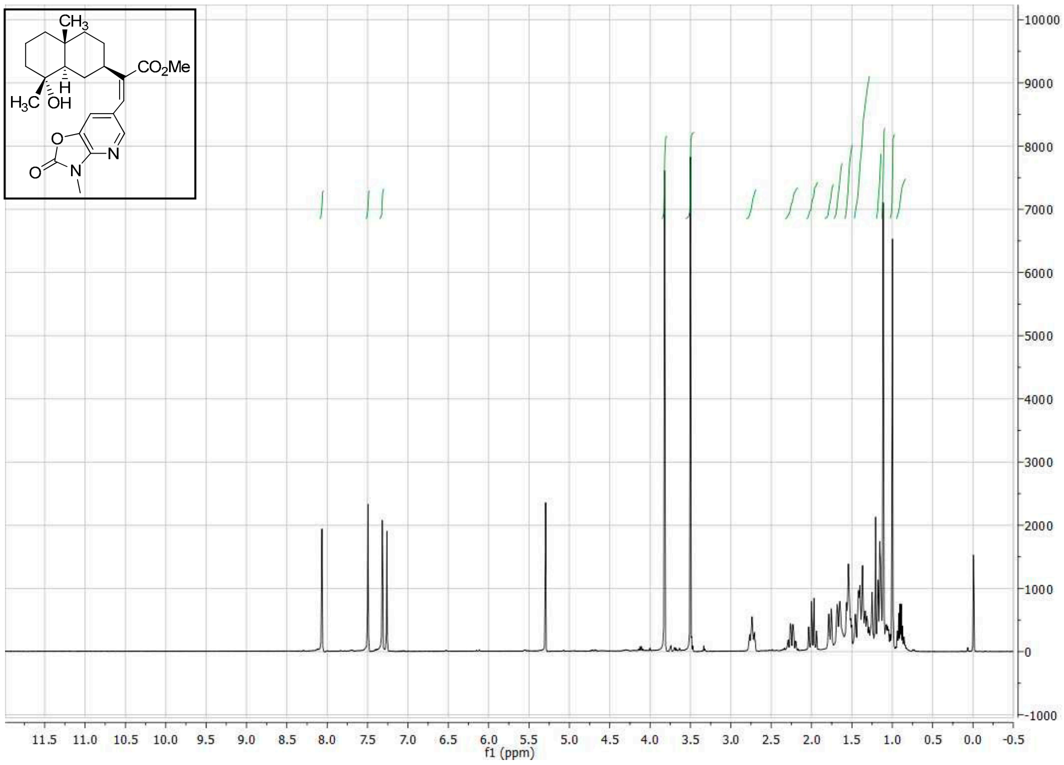


**$^{13}$C NMR spectrum of methyl (2E)-2-[(2R,4aR,8R,8aR)-8-hydroxy-4a,8-dimethyl-decahydronaphthalen-2-yl]-3-{3-methyl-2-oxo-2H,3H-[1,3oxazolo[4,5-*b*]pyridine-6-yl}prop-2-enoate (5i)**

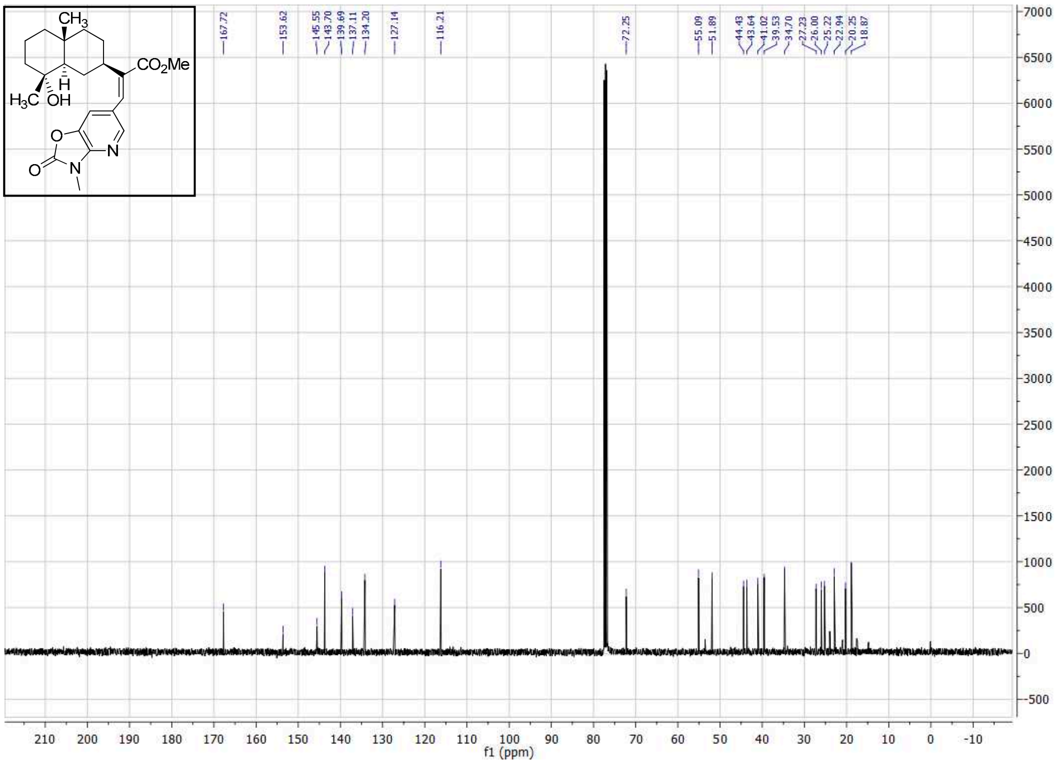

**$^{1}$H NMR spectrum of methyl 2-[(2R,4aS,7R,8aR)-7-hydroxy-4a-methyl-8-methylidene-decahydronaphthalen-2-yl]-3-phenylprop-2-enoate (8a)**

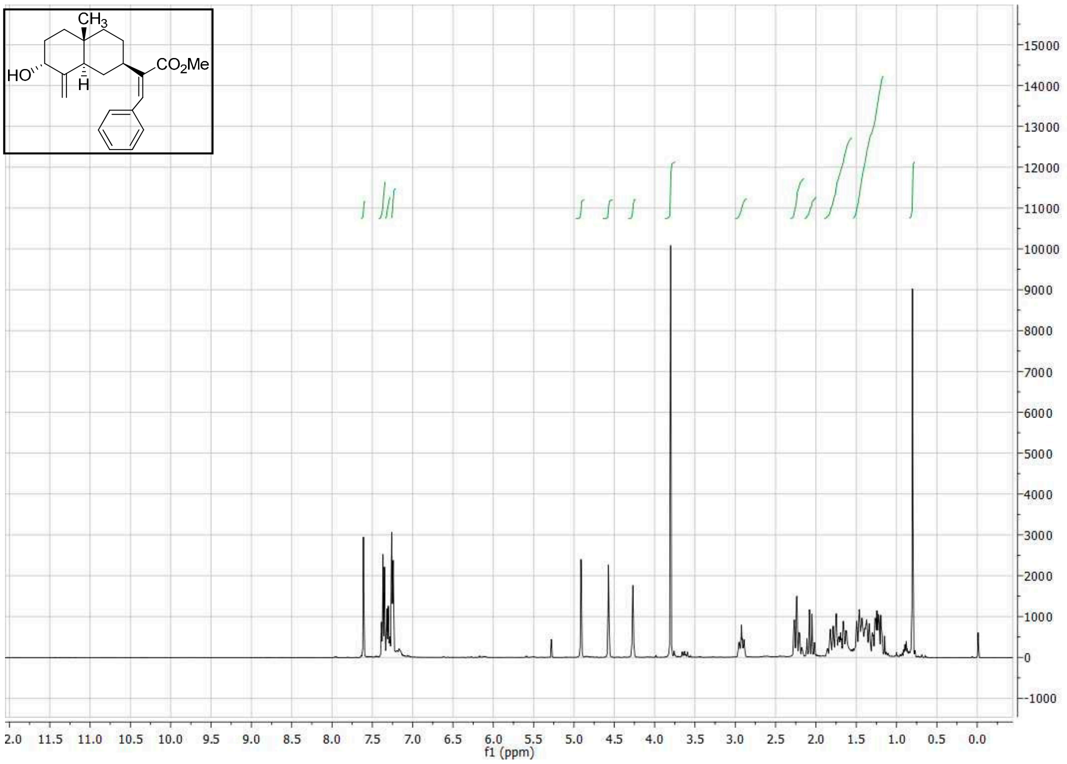


**$^{13}$C NMR spectrum of methyl 2-[(2R,4aS,7R,8aR)-7-hydroxy-4a-methyl-8-methylidene-decahydronaphthalen-2-yl]-3-phenylprop-2-enoate (8a)**

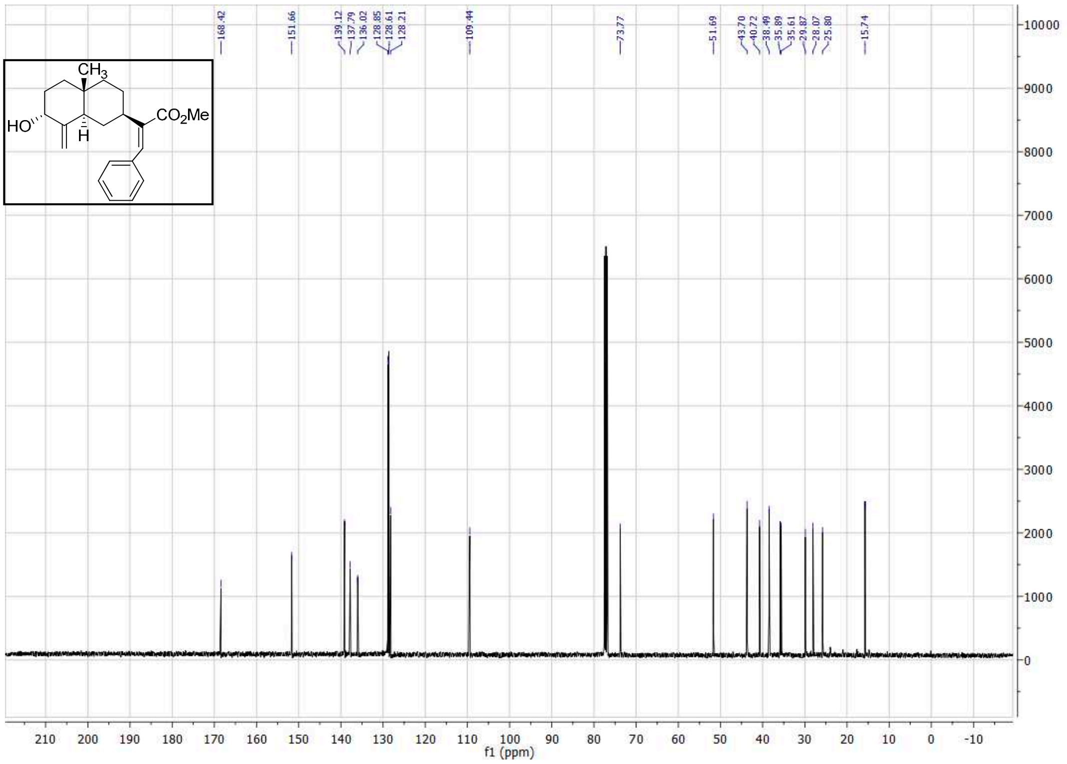

**COSY NMR spectrum of methyl 2-[(2R,4aS,7R,8aR)-7-hydroxy-4a-methyl-8-methylidene-decahydronaphthalen-2-yl]-3-phenylprop-2-enoate (8a)**

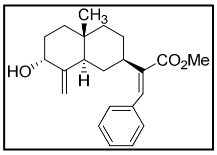


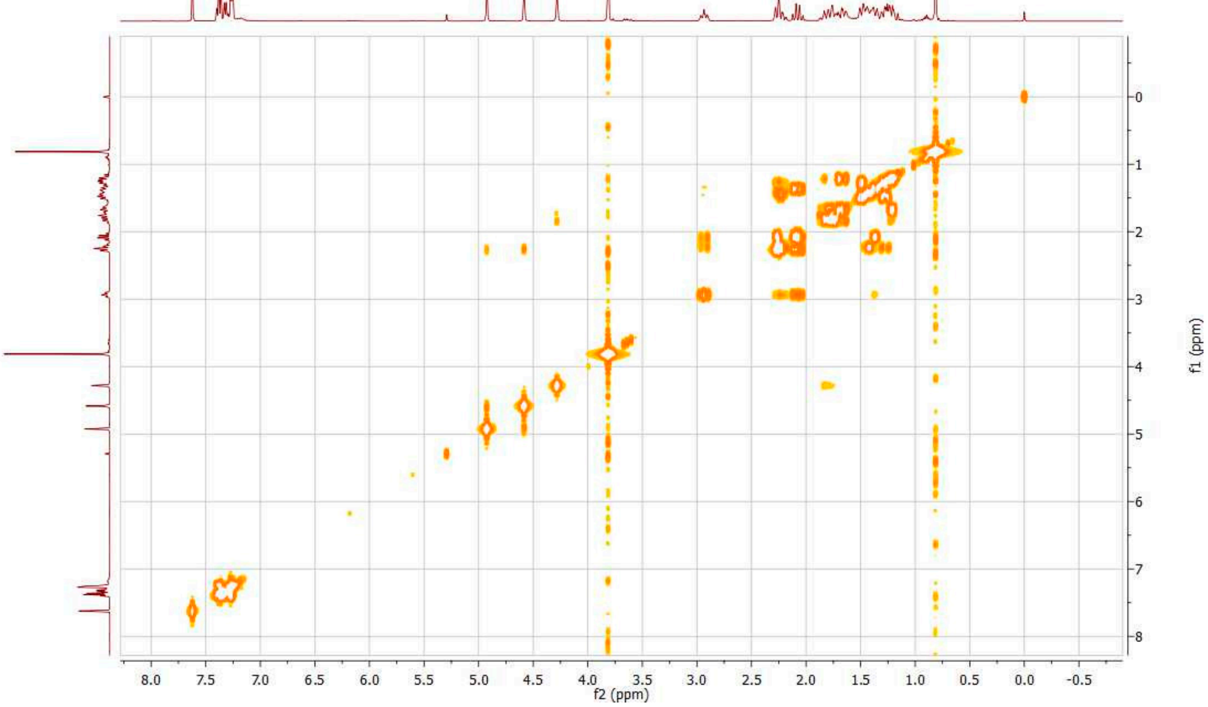

**NOESY NMR spectrum of methyl 2-[(2R,4aS,7R,8aR)-7-hydroxy-4a-methyl-8-methylidene-decahydronaphthalen-2-yl]-3-phenylprop-2-enoate (8a)**

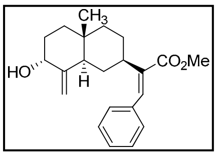


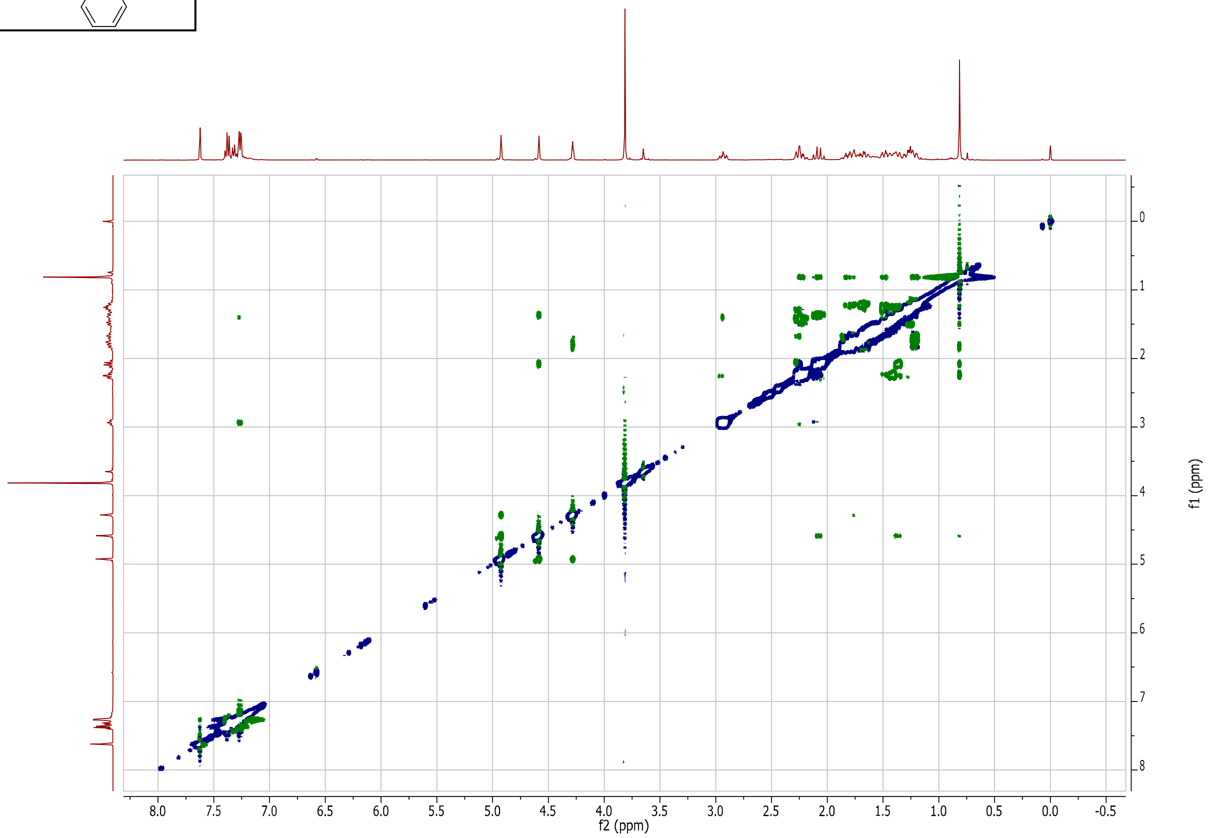

**$^{1}$H NMR spectrum of methyl 2-[(2R,4aS,7R,8aR)-7-hydroxy-4a-methyl-8-methylidene-decahydronaphthalen-2-yl]-3-(4-methylphenyl)prop-2-enoate (8b)**

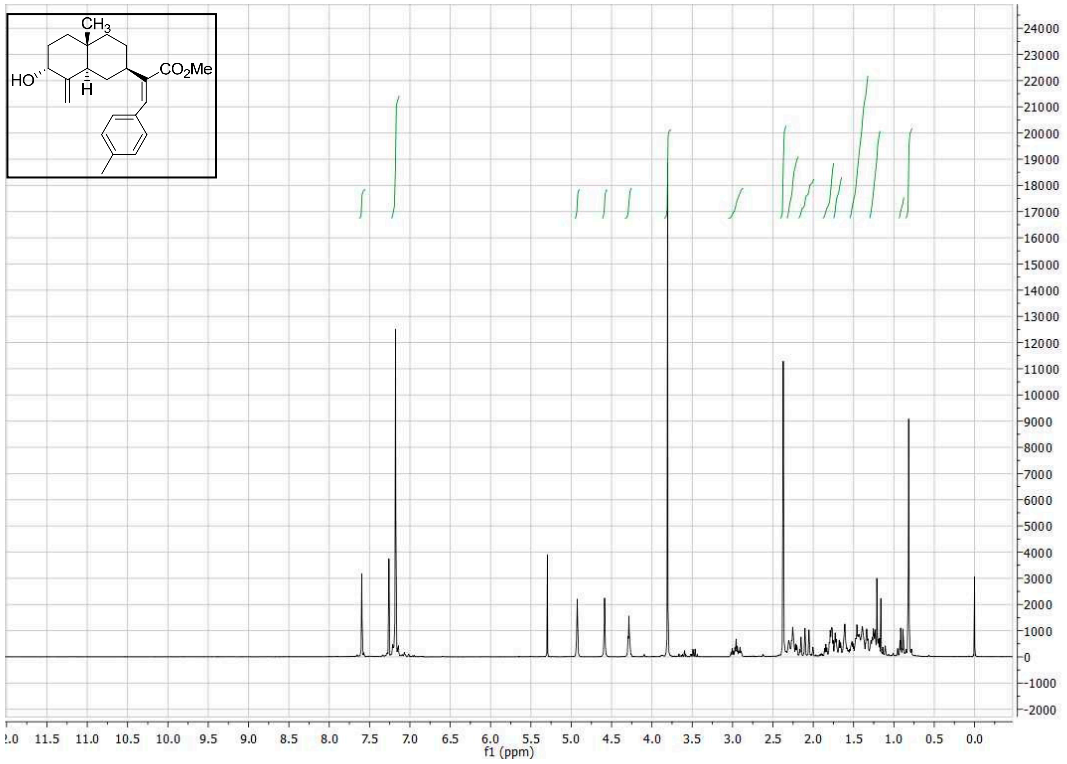


**$^{13}$C NMR spectrum of methyl 2-[(2R,4aS,7R,8aR)-7-hydroxy-4a-methyl-8-methylidene-decahydronaphthalen-2-yl]-3-(4-methylphenyl)prop-2-enoate (8b)**

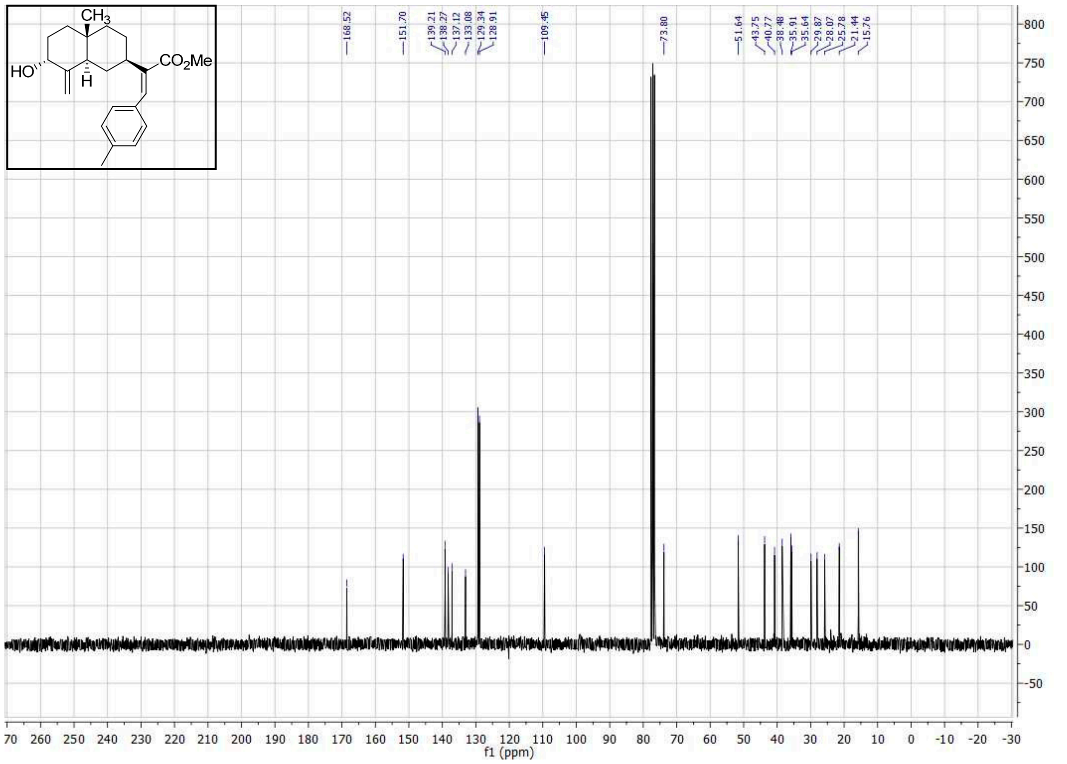

**$^{1}$H NMR spectrum of methyl 2-[(2R,4aS,7R,8aR)-7-hydroxy-4a-methyl-8-methylidene-decahydronaphthalen-2-yl]-3-(4-methoxyphenyl)prop-2-enoate (8c)**

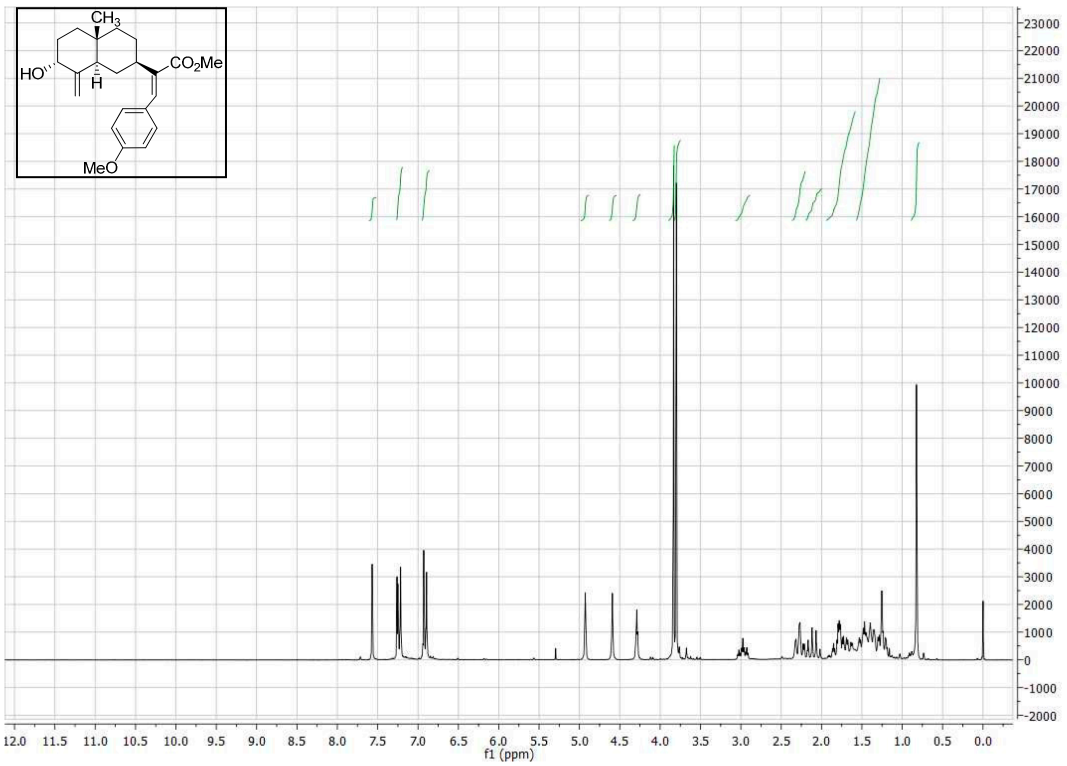

**$^{13}$C NMR spectrum of methyl 2-[(2R,4aS,7R,8aR)-7-hydroxy-4a-methyl-8-methylidene-decahydronaphthalen-2-yl]-3-(4-methoxyphenyl)prop-2-enoate (8c)**

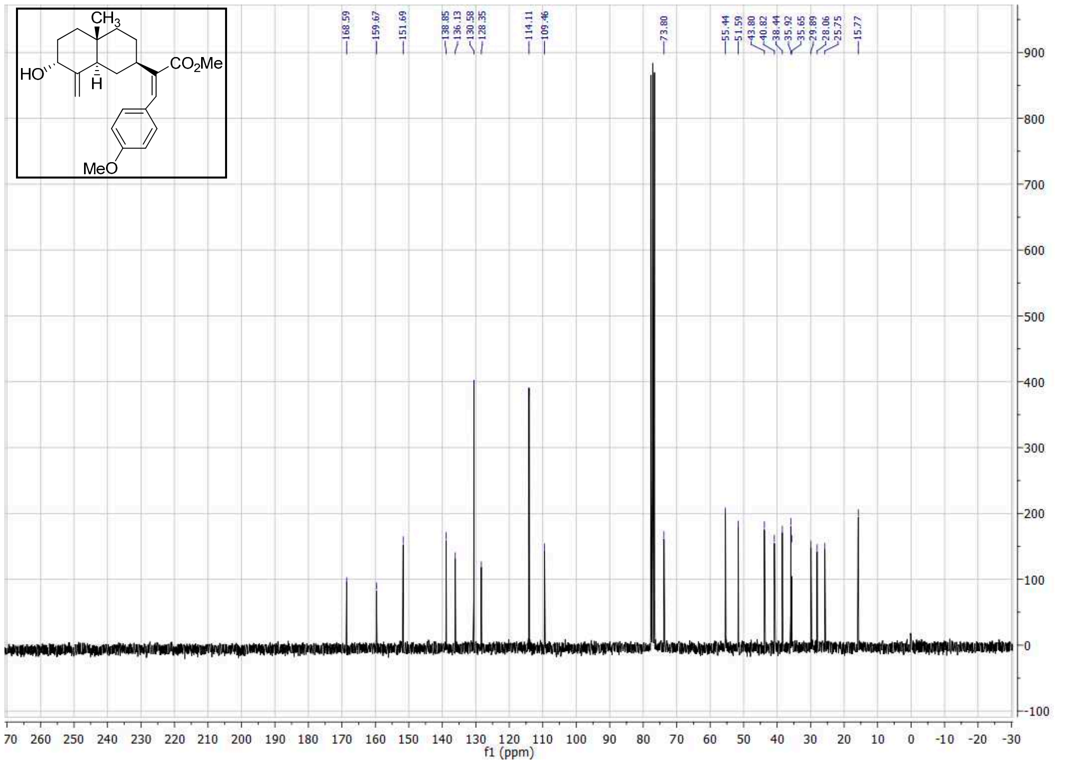

**$^{1}H$ NMR spectrum of methyl 2-[(2R,4aS,7R,8aR)-7-hydroxy-4a-methyl-8-methylidene-decahydronaphthalen-2-yl]-3-(3-methoxyphenyl)prop-2-enoate (8d)**

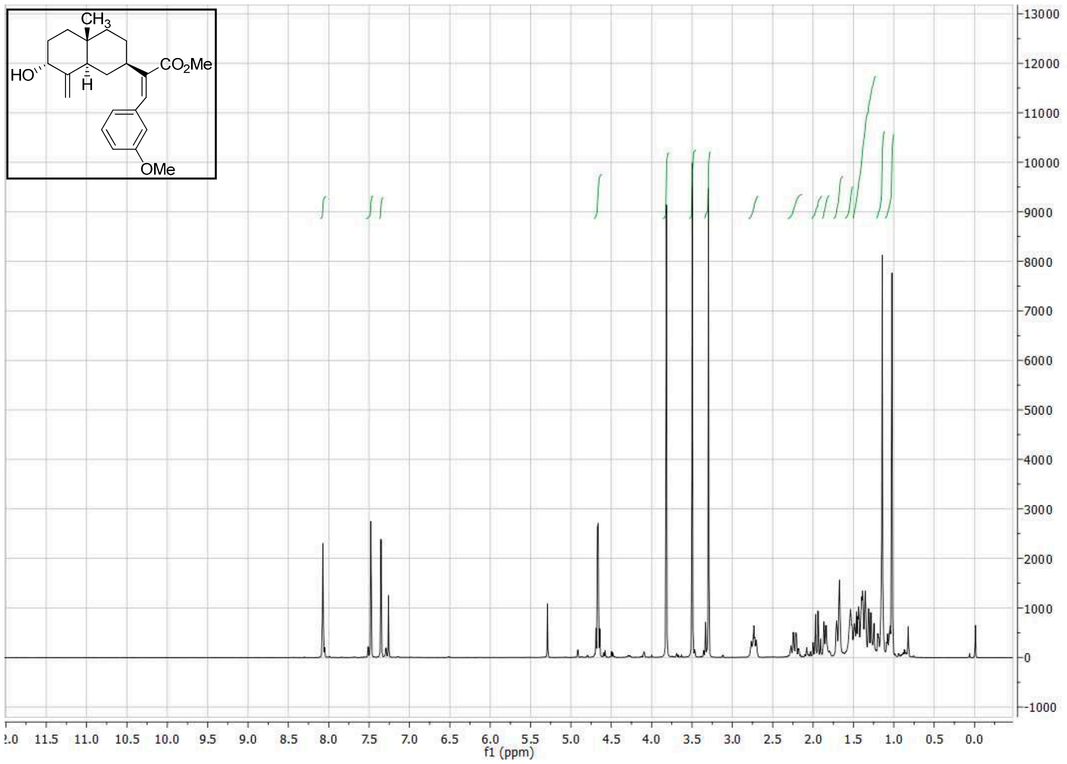


**$^{13}C$ NMR spectrum of methyl 2-[(2R,4aS,7R,8aR)-7-hydroxy-4a-methyl-8-methylidene-decahydronaphthalen-2-yl]-3-(3-methoxyphenyl)prop-2-enoate (8d)**

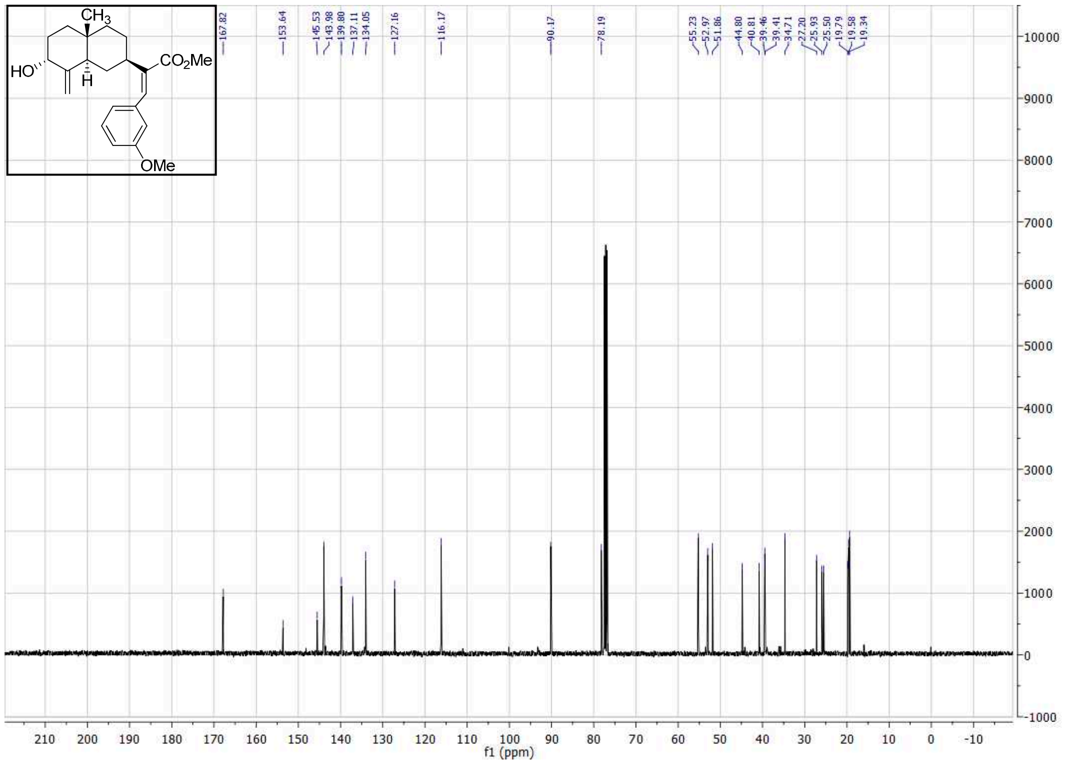

**$^{1}$H NMR spectrum of methyl 2-[(2R,4aS,7R,8aR)-7-hydroxy-4a-methyl-8-methylidene-decahydronaphthalen-2-yl]-3-(2-methylphenyl)prop-2-enoate (8e)**

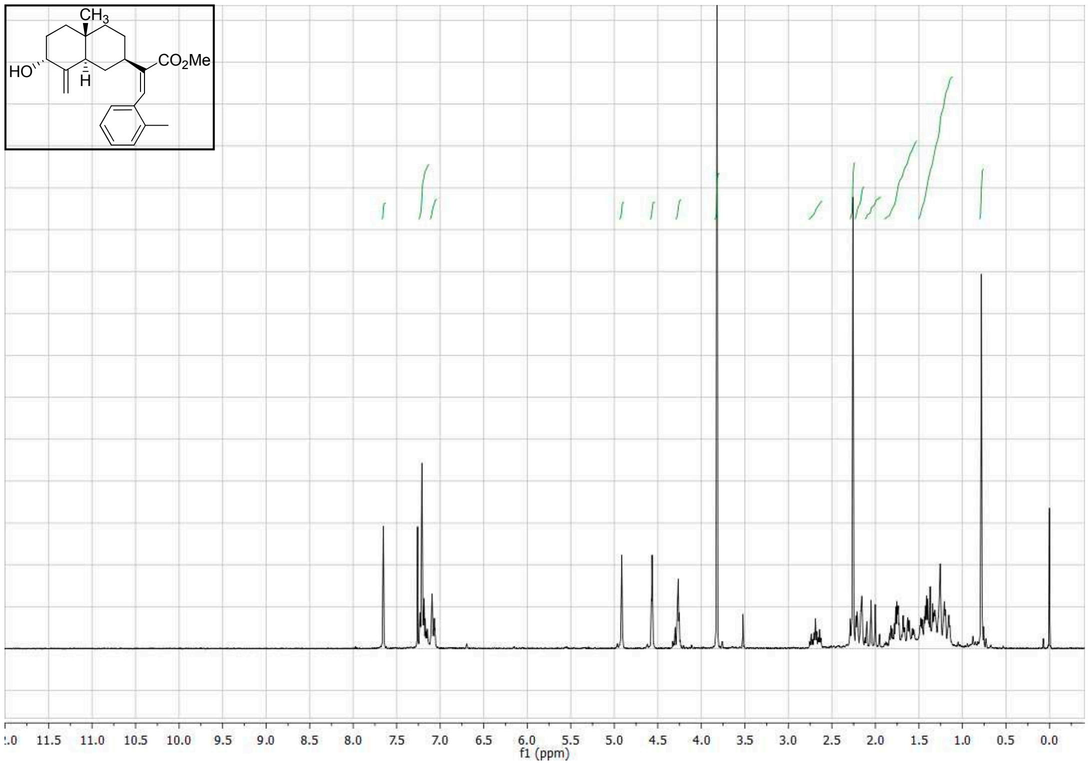


**$^{13}$C NMR spectrum of methyl 2-[(2R,4aS,7R,8aR)-7-hydroxy-4a-methyl-8-methylidene-decahydronaphthalen-2-yl]-3-(2-methylphenyl)prop-2-enoate (8e)**

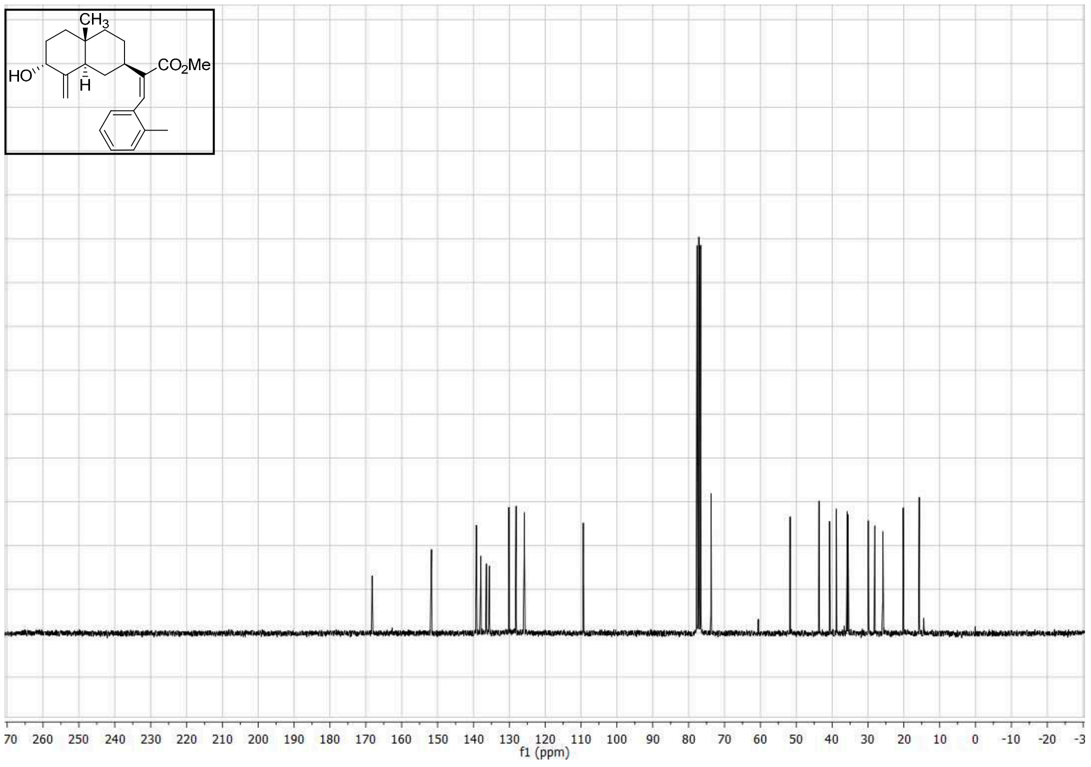

**$^{1}$H NMR spectrum of methyl 2-[(2R,4aS,7R,8aR)-7-hydroxy-4a-methyl-8-methylidene-decahydronaphthalen-2-yl]prop-2-enoate (9a)**

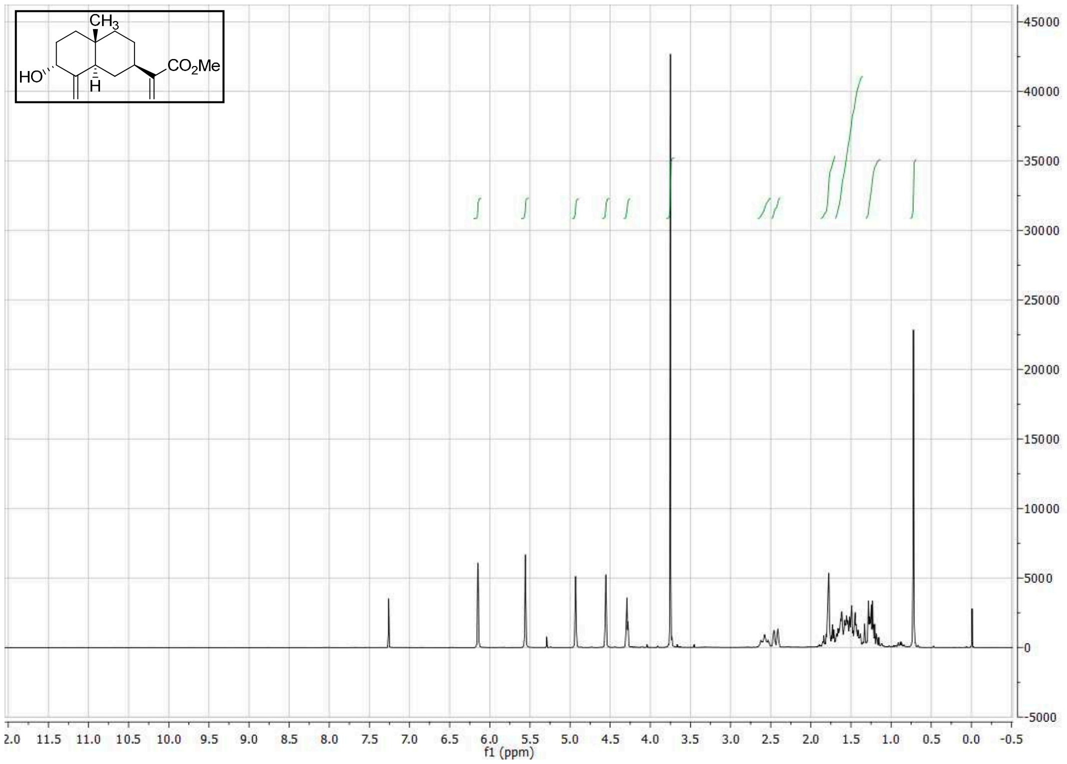

**$^{13}$C NMR spectrum of methyl 2-[(2R,4aS,7R,8aR)-7-hydroxy-4a-methyl-8-methylidene-decahydronaphthalen-2-yl]prop-2-enoate (9a)**

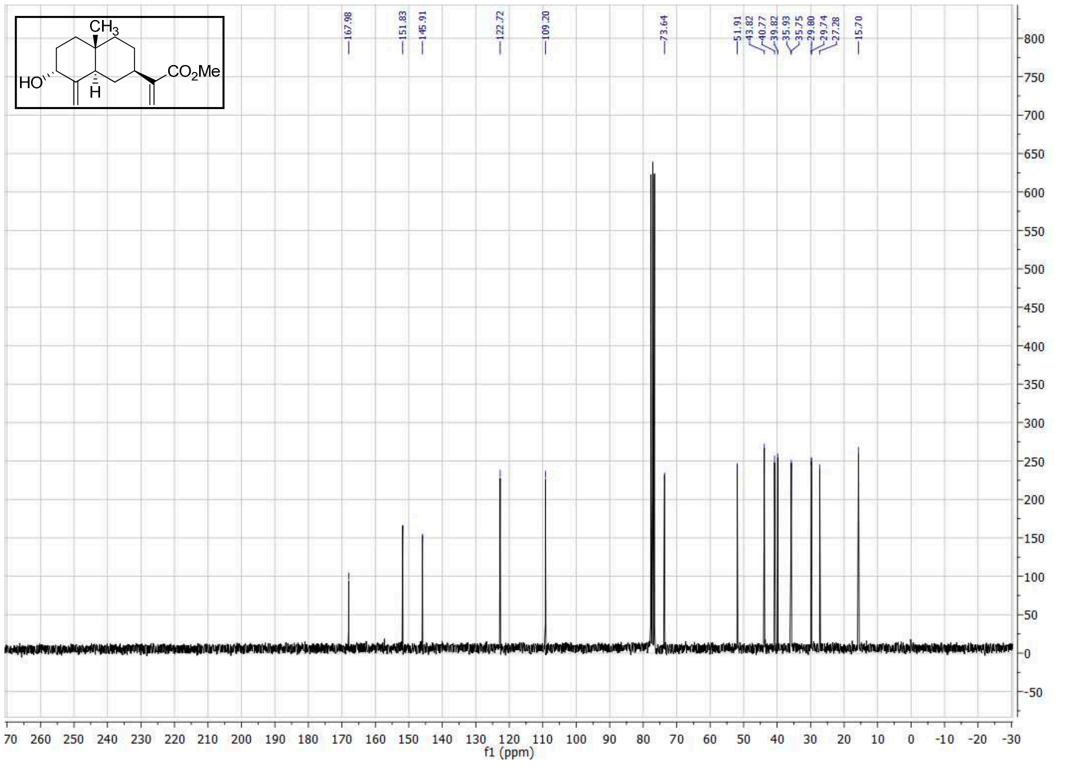

$^{1}$H NMR spectrum of methyl 2-[(2R,4aS,7R)-7-hydroxy-4a,8-dimethyl-1,2,3,4,4a,5,6,7-octahydronaphthalen-2-yl]prop-2-enoate (9b)

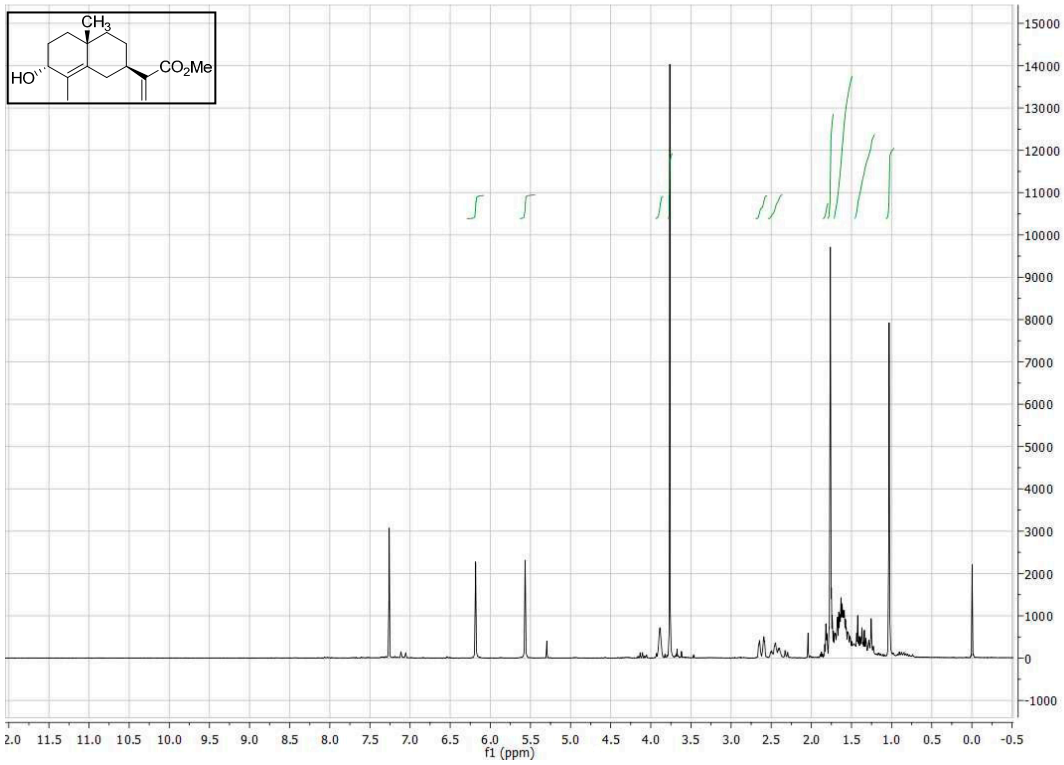


$^{13}$C NMR spectrum of methyl 2-[(2R,4aS,7R)-7-hydroxy-4a,8-dimethyl-1,2,3,4,4a,5,6,7-octahydronaphthalen-2-yl]prop-2-enoate (9b)

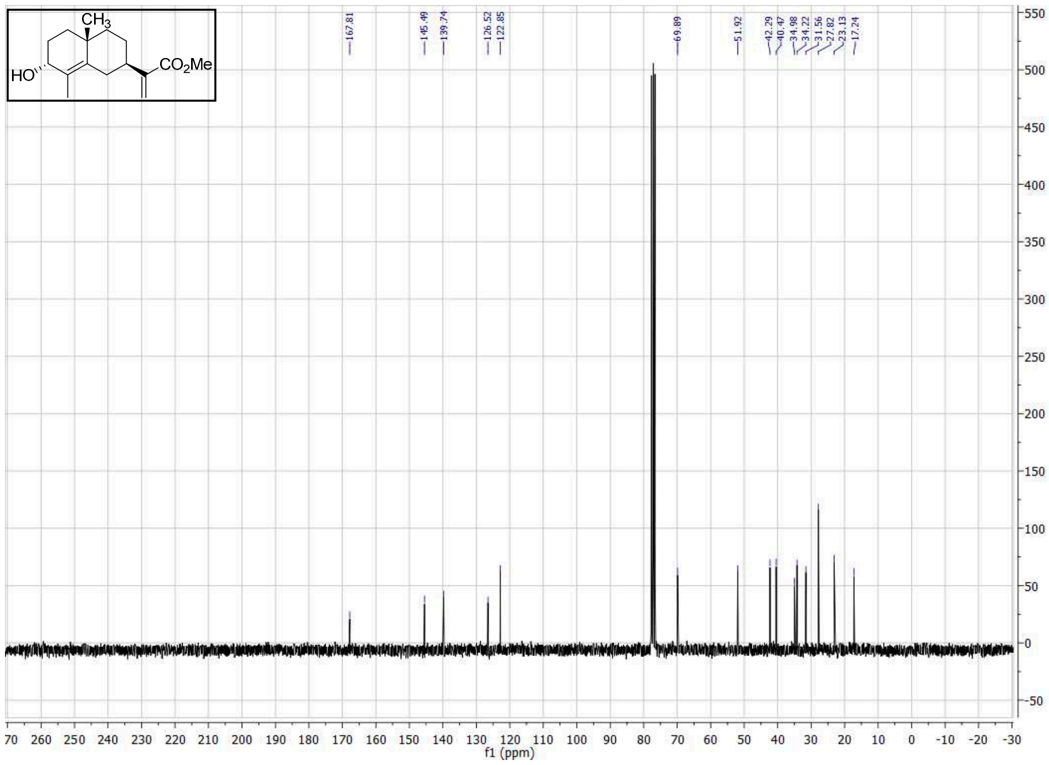

$^{1}H$ NMR spectrum of methyl 2-[(2R,4aS,7R,8aR)-7-(methoxymethoxy)-4a-methyl-8-methylidene-decahydronaphthalen-2-yl]prop-2-enoate (10)

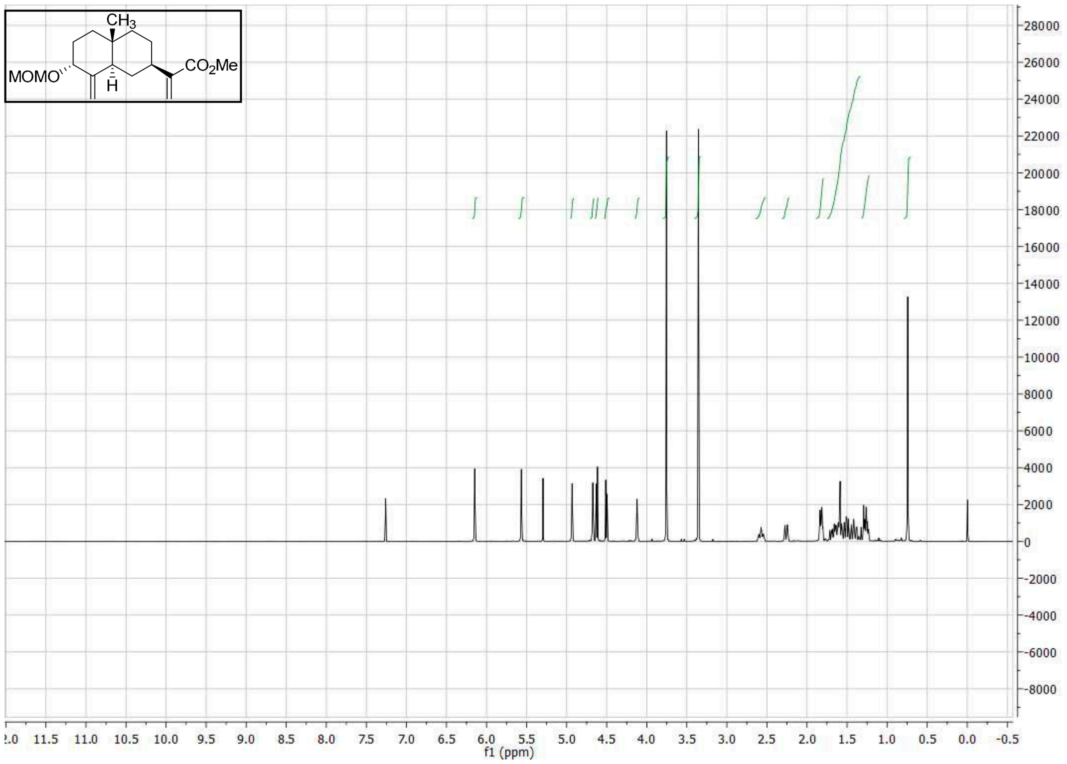


$^{13}C$ NMR spectrum of methyl 2-[(2R,4aS,7R,8aR)-7-(methoxymethoxy)-4a-methyl-8-methylidene-decahydronaphthalen-2-yl]prop-2-enoate (10)

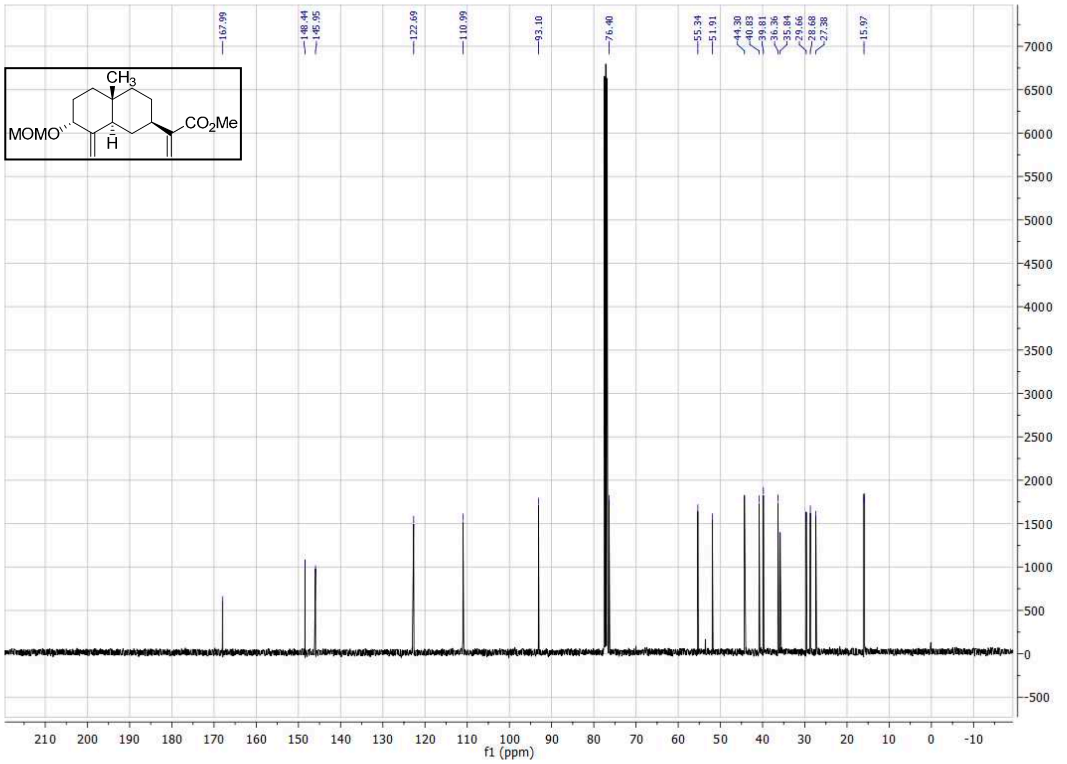

**$^{1}$H NMR spectrum of methyl 2-[(2R,4aS,7R,8aR)-7-(methoxymethoxy)-4a-methyl-8-methylidene-decahydronaphthalen-2-yl]-3-(4-methylphenyl)prop-2-enoate (11)**

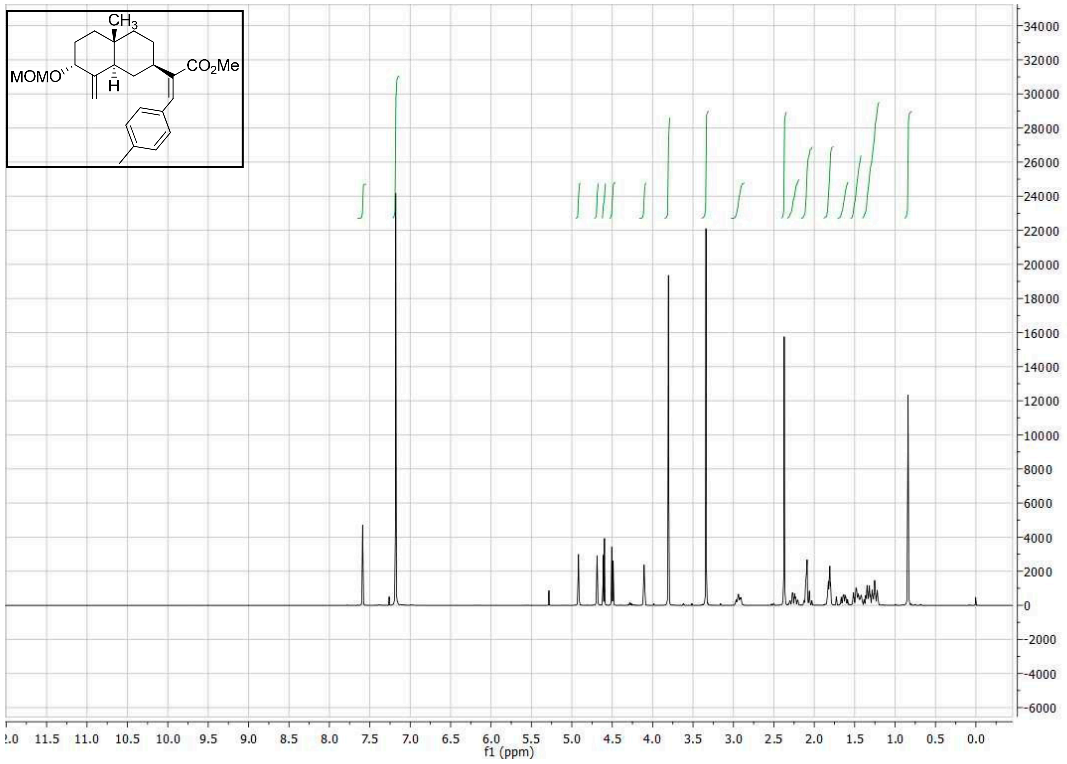


**$^{13}$C NMR spectrum of methyl 2-[(2R,4aS,7R,8aR)-7-(methoxymethoxy)-4a-methyl-8-methylidene-decahydronaphthalen-2-yl]-3-(4-methylphenyl)prop-2-enoate (11)**

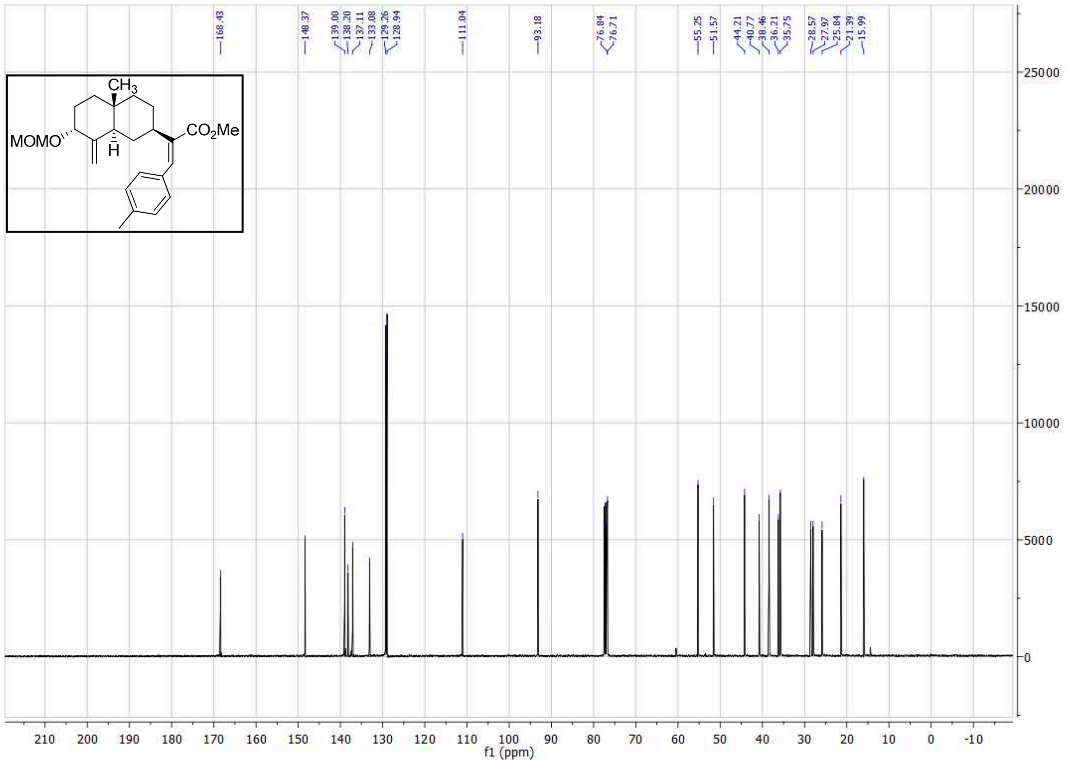

Supplement: Supplementary File 1 [file molecules-22-00652-s001.pdf]
